# Evaluating Machine Learning Models for Molecular Property Prediction: Performance and Robustness on Out-of-Distribution Data

**DOI:** 10.1021/acs.jcim.5c00475

**Published:** 2025-09-15

**Authors:** Hosein Fooladi, Thi Ngoc Lan Vu, Miriam Mathea, Johannes Kirchmair

**Affiliations:** † Department of Pharmaceutical Sciences, Division of Pharmaceutical Chemistry, Faculty of Life Sciences, 27258 University of Vienna, Josef-Holaubek-Platz 2, 1090 Vienna, Austria; ‡ Christian Doppler Laboratory for Molecular Informatics in the Biosciences, Department of Pharmaceutical Sciences, University of Vienna, 1090 Vienna, Austria; § Vienna Doctoral School of Pharmaceutical, Nutritional and Sport Sciences (PhaNuSpo), University of Vienna, 1090 Vienna, Austria; ∥ 5184 BASF SE, Ludwigshafen 67056, Germany

## Abstract

Today, machine learning models are employed extensively to predict the physicochemical and biological properties of molecules. Their performance is typically evaluated on in-distribution (ID) data, i.e., data originating from the same distribution as the training data. However, the real-world applications of such models often involve molecules that are more distant from the training data, necessitating the assessment of their performance on out-of-distribution (OOD) data. In this work, we investigate and evaluate the performance of 14 machine learning models, including classical approaches like random forests, as well as graph neural network (GNN) methods, such as message-passing graph neural networks, across eight data sets using ten splitting strategies for OOD data generation. First, we investigate what constitutes OOD data in the molecular domain for bioactivity and ADMET prediction tasks. In contrast to the common point of view, we show that both classical machine learning and GNN models work well (not substantially different from random splitting) on data split based on Bemis-Murcko scaffolds. Splitting based on chemical similarity clustering (UMAP-based clustering using ECFP4 fingerprints) poses the most challenging task for both types of models. Second, we investigate the extent to which ID and OOD performance have a positive linear relationship. If a positive correlation holds, models with the best performance on the ID data can be selected with the promise of having the best performance on OOD data. We show that the strength of this linear relationship is strongly related to how the OOD data is generated, i.e., which splitting strategies are used for generating OOD data. While the correlation between ID and OOD performance for scaffold splitting is strong (Pearson’s *r* ∼ 0.9), this correlation decreases significantly for all the cluster-based splitting (Pearson’s *r* ∼ 0.4). Therefore, the relationship can be more nuanced, and a strong positive correlation is not guaranteed for all OOD scenarios. These findings suggest that OOD performance evaluation and model selection should be carefully aligned with the intended application domain.

## Graphical abstract

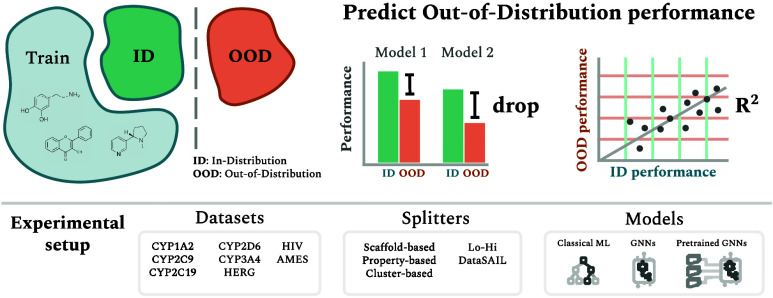

## Introduction

Machine learning (ML) plays a crucial role in the field of drug discovery, allowing the rapid prediction of molecular properties and biological activities.^1–4^ By leveraging vast data sets on chemical compounds, ML models have demonstrated remarkable success in identifying potential drug candidates, optimizing lead compounds, and predicting pharmacokinetic properties. This computational acceleration of early stage drug development has substantially contributed to the reduced need for extensive experimental testing.^5^ Yet, a critical challenge persists in the field: while ML models typically work well on in-distribution (ID) data (i.e., molecules similar to those in their training sets), they are often challenged by out-of-distribution (OOD) data (i.e., molecules that differ substantially from those in their training sets).

ML models generally show significant performance degradation when tested on OOD data and often fail when tested on examples outside the training domain.^6–9^ These limitations are particularly relevant in drug discovery, where exploring novel chemical spaces beyond the boundaries of training data is essential for identifying new therapeutic compounds. Although such exploration is crucial for innovation, it exposes ML models to OOD scenarios where their predictions may be unreliable.^10–12^ The ability to maintain accuracy and robustness when faced with novel molecular structures is, therefore, critical for applications of ML in drug discovery.

This study addresses the challenges posed by OOD data to ML by systematically evaluating the performance of ML models on OOD data in drug discovery, particularly molecular property and activity prediction (Figure 1). We examine a comprehensive range of approaches, from classical tabular methods such as random forests (RF)^13^ operating on molecular fingerprints to message-passing graph neural networks (GNNs)^14^ working directly with molecular graphs. Following recent findings by Gulrajani et al.^15^ that demonstrate the effectiveness of Empirical Risk Minimization (ERM)^16^ compared to specialized domain generalization methods,^17–21^ and given that ERM is the most widely used algorithm for training ML models in cheminformatics, we adopt ERM as our main training algorithm for this benchmark study.

**Figure 1:**
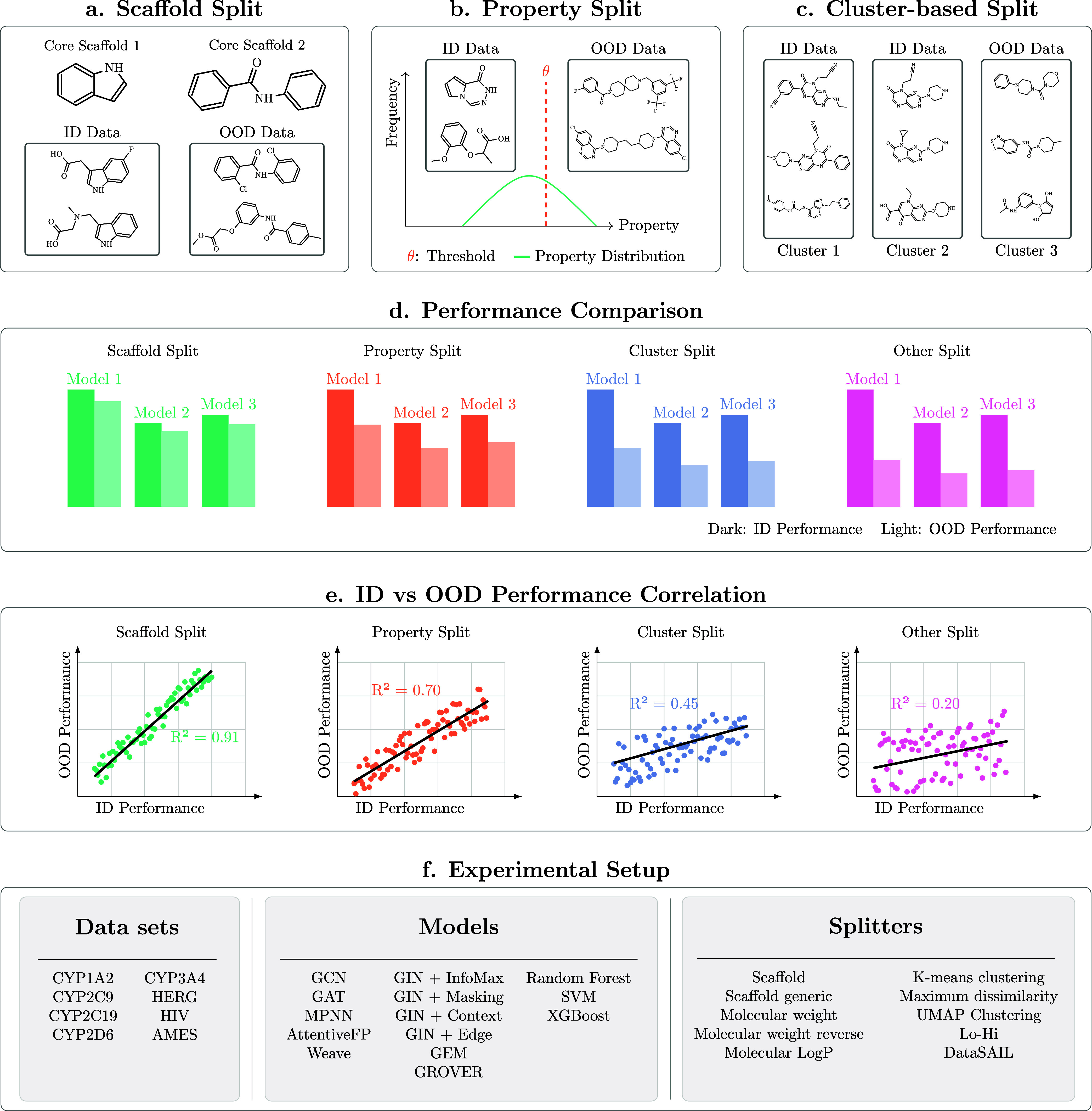
Overview of the study methodology. Data splitting approaches include: (a) scaffold-based splitting, (b) property-based splitting (using molecular weight and LogP), and (c) cluster-based splitting. (d) Performance comparison of classical ML models and GNN-based methods on in-distribution (ID) and out-of-distribution (OOD) test sets. (e) Analysis of linear correlation between ID and OOD performance. (f) Summary of data sets, models, and splitting methods utilized in this study (see Materials and Methods section for more information).

In the first part of this study, we explore what constitutes OOD data in the molecular domain. While some researchers suggest that splitting based on Bemis-Murcko scaffolds^22^ significantly decreases model performance, causing substantial accuracy drops compared to random splitting (as exemplified in the MoleculeNet benchmark^23^), recent studies have questioned this view.^24^ To resolve this ambiguity, we systematically create and evaluate different types of test sets that could potentially be considered OOD, providing a comprehensive analysis of their characteristics and impact on model performance.

In the second part of this study, we investigate the connection between ID and OOD performance. While earlier studies suggest a substantial positive correlation between these metrics, implying that ID performance could serve as a proxy for model selection,^25–27^ recent works have challenged this assumption.^28,29^ Our analysis across various data sets and splitting strategies reveals that the relationship between ID and OOD performance can vary significantly, sometimes even showing negative correlations. These findings suggest that model selection strategies concentrating solely on ID performance may not consistently yield optimal generalization capabilities. More nuanced approaches are therefore needed for the evaluation and selection of models in drug discovery applications.

## Materials and Methods

In this study, we evaluated the performance of three categories of models: (1) classical ML methods (e.g., RF), (2) graph neural networks trained from scratch (GNNs), and (3) pretrained graph neural networks (pretrained GNNs) on both ID and OOD test data sets. When comparing classical approaches to all graph-based approaches collectively, we refer to the latter as GNN-based methods (encompassing both GNNs and pretrained GNNs). We assessed the model performance primarily using the metrics ROC-AUC (reported in the main text) and accuracy (reported in the Supporting Information). Additional metrics relevant to virtual screening, such as hit rate, are reported and discussed in the Results and Discussion section.

The experimental setup consisted of three major components: (1) curated molecular data sets for benchmarking, (2) a selection of ML models, including both GNN-based methods and classical ML approaches, and (3) data splitting methods (splitters) for generating OOD test sets. These components are described in the following sections.

### Data Sets

We extracted our data set from the Therapeutic Data Commons (TDC) database, a benchmark resource for molecular machine learning.^30^ TDC includes data sets for tasks such as small-molecule activity and property prediction, protein–protein interaction prediction, molecular generation, and more. The data sets are organized into three categories: single-instance prediction, multi-instance prediction, and generation. In this study, we utilized the single-instance prediction data sets, specifically focusing on ADMET prediction tasks (which provide data on the absorption, distribution, metabolism, excretion, and toxicity of small molecules) and bioactivity prediction tasks.

For our benchmark study, we applied three key selection criteria to the ADMET and bioactivity prediction tasks:

- **Task Type**: Binary classification tasks
- **Data Set Size**: Data sets were limited to a size range of 5000 to 20,000 data points. This range was chosen to ensure sufficient data is available for reliable model training and evaluation while avoiding large data sets that could lead to increased computational complexity.
- **Class Balance**: The proportion of active compounds in each data set was required to fall between 30 and 70%. This constraint helps maintain a reasonably balanced data set, facilitating more robust training and evaluation.

Eight data sets of the TDC database met these criteria (Table 1), two thereof (CYP2D6 and HIV) after randomly down-sampling the respective majority class (i.e., the inactive compounds, in both cases).

**Table 1:** Data Set Statistics Showing Sample Sizes and Percentage of Active Compounds Before and After Pre-Processing

	data sets size		active (%)	
data sets	original	processed	original	processed
CYP1A2	12,579	12,535	46.3	46.3
CYP2C9	12,092	12,033	33.5	33.5
CYP2C19	12,665	12,613	45.9	46.0
CYP2D6^a^	6514 (13,130)	6493	38.6	38.5
CYP3A4	12,328	12,278	41.5	41.5
HIV^a^	5007 (41,127)	4980	30.0	30.0
AMES	7278	7157	54.6	54.6
HERG	13,445	12,890	49.9	50.0

a Subset extracted from the full data set (original size in parentheses).

The SMILES strings of each molecule in the data sets were preprocessed and standardized to remove salts, correct the protonation states at physiological pH, convert them to canonical tautomers, and apply additional adjustments. Complete preprocessing protocols are detailed in the Supporting Information. After removing duplicates from the standardized SMILES, the final data set sizes used for the benchmark study are listed in Table 1.

Information regarding target types and biological functions for each data set is provided in Table 2. Additional analyses of chemical space visualization and physicochemical property distributions across data sets are available in the Supporting Information (Figures S1–S3, Table S1).

**Table 2:** Description of Data Sets and Their Corresponding Biological Targets Used in This Study^a^

data set	biological relevance	task description
CYP1A2 ^31^	Major drug metabolism enzyme	Binary classification task to predict whether a small molecule is an inhibitor (active) or noninhibitor (inactive) of CYP1A2.
CYP2C9 ^31^	Major drug metabolism enzyme	Binary classification task to predict whether a small molecule is an inhibitor (active) or noninhibitor (inactive) of CYP2C9.
CYP2C19 ^31^	Major drug metabolism enzyme	Binary classification task to predict whether a small molecule is an inhibitor (active) or noninhibitor (inactive) of CYP2C19.
CYP2D6 ^31^	Major drug metabolism enzyme	Binary classification task to predict whether a small molecule is an inhibitor (active) or noninhibitor (inactive) of CYP2D6.
CYP3A4 ^31^	Major drug metabolism enzyme	Binary classification task to predict whether a small molecule is an inhibitor (active) or noninhibitor (inactive) of CYP3A4.
HIV^23^	Established antiviral drug target	Binary classification task to identify compounds with antiviral activity against HIV.
AMES^32^	Established toxicity end point	Binary classification task to predict whether a compound is likely mutagenic (positive in the Ames test) or nonmutagenic (negative in the Ames test).
HERG^33^	Established toxicity end point	Binary classification task to predict whether a compound is a hERG channel blocker (active) or not (inactive).

a Data sourced from the Therapeutic Data Commons (TDC) database.^30^

### Machine Learning Models

Both GNN-based methods and classical ML models were evaluated. Specifically, we explored 11 different GNN-based architectures: graph convolutional network (GCN),^34^ graph attention network (GAT),^35^ message passing neural network (MPNN),^36^ AttentiveFP,^37^ Weave,^38^ four variants of the graph isomorphic network (GIN)^39^ pretrained using different strategies: deep graph infomax, attribute masking, context prediction, and edge prediction,^40^ geometry-enhanced molecular representation learning (GEM),^41^ and self-supervised message passing graph transformer (GROVER).^42^ For classical ML models, we selected RF,^13^ support vector machine (SVM),^43,44^ and extreme gradient boosting (XGBoost)^45^ due to their popularity and established efficacy in cheminformatics. These models are among the most widely used for tasks such as molecular property prediction and bioactivity classification. (Table 3)

**Table 3:** Machine Learning Models Employed in This Study

model	model type	implementation
GCN	GNN	DGL-LifeSci
GAT	GNN	DGL-LifeSci
MPNN	GNN	DGL-LifeSci
AttentiveFP	GNN	DGL-LifeSci
Weave	GNN	DGL-LifeSci
GIN + InfoMax^a^	Pretrained GNN	DGL-LifeSci
GIN + Masking^b^	Pretrained GNN	DGL-LifeSci
GIN + Context^c^	Pretrained GNN	DGL-LifeSci
GIN + Edge^d^	Pretrained GNN	DGL-LifeSci
GEM^e^	Pretrained GNN	PaddlePaddle
GROVER^f^	Pretrained GNN	PyTorch
Random Forest	Classical ML	scikit-learn
SVM	Classical ML	scikit-learn
XGBoost	Classical ML	XGBoost

a Deep graph infomax pretraining.

b Attribute masking pretraining.

c Context prediction pretraining.

d Edge prediction pretraining.

e Geometry-enhanced molecular representation learning with 3D spatial structure.

f Self-supervised message passing graph transformer.

Since the focus of our work is on comparing model performance between ID and OOD test sets rather than optimizing performance, we used fixed hyperparameters across all model architectures to ensure fair comparisons. The specific hyperparameters used in our benchmark study are available in Table S2. A comprehensive description of hyperparameter selection and the model training procedure is provided in the Supporting Information.

#### Graph Neural Network (GNN) Based Methods

Molecules can be represented as graphs, where atoms form the nodes and bonds are the edges of the graph. Graph G=(V,E) is an object with a set of nodes $v_{i}\in V$ , a set of edges $\left(v_{i},v_{j}\right)\in E\subset V\times V$ , an adjacency matrix $A\in R^{N\times N}\left(\left|V\right|=N\right)$ , and a degree matrix **D**_*ii*_ = ∑_*j*_ *A*_*ij*_. In attributed graphs, each node $v_{i}\in V$ is represented by a feature vector $x_{i}\in R^{D}$ , with the node feature matrix $X\in R^{N\times D}$ over all the nodes, and optionally, each edge is represented by a feature vector $e_{i,j}\in R^{D^{\prime }}$ .

GNNs operate on these representations, updating features at each layer to a new node feature matrix $H\in R^{N\times F}$ , and possibly (optionally) a new edge feature matrix $E\in R^{M\times F^{\prime }}$ . GNNs and pretrained GNNs implementations were sourced from the DGL-LifeSci Python package,^46^ the GEM GitHub repository, and the GROVER GitHub repository.

GCN, GAT, and all GIN models utilize only node features and do not consider edge features. In contrast, Weave, MPNN, AttentiveFP, GEM, and GROVER models incorporate both node and edge features in their update functions. The GCN, GAT, MPNN, Weave, and AttentiveFP models use the same set of node features for each atom in the graph.

For the pretrained GIN models, we use the features generated through pretraining for each atom. For all the pretrained GIN models, the backbone architecture is GIN. GEM adapts a Geometry-based Graph Neural Network (GeoGNN) that incorporates 3D molecular geometry through a dual-graph architecture with GIN-style aggregation and update functions. GROVER integrates message-passing networks with Transformer-style architecture, using dynamic message passing where the number of hops is randomized during training.

Details on each model’s node and edge features can be found in the Supporting Information Table S3.

All of the GNN-based methods in this work follow the message passing or neighborhood aggregation scheme for updating each layer,

1 hi(L)=Update(L)(hi(L−1),AGGREGATE(L)(hi(L−1),hj(L−1),ei,j))=γ(L)(hi(L−1),⊕j∈N(i)φ(L)(hi(L−1),hj(L−1),ei,j))=γ(L)(hi(L−1),mN(i)(L))

where ⊕ denotes a differentiable, permutation invariant aggregation function, e.g., sum, mean or max, γ (Update) and ϕ denote differentiable functions such as multilayer perceptrons (MLPs), and $m_{N\left(i\right)}$ is the message that is aggregated from *v*_*i*_’s graph neighborhood.

Recent advances have extended the basic message passing framework in two notable directions. GEM incorporates 3D molecular geometry by operating on two interconnected graphs: an atom-bond graph G=(V,E) where atoms form nodes and bonds form edges, and a bond-angle graph H=(E,A) where bonds become nodes and bond angles become edges. This dual-graph approach enables the model to capture both topological and spatial relationships. GROVER enhances message passing by integrating it with Transformer-style attention mechanisms, where GNN outputs serve as queries, keys, and values for multihead attention, enabling both local structural information capture and global relation extraction.

Table 4 presents the message passing updating rules for node features in GNNs. In this Table, *Ã* = *A* + *I*_*N*_ is the adjacency matrix of the undirected graph G with added self-connections, and *d̂*_*i*_ and *d̂*_*j*_ are the degrees of nodes i and j in this adjacency matrix *Ã. σ* indicates nonlinearity, **W** are learnable parameters, GRU stands for gated recurrent unit, ϵ is a tunable scaler, *K* represents the number of heads for multihead attentions, and ∥ indicates the concatenation operation.

**Table 4:** Update Equations for Different Graph Neural Network Architectures

model	update equation
GCN	$h_{i}^{\left(L\right)}=\sigma \left(\sum_{j\in N\left(i\right)\cup \left\{i\right\}}\frac{1}{\sqrt{{\mathrm{d̂}}_{i}{\mathrm{d̂}}_{j}}}W^{\left(L\right)}h_{j}^{\left(L-1\right)}\right)$
GAT	$h_{i}^{\left(L\right)}={∥}_{k=1}^{k=K}\sigma \left(\sum_{j\in N\left(i\right)\cup \left\{i\right\}}\alpha_{i,j_{k}}^{L}W_{k}^{\left(L\right)}h_{j}^{\left(L-1\right)}\right)$
	$\alpha_{i,j_{k}}^{L}={\mathrm{softmax}}_{j}\left({e_{i,j}}_{k}\right)$ ,
	where ${e_{i,j}}_{k}=\mathrm{LeakyReLU}\left(a^{T}\left[W_{k}^{\left(L\right)}h_{i}∥W_{k}^{\left(L\right)}h_{j}\right]\right)$
MPNN	$h_{i}^{\left(L\right)}=\mathrm{Update}\left(h_{i}^{\left(L-1\right)},\sum_{j\in N\left(i\right)}\phi^{L}\left(h_{i}^{\left(L-1\right)},h_{j}^{\left(L-1\right)},e_{i,j}^{\left(L-1\right)}\right)\right)$
Weave	$h_{i}^{\left(L\right)}={\mathrm{Update}}_{\mathrm{atom}}\left(h_{i}^{\left(L-1\right)},⊕\left(\{{\mathrm{WeaveMessage}}_{j\to i}^{\left(L\right)}{\}}_{j\in N\left(i\right)}\right)\right)$
	${\mathrm{WeaveMessage}}_{j\to i}^{\left(L\right)}=\mathrm{MLP}\left(h_{i}^{\left(L-1\right)},h_{j}^{\left(L-1\right)},e_{i,j}^{\left(L-1\right)}\right)$
AttentiveFP	$h_{i}^{\left(L\right)}=\mathrm{GRU}\left(h_{i}^{\left(L-1\right)},\sum_{j\in N\left(i\right)}\alpha_{i,j}W^{\left(L\right)}h_{j}^{\left(L-1\right)}\right)$
	where $\alpha_{i,j}={\mathrm{softmax}}_{j}\left(\mathrm{MLP}\left([h_{i}∥h_{j}]\right)\right)$
GIN	$h_{i}^{\left(L\right)}={\mathrm{MLP}}^{\left(L\right)}\left(\left(1+\epsilon^{\left(L\right)}\right)h_{i}^{\left(L-1\right)}+\sum_{j\in N\left(i\right)}h_{j}^{\left(L-1\right)}\right)$

After updating node representations, node features are aggregated to obtain a graph representation, which is then fed into a predictor (often an MLP) for final prediction. The exact hyperparameters used for all experiments with GNNs and pretrained GNNs are reported in Table S2. Additionally, the process, data sets, and tasks for pretraining GNNs are described in the Supporting Information.

#### Classical Machine Learning Models

The implementations of RF^13^ and SVM^43,44^ were taken from the widely used Python library, scikit-learn,^47^ while the XGBoost implementation was obtained from the XGBoost Python library.^45^ To represent molecular structures for input to these algorithms, we utilized Morgan fingerprints ECFP4 with a radius of 2 and a bit length of 2048. Detailed hyperparameters for each method can be found in the Supporting Information Table S2.

### Splitters

Ten different splitters were employed to assign compounds to the OOD test and ID data sets (Table 5). Additionally, a random splitter was used as a reference.

**Table 5:** Overview of Data Set Splitting Strategies

splitting method	description
Scaffold	(1) Generate Bemis-Murcko scaffolds^22^ for all compounds with RDKit (MurckoScaffold.GetScaffoldForMol)
	(2) Group compounds sharing identical scaffolds
	(3) Place compounds with the same scaffold in the same subset (either OOD test or ID data set)
Scaffold Generic	Same as scaffold split, utilizing generic Bemis-Murcko scaffolds (all atoms converted to carbon atoms, all bonds to single bonds)
Molecular Weight^a^	(1) Calculate molecular weight for each compound using RDKit (rdMolDescriptors.CalcExactMolWt)
	(2) Sort compounds by molecular weight values
	(3) Assign the top 20% (highest weight) to the OOD test set and the remaining 80% to the ID data set
Molecular Weight Reverse^a^	Same as molecular weight splitting, just in step 3, assign the bottom 20% (lowest weight) to the OOD test set and the remaining 80% to the ID data set
Molecular LogP^a^	(1) Calculate CLogP for each compound using RDKit (Descriptors.MolLogP)
	(2) Sort compounds by CLogP values
	(3) Assign the top 20% (highest CLogP) to the test set and the remaining 80% to the ID data set
K-means Clustering^b^	(1) Generate ECFP4 fingerprints (radius = 2, 2048 bits) $M\in R^{N\times 2048}$
	(2) Create distance matrix $M\in R^{N\times 512}$ using 512 randomly selected molecules (Tanimoto distance of each compound to these 512 selected molecules). This step transform binary to continuous representation
	(3) Apply K-means clustering using Euclidean distance
	(4) Randomly assign 20% of clusters to the OOD test set and the remainder to the ID data set
Max Dissimilarity	(1) Perform K-means clustering
	(2) Select the most distant cluster for the OOD test set
	(3) Choose the furthest cluster from the OOD test set as the initial ID cluster
	(4) Iteratively add nearest clusters to ID data set until 80% allocation
	(5) Assign remaining clusters to the OOD test set
UMAP Clustering^48^	(1) Generate ECFP4 fingerprints (radius = 2, 2048 bits) $M\in R^{N\times 2048}$
	(2) Apply UMAP (Uniform Manifold Approximation and Projection) dimensionality reduction to preserve local and global structure and create $M\in R^{N\times 2}$ embedding matrix
	(3) Perform agglomerative clustering on the reduced UMAP embedding space
	(4) Assign clusters to OOD test set (20%) and ID data set (80%) to maximize distributional shift between training and test molecules
Lo-Hi^a^ ^,^ ^c^ ^49^	(1) Build a similarity graph: Connect molecules with ECFP4 (radius = 2, 1024 bits) Tanimoto similarity >0.4 (typically results in one giant connected component containing 95% of molecules)
	(2) Apply clustering: Use Butina clustering to create a computationally tractable coarsened graph (optional)
	(3) Formulate optimization: Set up Integer Linear Programming (ILP) to minimize removed molecules while breaking connectivity
	(4) Solve ILP: Find the optimal set of molecules to remove that eliminates all similarity connections between partitions
	(5) Create a split: Assign remaining molecules to ID data set and OOD test set, ensuring no test molecule has similarity >0.4 to ID data set
DataSAIL^d^ ^50^	(1) Compute similarity matrix: Calculate pairwise Tanimoto similarities using ECFP4 (radius *r* = 2, 1024 bits) of molecules (optionally of BemisMurcko scaffolds)
	(2) Precluster for scalability: Apply spectral clustering (similarities) or agglomerative clustering (distances) to group molecules into 50 clusters
	(3) Formulate Integer Linear Programming (ILP): Set up optimization routine to minimize total similarity between entities assigned to different splits while respecting size constraints
	(4) Solve the ILP: Solve the ILP using an off-the-shelf ILP solver (e.g., GUROBI, MOSEK, SCIP) via CVXPY python package.
	(5) Map back to the original data: Extract final split assignments from cluster assignments to individual molecules

a Deterministic splitter: Repeated use of any such method produces identical splits.

b Implementation originating from the Splito Python package.

c For Lo-Hi splitting. The ”Hi” split variant is used in this study.

d For DataSAIL splitting. The S1 method (i.e., the similarity-based one-dimensional cold split method) is employed in this study.

Four of the splitting methods, i.e., molecular weight, molecular weight reverse, molecular LogP, and Lo-Hi, are deterministic. This means that repeated use of these methods produces identical splits. The remaining splitting strategies are stochastic, meaning that repeating the splitting process can result in varying subsets for the OOD and ID data sets. Parameters for each splitting method are available in Table S4.

### Overall Modeling Workflow for Comparing In-Distribution and Out-of-Distribution Performance

Each data set *D* = {(*x*_*i*_, *y*_*i*_)}_*i* = 1_^*n*^ is initially divided into two parts: an out-of-distribution (OOD) test set and an in-distribution (ID) data set, using various extrapolative splitting approaches (detailed in Splitters). To minimize label distribution shifts between ID and OOD sets while maintaining feature distribution differences (covariate shift), we implement an iterative splitting process. This process generates up to 100 candidate splits and selects those where the difference in the percentage of active compounds between ID and OOD sets falls below a predetermined threshold. We aim to obtain 10 qualifying splits, incrementally relaxing the threshold if necessary. For deterministic splitting methods, where controlling label distribution is not possible, we accept the resulting label distribution shift (Figure 2).

2 $$\begin{array}{l} P_{\left(X,Y\right)}^{\mathrm{OOD}}\left(x,y\right)\neq P_{\left(X,Y\right)}^{\mathrm{ID}}\left(x,y\right)\\ P_{Y}^{\mathrm{OOD}}\left(y\right)\approx P_{Y}^{\mathrm{ID}}\left(y\right)\\ P_{X}^{\mathrm{OOD}}\left(x\right)\neq P_{X}^{\mathrm{ID}}\left(x\right) \end{array}$$

**Figure 2:**
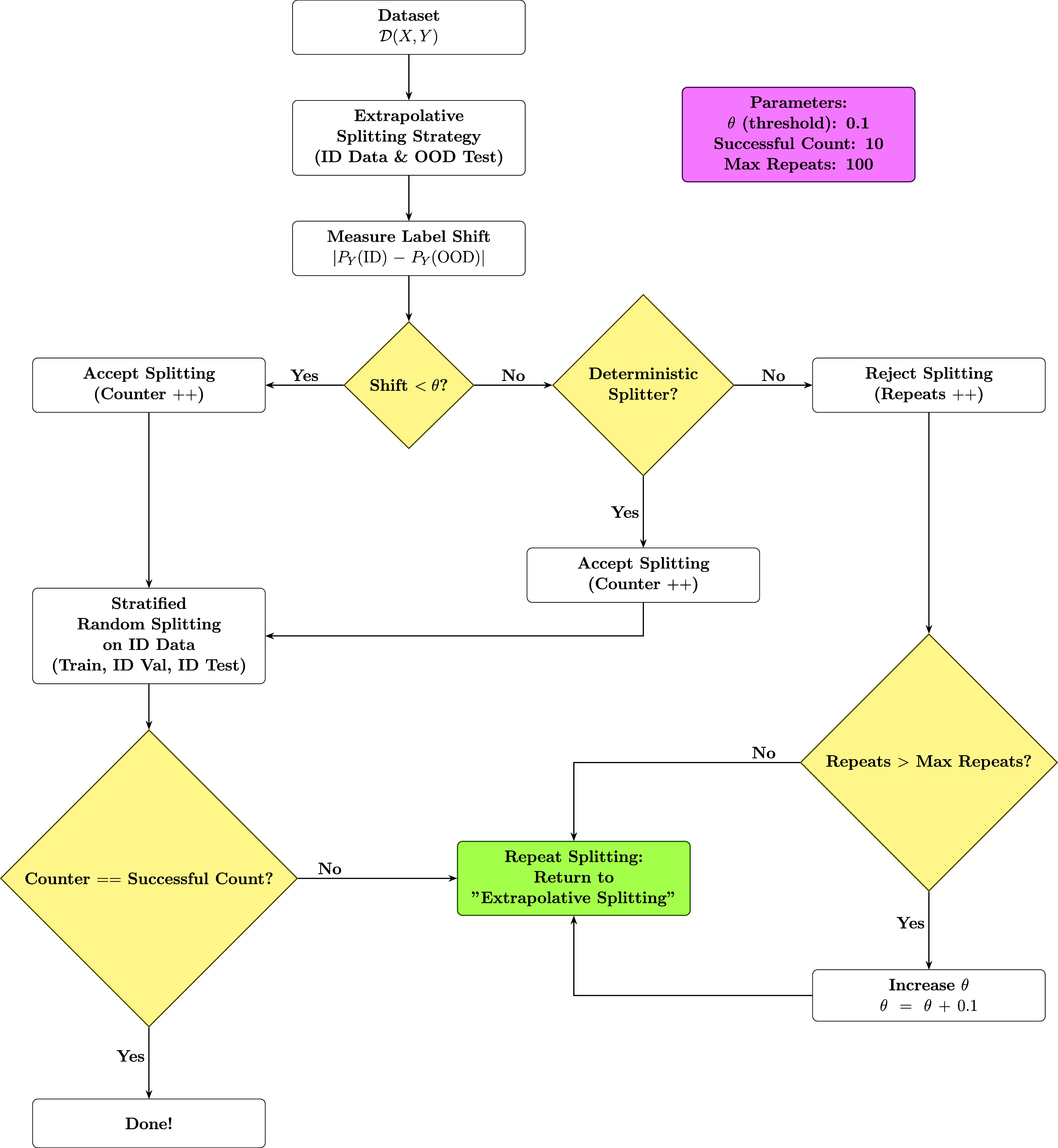
Overview of the data splitting workflow employed for creating out-of-distribution (OOD) data.

Following the initial ID-OOD splitting, the ID data set is further subdivided into a training set (72%), an ID validation set (8%), and an ID test set (20%) using stratified random splitting. The training and ID validation sets are used for model training and selection using the empirical risk minimization (ERM) algorithm. This workflow ensures consistent model evaluation, as the same training data is used when assessing performance on both ID and OOD test sets. Detailed procedures for model training and selection are provided in the Supporting Information. Moreover, the sizes of ID and OOD test sets, as well as the activity ratio of training and OOD test sets, after applying this workflow for all splitting strategies, can be found in Figures S4 and S5.

### Quantifying Distribution Shifts Between Training and Test Sets

To assess the difficulty of extrapolation and model generalization, we quantify the distance between the OOD test set and training set using two complementary molecular representations: fingerprints and graphs.

Following Sheridan’s methodology,^51^ we implement a k-nearest neighbors approach (*k* = 5) to calculate set-wise distances. For each molecule in the OOD test set, we identify its five nearest neighbors in the training set using two different representations and distance metrics: 1. Fingerprint-based distance: We generate Morgan fingerprints (radius = 2, 2048 bits) for all molecules and compute the Tanimoto similarity between pairs. 2. Graph-based distance: We represent molecules as attributed graphs, where atoms form nodes with associated features (with features in Table S3) and bonds form edges. The Tree Wasserstein distance, more specifically the Tree Mover Distance (TMD),^52^ is then used to compute the distance between these molecular graphs, accounting for topological and chemical feature differences.

The average distance to the k-nearest neighbors (*k* = 5) serves as a metric for the distribution shift between the training and test sets, with larger distances indicating more challenging extrapolation tasks. We provide box plots to show the distribution of similarities or distances between the test set molecules and their five closest training set neighbors in the Results and Discussion section.

## Results and Discussion

We report our findings organized into three key areas: (1) the analysis of the distribution shift and distance between the OOD test sets and training sets using both model-agnostic and model-dependent approaches, (2) the evaluation of the performance gap between ID and OOD test sets across different splitting methods and model architecture types, and (3) the investigation of the correlation between ID and OOD performance. Finally, we discuss limitations and future work.

### Evaluation of the Hardness of Splitting Strategies

Distribution shift, which represents how far the OOD test set deviates from the training set, can be evaluated using two complementary approaches: model-agnostic and model-dependent methods. In model-agnostic approaches, the distance between the OOD test and training sets is quantified without training ML models (as detailed in section Materials and Methods). The greater the distance, the harder the splitting strategy is considered to be. This relationship holds because larger distances indicate that the test set contains examples that are more dissimilar from the training data, making it inherently more difficult for any model to generalize successfully. In model-dependent approaches, a ML model is first trained on the training set and then evaluated on both the ID and OOD test sets. The difference in performance between these evaluations indicates the difficulty of the splitting approach used in creating OOD data. The larger the difference, the more challenging the splitter for each model.

#### Model-Agnostic Approach

For the model-agnostic approach, we calculated the distance between the OOD test set and training set using a *k*-nearest neighbors approach (*k* = 5). For both the Tanimoto and TMD metrics, all pairwise comparisons between splitting strategies showed significant differences (Tukey HSD^53^ test, *p* < 0.05), indicating that each strategy produced distinctly different distribution shifts (Figure 3).

**Figure 3:**
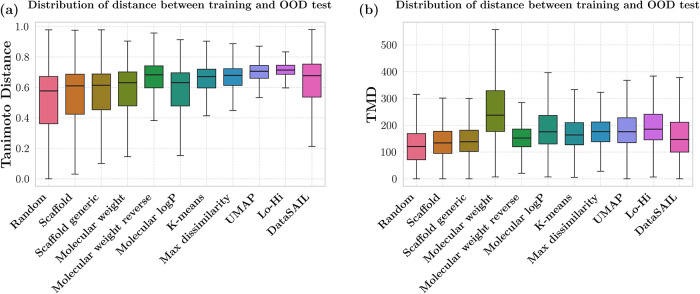
Distribution of distances (*y*-axis) between the OOD test sets and training sets for different splitters (*x*-axis). For each molecule in the OOD test set, the five nearest neighbors in the training set were identified using two distance metrics: (a) Tanimoto distance with Morgan fingerprint representation (radius 2; 2048 bits) and (b) TMD with graph representation. The results are aggregated over all test samples in all data sets.

Ranking the splitting strategies based on median values for the Tanimoto distance and TMD and comparing the ranks reveals a moderate correlation, with Spearman’s correlation coefficient ρ = 0.618 and Kendall’s Tau coefficient τ = 0.491. Both metrics identified the random, scaffold, and scaffold generic splitters (see Table 5 for definitions) as the least challenging ones under investigation (median Tanimoto distances of 0.577, 0.610, and 0.614; median TMDs of 120, 133, and 138, respectively). Prior studies also reported that scaffold splitting can lead to overly optimistic performance estimates.^24^ We hypothesize that generating scaffolds with the Bemis-Murcko algorithm results in scaffolds that represent relatively few molecules each. As the ratio of unique scaffolds to the total number of molecules increases and approaches unity (i.e., each molecule has a unique scaffold), scaffold splitting converges toward random splitting (in the extreme case, scaffold splitting converges toward random splitting, since molecules cannot be grouped by shared structural frameworks). To validate this hypothesis, we quantified the ratio of unique scaffolds to the total number of molecules for each data set (Figure S6). On average, there are only 1.93 molecules per scaffold, which increases to just 2.75 molecules per scaffold when considering generic scaffolds (with a maximum of 4.06 molecules per scaffold for the AMES data set). This low scaffold-to-molecule ratio means that most scaffolds contain very few molecules, increasing the probability that structurally similar molecules (e.g., with high Tanimoto similarity) are assigned to different scaffolds and subsequently placed in different splits (training vs test sets). This fragmentation explains why scaffold splitting fails to create challenging distribution shifts, leading to overly optimistic performance estimates.

The Tanimoto distance and TMD disagree on identifying the most challenging splitters. According to the Tanimoto distance, the most challenging splitters are Lo-Hi and UMAP clustering (medians of 0.714 and 0.705, respectively). In contrast, according to the TMD, the hardest splitters are molecular weight and Lo-Hi (medians of 237 and 185, respectively). These findings indicate that, although there is a moderate correlation between the rankings based on these two metrics, they capture different aspects of splitting strategies. It also showcases that the metrics can be used in a complementary manner.

In order to get a better intuition of how each splitting strategies split the data and distribute the molecules into ID data set and OOD test set, we also provide an example t-SNE plot (based on ECFP4, *r* = 2, and 2048 bits) for CYP2C19 as the representative data set (Figure 4).

**Figure 4:**
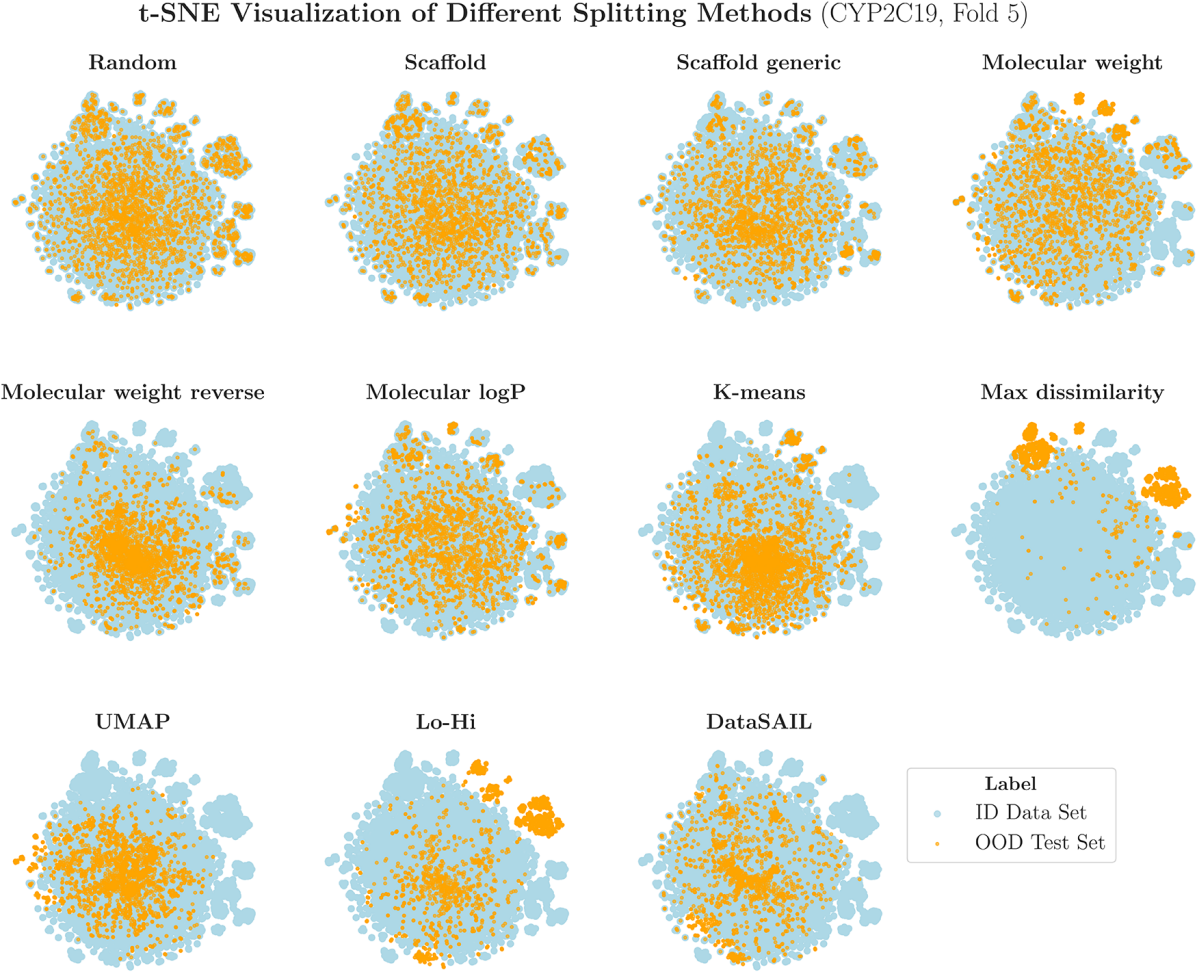
t-SNE visualization of one fold of the CYP2C19 data set across different splitting strategies. Molecular structures are represented using ECFP4 fingerprints (radius = 2, 2048 bits) and projected into 2D space. Different colors indicate ID and OOD test set assignments for each splitting method.

#### Model-Dependent Approach

In the model-dependent approach, the difference between ID and OOD test sets performance (Δ ROC-AUC) is used as the proxy for quantifying the hardness of a split. We report the Δ ROC-AUC values for both the aggregated results across all data sets and for the individual data sets (Figure 5).

**Figure 5:**
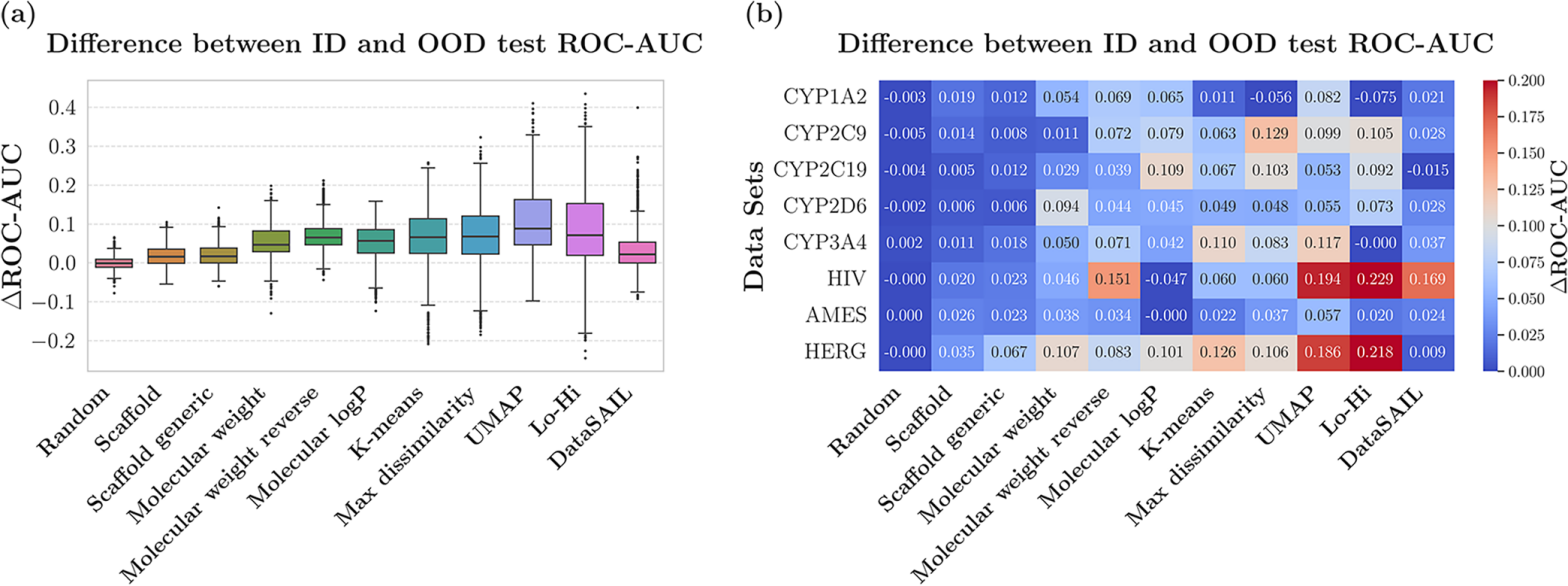
Performance gap (measured as Δ ROC-AUC) between the ID and OOD test sets with different splitters. (a) Results aggregated across all data sets and models (i.e., for each data set, Δ ROC-AUC was calculated (*y*-axis), and then all results were concatenated for each splitter (*x*-axis) (b) Mean Δ ROC-AUC between the ID and OOD sets, shown separately for each data set and splitter.

Tukey’s HSD test revealed no significant differences between several splitting strategy pairs: scaffold and scaffold-generic (*p*_adj_ = 0.40), molecular weight and molecular LogP (*p*_adj_ = 0.92), and K-means and max dissimilarity (*p*_adj_ = 0.99). The largest Δ ROC-AUC belongs to UMAP clustering following by Lo-Hi (Median 0.088 and 0.070, respectively). Since this approach is model-dependent, the results (including the ranking of the splitters) can differ depending on the ML algorithm employed.

We investigated and computed the correlation between rankings derived from the model-agnostic and model-dependent approaches (Table 6). Within the model-dependent approach, we found that there is a strong correlation between classical ML and GNN-based methods rankings (Spearman’s ρ = 0.936), suggesting a consistent splitter ranking across these architectures. When comparing the correlations between the splitter rankings by model-agnostic and model-dependent approaches, we found that the Tanimoto metric rankings showed stronger correlations to both classical ML and GNN-based methods rankings than the TMD rankings (Tanimoto distances: Spearman’s ρ = 0.909 and 0.900; TMDs: Spearman’s ρ = 0.673 and 0.736, respectively).

**Table 6:** Pairwise Rank Correlation between Splitter Hardness Rankings across Different Approaches, Measured Using Spearman’s Correlation (*ρ*) and Kendall’s Tau (*τ*)

Method 1	Method 2	Spearman’s ρ	Kendall’s τ
Model-Agnostic (Tanimoto)	Model-Agnostic (TMD)	0.618	0.491
Model-Agnostic (Tanimoto)	Model-Dependent (Classical ML)	0.909	0.782
Model-Agnostic (Tanimoto)	Model-Dependent (GNN-based methods)	0.900	0.782
Model-Agnostic (TMD)	Model-Dependent (Classical ML)	0.673	0.491
Model-Agnostic (TMD)	Model-Dependent (GNN-based methods)	0.736	0.564
Model-Dependent (Classical ML)	Model-Dependent (GNN-based methods)	0.936	0.855

Strong correlation of Tanimoto metric rankings to both classical ML and GNN-based methods rankings has significant practical implications: model-agnostic Tanimoto distance can reliably predict the relative difficulty of different splitting strategies without the computational expense of training multiple models. Given that both Tanimoto distance and classical ML models use the Morgan fingerprint, a strong correlation was expected between their rankings. However, the similarly strong correlation between Tanimoto distance and GNN-based models was unexpected. These observations indicate that the Tanimoto distance can serve as an efficient and reliable predictor of performance drops across different splitting methods, for both classical ML and GNN-based models. Thus, the Tanimoto distance offers a computationally efficient approach for assessing splitting strategy difficulty in experimental design.

### Comparing the Performance of Classical ML, GNNs, and Pretrained GNNs on OOD Data

We conducted a comprehensive analysis of the ID performance, OOD performance, and the performance gap (difference between ID and OOD ROC-AUC values) across all the splitters, data sets, and models. Our primary objective was to investigate whether classical ML, GNN, and pretrained GNN models behave differently on OOD data or if they follow a similar pattern. This comparison is important for understanding if the concept of OOD varies between these three categories of models.

We first present the results aggregated and averaged across all the models (Table 7). This is followed by the comparative analysis of classical ML, GNNs, and pretrained GNN models (Table 8, Figure 6, and Figure 7).

**Figure 6:**
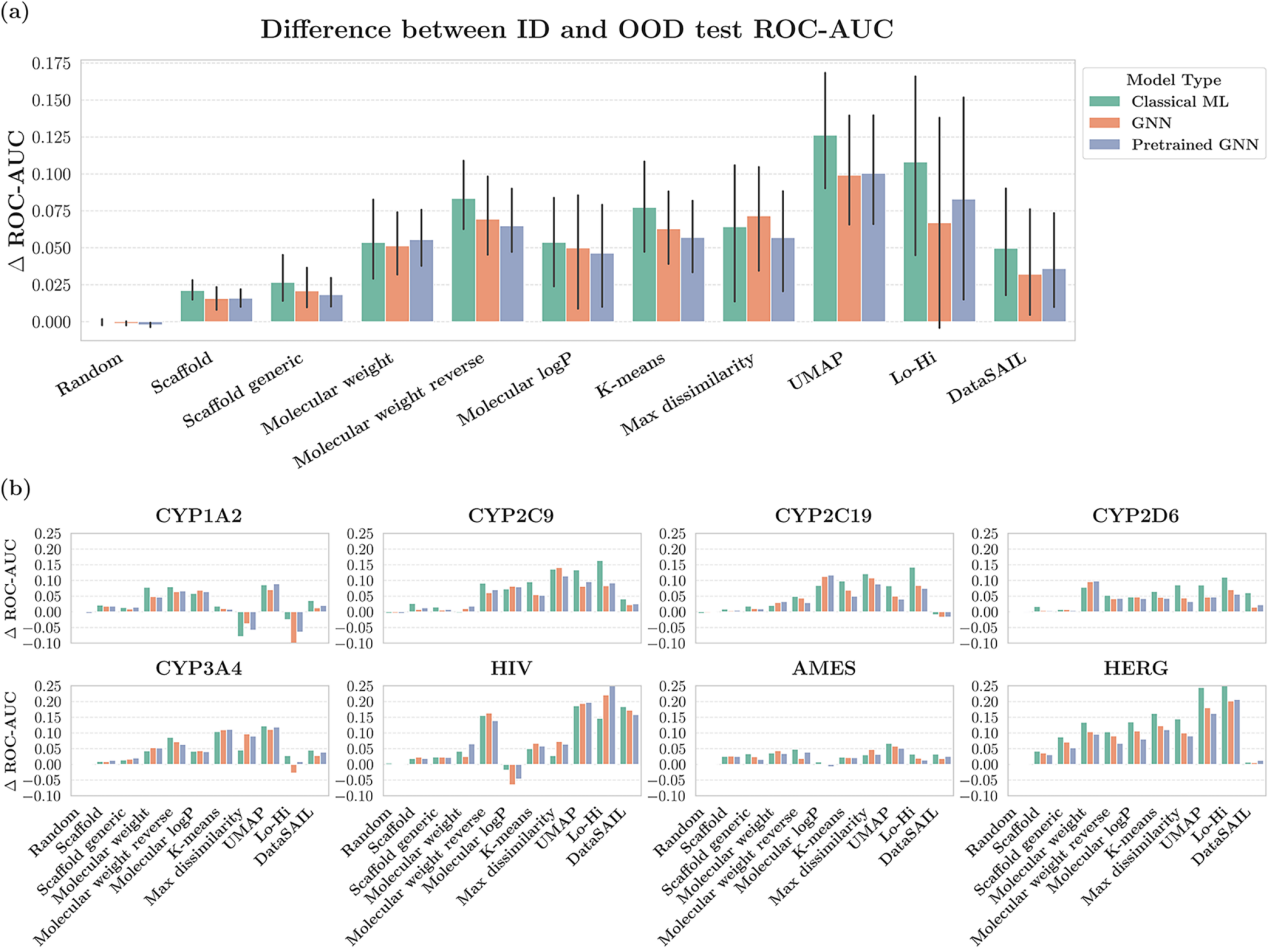
Performance gap (measured as Δ ROC-AUC), between ID and OOD test sets for classical ML and GNN models. The figure presents both the aggregated results across all data sets and the results for each data set separately.

**Figure 7:**
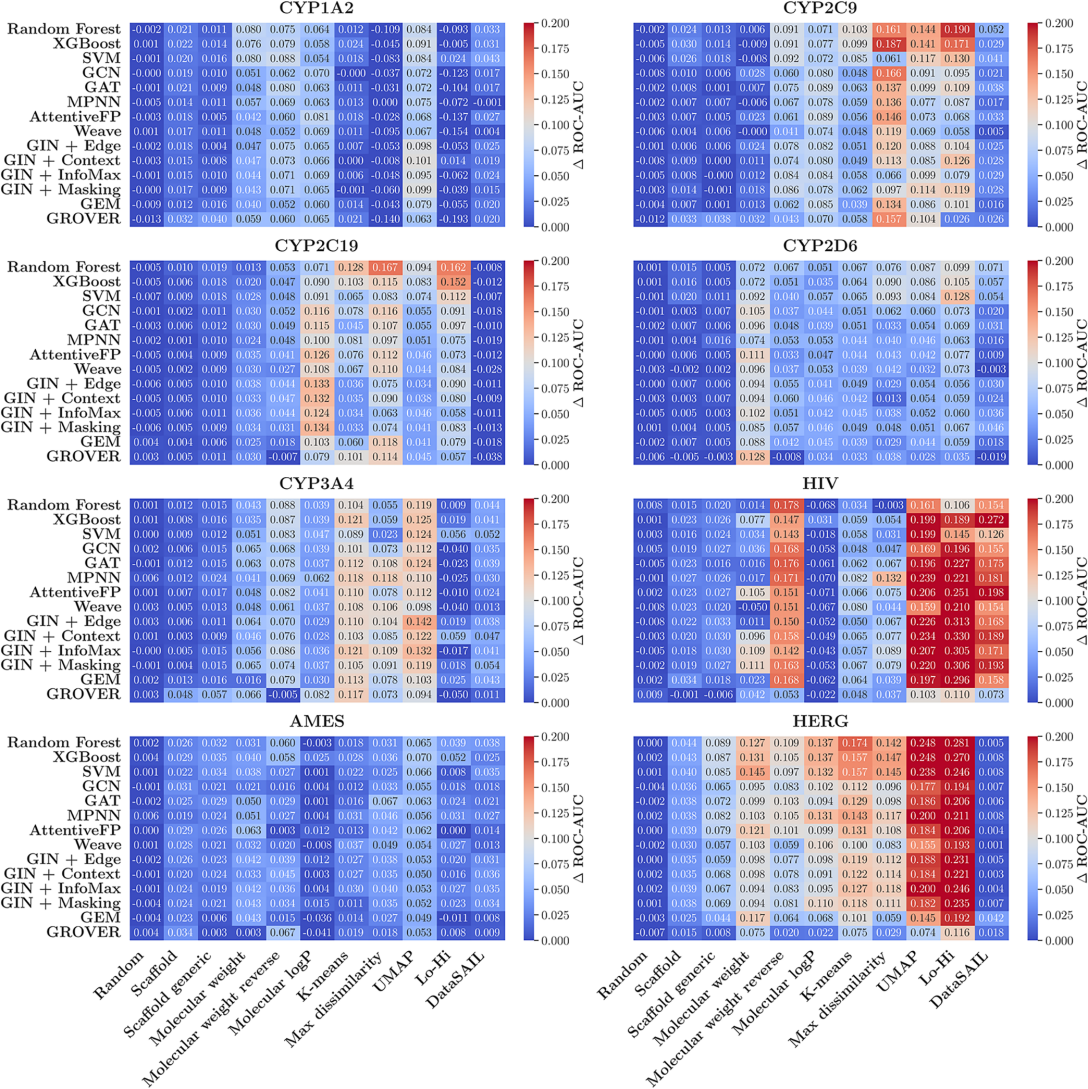
Performance gap (measured as Δ ROC-AUC), between ID and OOD test sets across different splitters (*x*-axis), models (*y*-axis), and data sets.

**Table 7:** Performance Gap in Terms of ROC-AUC between the In-Distribution (ID) and Out-of-Distribution (OOD) Test Sets^a^ ^,^ ^b^

		data sets							
split type	metric	CYP1A2	CYP2C9	CYP2C19	CYP2D6	CYP3A4	HIV	AMES	HERG
Random	Test (ID)	0.77 (0.01)	0.78 (0.02)	0.85 (0.01)	0.85 (0.04)	0.76 (0.02)	0.78 (0.07)	0.67 (0.02)	0.87 (0.04)
	Test (OOD)	0.78 (0.01)	0.78 (0.02)	0.85 (0.01)	0.85 (0.03)	0.76 (0.02)	0.78 (0.07)	0.67 (0.02)	0.87 (0.03)
	Gap	–0.00 (0.02)	–0.00 (0.01)	–0.00 (0.01)	–0.00 (0.01)	0.00 (0.01)	–0.00 (0.03)	0.00 (0.02)	–0.00 (0.01)
Scaffold	Test (ID)	0.78 (0.01)	0.78 (0.02)	0.85 (0.01)	0.85 (0.02)	0.75 (0.02)	0.77 (0.06)	0.67 (0.02)	0.87 (0.04)
	Test (OOD)	0.76 (0.02)	0.77 (0.02)	0.85 (0.02)	0.85 (0.02)	0.74 (0.04)	0.75 (0.06)	0.64 (0.02)	0.84 (0.03)
	Gap	0.02 (0.02)	0.01 (0.02)	0.01 (0.02)	0.01 (0.02)	0.01 (0.03)	0.02 (0.04)	0.03 (0.02)	0.04 (0.02)
Scaffold generic	Test (ID)	0.77 (0.01)	0.78 (0.02)	0.85 (0.01)	0.85 (0.02)	0.76 (0.02)	0.78 (0.06)	0.66 (0.03)	0.88 (0.06)
	Test (OOD)	0.76 (0.02)	0.77 (0.02)	0.84 (0.01)	0.85 (0.02)	0.74 (0.03)	0.76 (0.06)	0.64 (0.03)	0.81 (0.04)
	Gap	0.01 (0.02)	0.01 (0.01)	0.01 (0.01)	0.01 (0.02)	0.02 (0.03)	0.02 (0.04)	0.02 (0.02)	0.07 (0.03)
Molecular weight	Test (ID)	0.78 (0.01)	0.76 (0.01)	0.86 (0.01)	0.86 (0.01)	0.75 (0.02)	0.72 (0.04)	0.66 (0.01)	0.88 (0.03)
	Test (OOD)	0.72 (0.02)	0.75 (0.02)	0.83 (0.01)	0.77 (0.02)	0.70 (0.03)	0.67 (0.06)	0.62 (0.02)	0.77 (0.02)
	Gap	0.05 (0.02)	0.01 (0.02)	0.03 (0.01)	0.09 (0.02)	0.05 (0.02)	0.05 (0.06)	0.04 (0.02)	0.11 (0.02)
Molecular weight reverse	Test (ID)	0.78 (0.01)	0.78 (0.02)	0.85 (0.02)	0.87 (0.02)	0.74 (0.04)	0.78 (0.06)	0.67 (0.02)	0.88 (0.04)
	Test (OOD)	0.71 (0.01)	0.71 (0.02)	0.81 (0.02)	0.82 (0.01)	0.67 (0.02)	0.63 (0.04)	0.64 (0.03)	0.79 (0.02)
	Gap	0.07 (0.01)	0.07 (0.02)	0.04 (0.02)	0.04 (0.02)	0.07 (0.02)	0.15 (0.03)	0.03 (0.02)	0.08 (0.03)
Molecular logp	Test (ID)	0.79 (0.01)	0.79 (0.02)	0.87 (0.02)	0.85 (0.02)	0.75 (0.02)	0.77 (0.07)	0.67 (0.02)	0.88 (0.04)
	Test (OOD)	0.72 (0.01)	0.71 (0.02)	0.76 (0.02)	0.80 (0.02)	0.70 (0.03)	0.82 (0.08)	0.67 (0.03)	0.78 (0.02)
	Gap	0.07 (0.01)	0.08 (0.01)	0.11 (0.02)	0.05 (0.01)	0.04 (0.02)	–0.05 (0.03)	–0.00 (0.02)	0.10 (0.03)
K-means	Test (ID)	0.77 (0.03)	0.79 (0.02)	0.85 (0.01)	0.86 (0.02)	0.77 (0.03)	0.78 (0.07)	0.66 (0.02)	0.88 (0.03)
	Test (OOD)	0.76 (0.10)	0.73 (0.04)	0.78 (0.06)	0.81 (0.03)	0.66 (0.04)	0.72 (0.09)	0.64 (0.03)	0.75 (0.04)
	Gap	0.01 (0.13)	0.06 (0.05)	0.07 (0.05)	0.05 (0.03)	0.11 (0.04)	0.06 (0.09)	0.02 (0.03)	0.13 (0.04)
Max dissimilarity	Test (ID)	0.74 (0.01)	0.77 (0.01)	0.84 (0.01)	0.85 (0.02)	0.74 (0.02)	0.78 (0.07)	0.67 (0.02)	0.87 (0.04)
	Test (OOD)	0.80 (0.08)	0.64 (0.06)	0.74 (0.07)	0.80 (0.04)	0.65 (0.07)	0.72 (0.10)	0.64 (0.02)	0.77 (0.05)
	Gap	–0.06 (0.09)	0.13 (0.06)	0.10 (0.07)	0.05 (0.04)	0.08 (0.08)	0.06 (0.07)	0.04 (0.02)	0.11 (0.06)
UMAP	Test (ID)	0.77 (0.03)	0.78 (0.02)	0.85 (0.01)	0.86 (0.02)	0.77 (0.02)	0.79 (0.07)	0.67 (0.02)	0.88 (0.04)
	Test (OOD)	0.69 (0.08)	0.68 (0.05)	0.80 (0.04)	0.80 (0.03)	0.65 (0.04)	0.60 (0.09)	0.62 (0.03)	0.70 (0.04)
	Gap	0.08 (0.11)	0.10 (0.05)	0.05 (0.04)	0.06 (0.03)	0.12 (0.05)	0.19 (0.09)	0.06 (0.03)	0.19 (0.06)
Lo-Hi	Test (ID)	0.70 (0.01)	0.79 (0.02)	0.85 (0.01)	0.87 (0.02)	0.72 (0.03)	0.78 (0.06)	0.65 (0.02)	0.88 (0.03)
	Test (OOD)	0.77 (0.09)	0.68 (0.04)	0.76 (0.03)	0.79 (0.03)	0.72 (0.04)	0.55 (0.09)	0.63 (0.02)	0.66 (0.03)
	Gap	–0.08 (0.09)	0.10 (0.05)	0.09 (0.03)	0.07 (0.02)	–0.00 (0.04)	0.23 (0.09)	0.02 (0.02)	0.22 (0.04)
DataSAIL	Test (ID)	0.76 (0.01)	0.79 (0.02)	0.85 (0.01)	0.85 (0.02)	0.75 (0.02)	0.79 (0.06)	0.68 (0.02)	0.87 (0.04)
	Test (OOD)	0.74 (0.04)	0.76 (0.04)	0.86 (0.02)	0.83 (0.03)	0.72 (0.04)	0.62 (0.05)	0.65 (0.02)	0.87 (0.05)
	Gap	0.02 (0.05)	0.03 (0.03)	–0.02 (0.02)	0.03 (0.03)	0.04 (0.04)	0.17 (0.05)	0.02 (0.01)	0.01 (0.04)

a Performance is averaged over replications and models that are included in classical ML and GNN-based categories.

b Parentheses show the standard deviations across models and replicates.

**Table 8:** Performance Gap in Terms of ROC-AUC between In-Distribution (ID) and Out-of-Distribution (OOD) Test Set

			data sets							
split type	model type	metric	CYP1A2	CYP2C9	CYP2C19	CYP2D6	CYP3A4	HIV	AMES	HERG
Random	Classical ML	Test (ID)	0.77 (0.01)	0.79 (0.02)	0.85 (0.01)	0.86 (0.01)	0.77 (0.01)	0.81 (0.02)	0.66 (0.02)	0.90 (0.01)
		Test (OOD)	0.77 (0.02)	0.79 (0.01)	0.85 (0.01)	0.86 (0.01)	0.77 (0.01)	0.81 (0.02)	0.66 (0.02)	0.90 (0.01)
		Gap	–0.00 (0.02)	–0.00 (0.01)	–0.01 (0.01)	0.00 (0.01)	0.00 (0.01)	0.00 (0.02)	0.00 (0.02)	0.00 (0.01)
	GNN	Test (ID)	0.77 (0.01)	0.78 (0.02)	0.85 (0.01)	0.85 (0.01)	0.76 (0.02)	0.79 (0.02)	0.67 (0.02)	0.87 (0.02)
		Test (OOD)	0.78 (0.01)	0.78 (0.01)	0.86 (0.01)	0.85 (0.01)	0.75 (0.02)	0.79 (0.03)	0.67 (0.01)	0.87 (0.02)
		Gap	–0.00 (0.01)	–0.00 (0.01)	–0.00 (0.01)	–0.00 (0.01)	0.00 (0.01)	–0.00 (0.03)	0.00 (0.02)	–0.00 (0.01)
	Pretrained GNN	Test (ID)	0.77 (0.01)	0.78 (0.02)	0.85 (0.02)	0.84 (0.05)	0.75 (0.03)	0.75 (0.10)	0.66 (0.03)	0.85 (0.04)
		Test (OOD)	0.78 (0.01)	0.78 (0.02)	0.85 (0.02)	0.85 (0.05)	0.75 (0.03)	0.75 (0.10)	0.66 (0.03)	0.85 (0.04)
		Gap	–0.00 (0.01)	–0.01 (0.01)	–0.00 (0.01)	–0.00 (0.01)	0.00 (0.01)	–0.00 (0.03)	–0.00 (0.02)	–0.00 (0.01)
Scaffold	Classical ML	Test (ID)	0.77 (0.01)	0.79 (0.01)	0.85 (0.00)	0.85 (0.01)	0.77 (0.01)	0.80 (0.02)	0.66 (0.02)	0.90 (0.01)
		Test (OOD)	0.75 (0.02)	0.77 (0.02)	0.84 (0.01)	0.84 (0.01)	0.76 (0.02)	0.78 (0.02)	0.63 (0.03)	0.86 (0.01)
		Gap	0.02 (0.02)	0.03 (0.02)	0.01 (0.01)	0.02 (0.02)	0.01 (0.02)	0.02 (0.04)	0.03 (0.02)	0.04 (0.01)
	GNN	Test (ID)	0.78 (0.01)	0.78 (0.01)	0.85 (0.01)	0.85 (0.02)	0.75 (0.02)	0.77 (0.03)	0.67 (0.02)	0.88 (0.02)
		Test (OOD)	0.76 (0.02)	0.77 (0.01)	0.85 (0.01)	0.84 (0.02)	0.74 (0.02)	0.75 (0.03)	0.64 (0.02)	0.84 (0.02)
		Gap	0.02 (0.02)	0.01 (0.01)	0.00 (0.02)	0.00 (0.02)	0.01 (0.03)	0.02 (0.04)	0.03 (0.02)	0.04 (0.01)
	Pretrained GNN	Test (ID)	0.78 (0.01)	0.78 (0.02)	0.85 (0.01)	0.85 (0.02)	0.75 (0.03)	0.76 (0.09)	0.66 (0.02)	0.86 (0.05)
		Test (OOD)	0.76 (0.02)	0.77 (0.03)	0.85 (0.02)	0.85 (0.02)	0.74 (0.05)	0.74 (0.08)	0.64 (0.03)	0.82 (0.04)
		Gap	0.02 (0.02)	0.01 (0.02)	0.00 (0.02)	0.00 (0.02)	0.01 (0.03)	0.02 (0.04)	0.03 (0.02)	0.03 (0.02)
Scaffold generic	Classical ML	Test (ID)	0.77 (0.01)	0.79 (0.01)	0.85 (0.01)	0.85 (0.01)	0.77 (0.01)	0.81 (0.02)	0.65 (0.02)	0.91 (0.01)
		Test (OOD)	0.76 (0.02)	0.77 (0.01)	0.83 (0.01)	0.85 (0.01)	0.76 (0.02)	0.78 (0.03)	0.62 (0.02)	0.82 (0.02)
		Gap	0.01 (0.02)	0.02 (0.01)	0.02 (0.01)	0.01 (0.02)	0.01 (0.02)	0.02 (0.04)	0.03 (0.02)	0.09 (0.02)
	GNN	Test (ID)	0.77 (0.01)	0.77 (0.01)	0.85 (0.01)	0.85 (0.01)	0.76 (0.02)	0.78 (0.03)	0.67 (0.02)	0.89 (0.02)
		Test (OOD)	0.76 (0.02)	0.77 (0.01)	0.84 (0.01)	0.84 (0.02)	0.74 (0.02)	0.76 (0.04)	0.65 (0.02)	0.82 (0.02)
		Gap	0.01 (0.02)	0.01 (0.01)	0.01 (0.01)	0.01 (0.02)	0.02 (0.03)	0.02 (0.04)	0.02 (0.02)	0.07 (0.02)
	Pretrained GNN	Test (ID)	0.77 (0.01)	0.78 (0.02)	0.85 (0.02)	0.85 (0.03)	0.76 (0.03)	0.77 (0.09)	0.66 (0.04)	0.85 (0.08)
		Test (OOD)	0.76 (0.02)	0.77 (0.03)	0.84 (0.02)	0.85 (0.03)	0.74 (0.04)	0.74 (0.08)	0.64 (0.03)	0.80 (0.06)
		Gap	0.01 (0.03)	0.01 (0.02)	0.01 (0.01)	0.00 (0.02)	0.02 (0.03)	0.02 (0.04)	0.02 (0.02)	0.05 (0.03)
Molecular weight	Classical ML	Test (ID)	0.78 (0.01)	0.76 (0.01)	0.85 (0.00)	0.86 (0.00)	0.76 (0.01)	0.74 (0.01)	0.65 (0.01)	0.90 (0.01)
		Test (OOD)	0.70 (0.01)	0.76 (0.00)	0.83 (0.01)	0.78 (0.01)	0.72 (0.01)	0.70 (0.03)	0.62 (0.01)	0.77 (0.00)
		Gap	0.08 (0.00)	–0.00 (0.01)	0.02 (0.01)	0.08 (0.01)	0.04 (0.01)	0.04 (0.03)	0.04 (0.00)	0.13 (0.01)
	GNN	Test (ID)	0.78 (0.01)	0.76 (0.01)	0.87 (0.01)	0.86 (0.01)	0.76 (0.01)	0.70 (0.03)	0.66 (0.01)	0.88 (0.02)
		Test (OOD)	0.73 (0.01)	0.75 (0.01)	0.84 (0.01)	0.77 (0.01)	0.70 (0.02)	0.68 (0.06)	0.62 (0.02)	0.77 (0.02)
		Gap	0.05 (0.01)	0.01 (0.02)	0.03 (0.01)	0.10 (0.02)	0.05 (0.02)	0.02 (0.07)	0.04 (0.02)	0.10 (0.01)
	Pretrained GNN	Test (ID)	0.78 (0.01)	0.76 (0.02)	0.86 (0.02)	0.86 (0.02)	0.75 (0.03)	0.72 (0.06)	0.66 (0.02)	0.86 (0.04)
		Test (OOD)	0.73 (0.02)	0.75 (0.02)	0.83 (0.02)	0.77 (0.03)	0.70 (0.04)	0.66 (0.07)	0.62 (0.02)	0.77 (0.03)
		Gap	0.05 (0.01)	0.02 (0.01)	0.03 (0.01)	0.10 (0.02)	0.05 (0.02)	0.07 (0.06)	0.03 (0.02)	0.10 (0.02)
Molecular weight reverse	Classical ML	Test (ID)	0.79 (0.01)	0.79 (0.01)	0.85 (0.00)	0.87 (0.01)	0.76 (0.01)	0.80 (0.00)	0.68 (0.02)	0.90 (0.00)
		Test (OOD)	0.71 (0.01)	0.70 (0.01)	0.80 (0.00)	0.82 (0.01)	0.68 (0.01)	0.65 (0.02)	0.63 (0.01)	0.80 (0.01)
		Gap	0.08 (0.01)	0.09 (0.00)	0.05 (0.00)	0.05 (0.01)	0.09 (0.00)	0.16 (0.02)	0.05 (0.02)	0.10 (0.01)
	GNN	Test (ID)	0.78 (0.01)	0.78 (0.01)	0.86 (0.01)	0.87 (0.01)	0.75 (0.02)	0.79 (0.01)	0.66 (0.01)	0.88 (0.02)
		Test (OOD)	0.72 (0.01)	0.71 (0.02)	0.81 (0.01)	0.83 (0.01)	0.68 (0.02)	0.62 (0.02)	0.65 (0.02)	0.79 (0.02)
		Gap	0.06 (0.01)	0.06 (0.02)	0.04 (0.01)	0.04 (0.01)	0.07 (0.01)	0.16 (0.02)	0.02 (0.02)	0.09 (0.02)
	Pretrained GNN	Test (ID)	0.78 (0.01)	0.78 (0.03)	0.85 (0.03)	0.87 (0.03)	0.73 (0.05)	0.76 (0.08)	0.67 (0.02)	0.86 (0.05)
		Test (OOD)	0.72 (0.01)	0.71 (0.02)	0.82 (0.02)	0.82 (0.01)	0.67 (0.03)	0.63 (0.05)	0.63 (0.04)	0.79 (0.03)
		Gap	0.07 (0.01)	0.07 (0.02)	0.03 (0.02)	0.04 (0.03)	0.06 (0.03)	0.14 (0.04)	0.04 (0.02)	0.07 (0.03)
Molecular logp	Classical ML	Test (ID)	0.78 (0.01)	0.80 (0.01)	0.86 (0.00)	0.85 (0.00)	0.76 (0.01)	0.81 (0.01)	0.66 (0.01)	0.91 (0.01)
		Test (OOD)	0.72 (0.01)	0.72 (0.01)	0.78 (0.01)	0.80 (0.01)	0.71 (0.01)	0.83 (0.03)	0.65 (0.02)	0.77 (0.01)
		Gap	0.06 (0.00)	0.07 (0.00)	0.08 (0.01)	0.05 (0.01)	0.04 (0.00)	–0.02 (0.04)	0.01 (0.01)	0.14 (0.00)
	GNN	Test (ID)	0.79 (0.01)	0.78 (0.01)	0.87 (0.01)	0.85 (0.01)	0.75 (0.01)	0.76 (0.03)	0.68 (0.01)	0.89 (0.02)
		Test (OOD)	0.72 (0.01)	0.70 (0.01)	0.76 (0.01)	0.80 (0.02)	0.71 (0.02)	0.83 (0.03)	0.68 (0.01)	0.78 (0.03)
		Gap	0.07 (0.01)	0.08 (0.01)	0.11 (0.01)	0.05 (0.01)	0.04 (0.01)	–0.07 (0.02)	0.00 (0.01)	0.11 (0.02)
	Pretrained GNN	Test (ID)	0.79 (0.01)	0.79 (0.02)	0.87 (0.02)	0.85 (0.02)	0.74 (0.03)	0.75 (0.10)	0.66 (0.03)	0.87 (0.04)
		Test (OOD)	0.72 (0.01)	0.71 (0.02)	0.75 (0.01)	0.80 (0.02)	0.70 (0.05)	0.80 (0.12)	0.67 (0.04)	0.78 (0.02)
		Gap	0.06 (0.01)	0.08 (0.01)	0.12 (0.02)	0.04 (0.01)	0.04 (0.02)	–0.05 (0.03)	–0.01 (0.03)	0.08 (0.03)
K-means	Classical ML	Test (ID)	0.77 (0.03)	0.80 (0.01)	0.85 (0.01)	0.86 (0.01)	0.78 (0.02)	0.81 (0.02)	0.65 (0.02)	0.91 (0.01)
		Test (OOD)	0.75 (0.11)	0.70 (0.04)	0.75 (0.07)	0.80 (0.02)	0.67 (0.04)	0.76 (0.07)	0.63 (0.04)	0.74 (0.04)
		Gap	0.02 (0.14)	0.10 (0.04)	0.10 (0.07)	0.07 (0.02)	0.10 (0.05)	0.05 (0.08)	0.02 (0.04)	0.16 (0.05)
	GNN	Test (ID)	0.77 (0.03)	0.78 (0.02)	0.85 (0.01)	0.86 (0.01)	0.77 (0.02)	0.78 (0.04)	0.66 (0.01)	0.88 (0.02)
		Test (OOD)	0.76 (0.10)	0.73 (0.04)	0.78 (0.05)	0.81 (0.03)	0.66 (0.03)	0.71 (0.08)	0.64 (0.03)	0.76 (0.04)
		Gap	0.01 (0.12)	0.05 (0.05)	0.07 (0.04)	0.05 (0.03)	0.11 (0.03)	0.07 (0.09)	0.02 (0.03)	0.12 (0.04)
	Pretrained GNN	Test (ID)	0.77 (0.03)	0.79 (0.02)	0.85 (0.02)	0.86 (0.02)	0.77 (0.03)	0.77 (0.10)	0.66 (0.02)	0.87 (0.04)
		Test (OOD)	0.76 (0.10)	0.74 (0.04)	0.80 (0.06)	0.82 (0.03)	0.65 (0.04)	0.71 (0.11)	0.64 (0.03)	0.76 (0.03)
		Gap	0.01 (0.13)	0.05 (0.04)	0.05 (0.05)	0.04 (0.03)	0.11 (0.03)	0.06 (0.10)	0.02 (0.03)	0.11 (0.03)
Max dissimilarity	Classical ML	Test (ID)	0.73 (0.02)	0.76 (0.01)	0.83 (0.01)	0.85 (0.01)	0.74 (0.01)	0.81 (0.02)	0.67 (0.02)	0.90 (0.01)
		Test (OOD)	0.81 (0.07)	0.63 (0.07)	0.71 (0.07)	0.76 (0.04)	0.69 (0.06)	0.78 (0.05)	0.64 (0.02)	0.76 (0.06)
		Gap	–0.08 (0.07)	0.14 (0.07)	0.12 (0.07)	0.09 (0.04)	0.05 (0.07)	0.03 (0.05)	0.03 (0.02)	0.14 (0.06)
	GNN	Test (ID)	0.74 (0.01)	0.77 (0.01)	0.85 (0.01)	0.85 (0.02)	0.74 (0.02)	0.79 (0.02)	0.68 (0.02)	0.88 (0.03)
		Test (OOD)	0.78 (0.09)	0.63 (0.05)	0.74 (0.07)	0.80 (0.03)	0.64 (0.07)	0.72 (0.07)	0.63 (0.02)	0.78 (0.04)
		Gap	–0.04 (0.09)	0.14 (0.05)	0.11 (0.07)	0.04 (0.04)	0.10 (0.08)	0.07 (0.07)	0.05 (0.02)	0.10 (0.04)
	Pretrained GNN	Test (ID)	0.74 (0.01)	0.77 (0.02)	0.84 (0.02)	0.85 (0.03)	0.73 (0.03)	0.76 (0.11)	0.67 (0.03)	0.86 (0.05)
		Test (OOD)	0.80 (0.09)	0.65 (0.05)	0.75 (0.07)	0.82 (0.04)	0.64 (0.07)	0.69 (0.12)	0.64 (0.02)	0.77 (0.05)
		Gap	–0.06 (0.09)	0.11 (0.05)	0.09 (0.07)	0.03 (0.03)	0.09 (0.08)	0.06 (0.08)	0.03 (0.02)	0.09 (0.06)
UMAP	Classical ML	Test (ID)	0.77 (0.04)	0.78 (0.02)	0.86 (0.01)	0.86 (0.02)	0.78 (0.02)	0.82 (0.02)	0.67 (0.02)	0.91 (0.01)
		Test (OOD)	0.68 (0.07)	0.65 (0.04)	0.77 (0.04)	0.78 (0.03)	0.66 (0.04)	0.64 (0.04)	0.60 (0.03)	0.66 (0.04)
		Gap	0.09 (0.11)	0.13 (0.03)	0.08 (0.04)	0.09 (0.04)	0.12 (0.04)	0.19 (0.05)	0.07 (0.02)	0.24 (0.04)
	GNN	Test (ID)	0.77 (0.03)	0.78 (0.02)	0.86 (0.01)	0.85 (0.02)	0.77 (0.02)	0.80 (0.03)	0.68 (0.01)	0.88 (0.02)
		Test (OOD)	0.70 (0.08)	0.70 (0.05)	0.81 (0.04)	0.81 (0.02)	0.66 (0.04)	0.61 (0.07)	0.62 (0.02)	0.70 (0.04)
		Gap	0.07 (0.11)	0.08 (0.05)	0.05 (0.04)	0.05 (0.03)	0.11 (0.04)	0.19 (0.07)	0.06 (0.03)	0.18 (0.05)
	Pretrained GNN	Test (ID)	0.77 (0.03)	0.78 (0.03)	0.85 (0.02)	0.85 (0.03)	0.77 (0.03)	0.78 (0.09)	0.67 (0.02)	0.87 (0.04)
		Test (OOD)	0.68 (0.08)	0.68 (0.06)	0.81 (0.03)	0.81 (0.03)	0.65 (0.04)	0.58 (0.11)	0.62 (0.03)	0.70 (0.04)
		Gap	0.09 (0.11)	0.10 (0.05)	0.04 (0.03)	0.05 (0.02)	0.12 (0.05)	0.20 (0.11)	0.05 (0.03)	0.16 (0.06)
Lo-Hi	Classical ML	Test (ID)	0.68 (0.02)	0.78 (0.01)	0.85 (0.00)	0.87 (0.00)	0.73 (0.01)	0.81 (0.00)	0.66 (0.02)	0.90 (0.00)
		Test (OOD)	0.71 (0.07)	0.62 (0.01)	0.70 (0.02)	0.76 (0.01)	0.70 (0.03)	0.66 (0.04)	0.62 (0.01)	0.64 (0.02)
		Gap	–0.02 (0.05)	0.16 (0.03)	0.14 (0.02)	0.11 (0.01)	0.03 (0.02)	0.15 (0.04)	0.03 (0.02)	0.27 (0.02)
	GNN	Test (ID)	0.70 (0.01)	0.79 (0.01)	0.86 (0.01)	0.87 (0.01)	0.72 (0.02)	0.79 (0.03)	0.65 (0.02)	0.88 (0.02)
		Test (OOD)	0.82 (0.05)	0.70 (0.03)	0.77 (0.01)	0.80 (0.02)	0.75 (0.02)	0.57 (0.06)	0.63 (0.02)	0.68 (0.02)
		Gap	–0.12 (0.05)	0.08 (0.03)	0.08 (0.02)	0.07 (0.01)	–0.03 (0.02)	0.22 (0.07)	0.02 (0.02)	0.20 (0.02)
	Pretrained GNN	Test (ID)	0.70 (0.01)	0.79 (0.02)	0.85 (0.01)	0.86 (0.02)	0.72 (0.04)	0.76 (0.08)	0.65 (0.02)	0.87 (0.04)
		Test (OOD)	0.76 (0.10)	0.70 (0.03)	0.77 (0.02)	0.81 (0.02)	0.71 (0.05)	0.48 (0.05)	0.64 (0.02)	0.66 (0.02)
		Gap	–0.06 (0.10)	0.09 (0.04)	0.07 (0.02)	0.06 (0.01)	0.01 (0.05)	0.28 (0.10)	0.01 (0.02)	0.21 (0.05)
DataSAIL	Classical ML	Test (ID)	0.76 (0.01)	0.80 (0.01)	0.84 (0.01)	0.86 (0.01)	0.77 (0.01)	0.81 (0.00)	0.67 (0.01)	0.90 (0.01)
		Test (OOD)	0.72 (0.04)	0.75 (0.03)	0.85 (0.02)	0.80 (0.02)	0.72 (0.03)	0.63 (0.06)	0.64 (0.02)	0.89 (0.01)
		Gap	0.04 (0.04)	0.04 (0.03)	–0.01 (0.02)	0.06 (0.02)	0.05 (0.03)	0.18 (0.06)	0.03 (0.01)	0.01 (0.01)
	GNN	Test (ID)	0.76 (0.01)	0.78 (0.01)	0.85 (0.01)	0.85 (0.02)	0.75 (0.02)	0.80 (0.02)	0.68 (0.01)	0.88 (0.02)
		Test (OOD)	0.75 (0.04)	0.76 (0.03)	0.87 (0.01)	0.84 (0.02)	0.72 (0.04)	0.62 (0.04)	0.66 (0.01)	0.87 (0.02)
		Gap	0.01 (0.05)	0.02 (0.03)	–0.02 (0.01)	0.01 (0.03)	0.03 (0.04)	0.17 (0.04)	0.02 (0.01)	0.01 (0.01)
	Pretrained GNN	Test (ID)	0.77 (0.01)	0.78 (0.02)	0.85 (0.02)	0.85 (0.03)	0.75 (0.03)	0.78 (0.08)	0.67 (0.02)	0.86 (0.05)
		Test (OOD)	0.75 (0.04)	0.76 (0.05)	0.86 (0.02)	0.83 (0.02)	0.71 (0.04)	0.62 (0.06)	0.65 (0.02)	0.84 (0.07)
		Gap	0.02 (0.05)	0.03 (0.04)	–0.02 (0.02)	0.02 (0.03)	0.04 (0.04)	0.16 (0.05)	0.03 (0.02)	0.01 (0.05)

Examining Table 7, our first observation is that the ID performance differs across data sets, with mean ID test ROC-AUC ranging from 0.88 (HERG) to 0.67 (AMES). This indicates that inherent task difficulty varies substantially across molecular property prediction data sets. Since our focus in this study is on the relative performance drop of OOD compared to ID rather than the absolute ID performance, we ran all the experiments with the same default hyperparameters (Table S2) for reduced complexity and fair comparison. Note that these hyperparameters can lead to suboptimal performance on some data sets.

We found that the performance gap varied substantially across the data sets for some splitters compared to others. For example, for the scaffold splitter, the mean gap in Δ ROC-AUC ranged from 0.01 (CYP2C9, CYP2C19, CYP2D6, CYP3A4) to 0.04 (HERG), whereas for the max dissimilarity splitter, the gap was wider, ranging from −0.06 (CYP1A2) to 0.13 (CYP2C9). One-sample *t* tests against zero revealed that 74 out of 80 data set-splitter combinations showed statistically significant performance differences (*p* < 0.05; Bonferroni corrected).

To determine the extent to which these observations are model-dependent, we calculated these metrics for classical ML, GNN, and pretrained GNN models (Table 8 and Figure 6). One observation is that whenever the performance gap increased for classical ML models, we saw a corresponding increase in performance gap for the GNN-based models. In other words, when classical ML models are challenged by a particular data splitting method (showing larger performance gaps), GNN models are similarly challenged by that same splitting method. This suggests that both types of models follow the same trend when facing different splitting strategies.

For each splitter, we conducted one-way ANOVA tests on the performance gap data obtained from our repeated splits, with a significance level of α = 0.01 to determine whether there were statistically significant differences among classical ML, GNN, and pretrained GNN models. We found significant overall differences for the scaffold generic, molecular weight reverse, K-means, UMAP, Lo-Hi and DataSAIL splitters (*F*-statistics and *p*-values: *F* = 5.9, *p* = 2.95 × 10^–3^; *F* = 16.6, *p* = 8.06 × 10^–8^; *F* = 5.9, *p* = 2.93 × 10^–3^; *F* = 10.0, *p* = 4.74 × 10^–5^; *F* = 10.4, p = 3.24 × 10^–5^; and *F* = 6.1, *p* = 2.33 × 10^–3^, respectively). For all these cases, the GNN-based models were more robust compared to classical ML models and exhibited a smaller decline in performance. The largest robustness advantages emerged in chemically challenging splits such as Hi-Lo, UMAP clustering, and DataSAIL, where GNN-based models maintained significantly smaller performance degradation. Both GNNs and pretrained GNNs showed comparable robustness, with pretrained models showing slight advantages in some clustering-based splits.

Another observation is that the generalization to molecular weight reverse splitting (i.e., training on larger-weight molecules and testing on smaller ones) posed a harder challenge to all three types of models compared to molecular weight splitting (i.e., training on smaller molecules and testing on larger ones). This indicates that models can have a more accurate prediction and higher generalization capacity when tested on molecules larger than those they were trained on, compared to the case when they are tested on molecules smaller than those they were trained on.

We additionally provide the same tables and figures comparing ID and OOD performance based on the accuracy metric in the Supporting Information (Tables S5, S6, and Figures S7–S10). When considering the drop in accuracy, Lo-Hi and UMAP splitting caused the largest drops (0.080 and 0.074, respectively). For splitting strategies that showed statistically significant differences in performance drop between classical ML and GNN-based methods (i.e., all splitting strategies except DataSAIL), GNN-based methods demonstrated greater robustness.

Moreover, we show the results for ROC-AUC performance of individual models in Figure S11. GROVER emerged as the most robust model, followed by Weave and GEM, demonstrating superior generalization capabilities. In contrast, tree-based ensemble methods showed the poorest robustness, with XGBoost and Random Forest exhibiting the largest performance degradation under distribution shift.

#### Hit Rate as an Alternative Metric to ROC-AUC

While ROC-AUC and accuracy serve as standard classification metrics, virtual screening applications often require evaluation metrics that better reflect real-world drug discovery workflows. Hit rate, which measures the fraction of true actives among the top-ranked predictions, provides a more direct assessment of model performance in virtual screening scenarios where only a limited number of top-ranked compounds can be experimentally tested.

We calculated the top-100 hit rate for each model and splitting strategy. For each OOD test set, we ranked all molecules by their predicted probability of activity and selected the top 100 predictions. The hit rate was then calculated as the fraction of true active compounds within these top 100 predictions. This metric simulates the practical constraint in virtual screening where computational resources and experimental capacity limit the number of compounds that can be further evaluated.

3 $$\mathrm{Hit}\;\mathrm{Rate}\;\left(\%\right)=\frac{\mathrm{TP}}{\mathrm{TP}+\mathrm{FP}}\times 100$$

We first examined the relationship between hit rate and ROC-AUC across all splitting strategies (Figure 8). We observed moderate correlations between these metrics, with Pearson correlation coefficients ranging from *r* = 0.36 (molecular weight splitting) to *r* = 0.72 (Lo-Hi splitting). This finding aligns with previous observations that ROC-AUC and hit rate can show divergent behavior, particularly for challenging distribution shifts such as UMAP-based clustering splits.^48^

**Figure 8:**
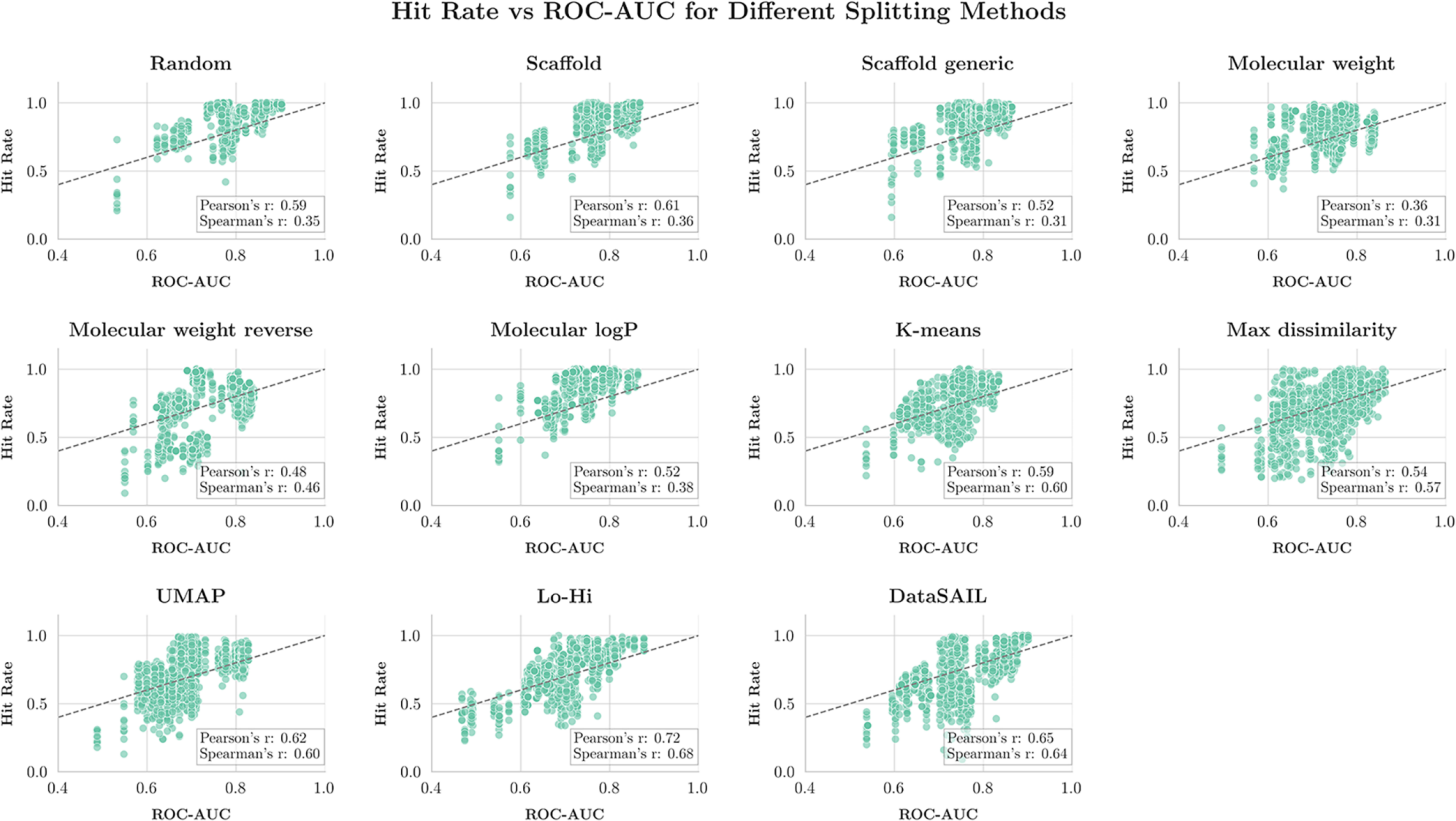
Correlation analysis between top-100 hit rate and ROC-AUC performance across all splitting strategies. Each data point represents the performance of a single model on one experimental replicate of a specific data set-splitter combination. The correlation patterns reveal the extent to which these two metrics provide consistent assessments of model performance.

Analysis of hit rate performance on OOD test sets revealed patterns largely consistent with our ROC-AUC findings, but with notable differences in magnitude (Figure S12). The largest performance drops in hit rate were observed for UMAP clustering (mean drop: 18.7%), and molecular weight reverse splitting (mean drop: 17.5%), while scaffold-based splits showed minimal degradation (mean drop: 1.8%).

Importantly, we observed substantial data set dependent variation in which splitting strategies proved most challenging. For instance, while UMAP clustering splitting showed the largest average hit rate drop across data sets, Lo-Hi splitting produced the most severe performance degradation specifically for the HERG data set (hit rate drop: 25.04 vs 15.68% for UMAP).

The moderate correlation between ROC-AUC and hit rate, combined with splitting-strategy-dependent variations, underscores the importance of using multiple evaluation metrics for assessing the performance of prediction models. The differential impact of splitting strategies on hit rate versus ROC-AUC suggests that model selection based solely on ROC-AUC optimization may not guarantee optimal performance in virtual screening scenarios, particularly when the target chemical space differs significantly from the training distribution.

### Analysis of the Relationship Between ID and OOD Performance

Previous reports suggest there is a strong linear positive correlation between ID and OOD performance, i.e., higher ID leads to higher OOD performance.^25,26^ Miller et al. introduced the accuracy-on-the-line concept, which shows empirically that there is a strong correlation between ID and OOD accuracy for a wide range of models and distribution shift in the computer vision domain.^25^ If this relationship holds, it can be used as a proxy for model selection for achieving higher OOD generalization. Since the introduction of the accuracy-on-the-line concept, many other studies have supported this idea^26,54,55^ or cast doubts on it.^29^ We investigated the on-the-line concept when applied to the molecular domain, in particular molecular property prediction, to understand whether this observation holds on data of the chemical domain. First, we investigated if there is a strong correlation (Pearson correlation and *R* ^2^), between ID and OOD performance for our splitting strategies, models, and data sets. Moreover, we probed for a connection between the ID-OOD correlation and the hardness of splitting strategies. In the following section, we analyze the ID-OOD correlation for classical ML, GNN, and pretrained GNN models when combining the results, ID and OOD ROC-AUC, for all the splitting strategies and data sets. Furthermore, we performed the same analysis considering each splitting strategy separately (Figure 9 and Table S7).

**Figure 9:**
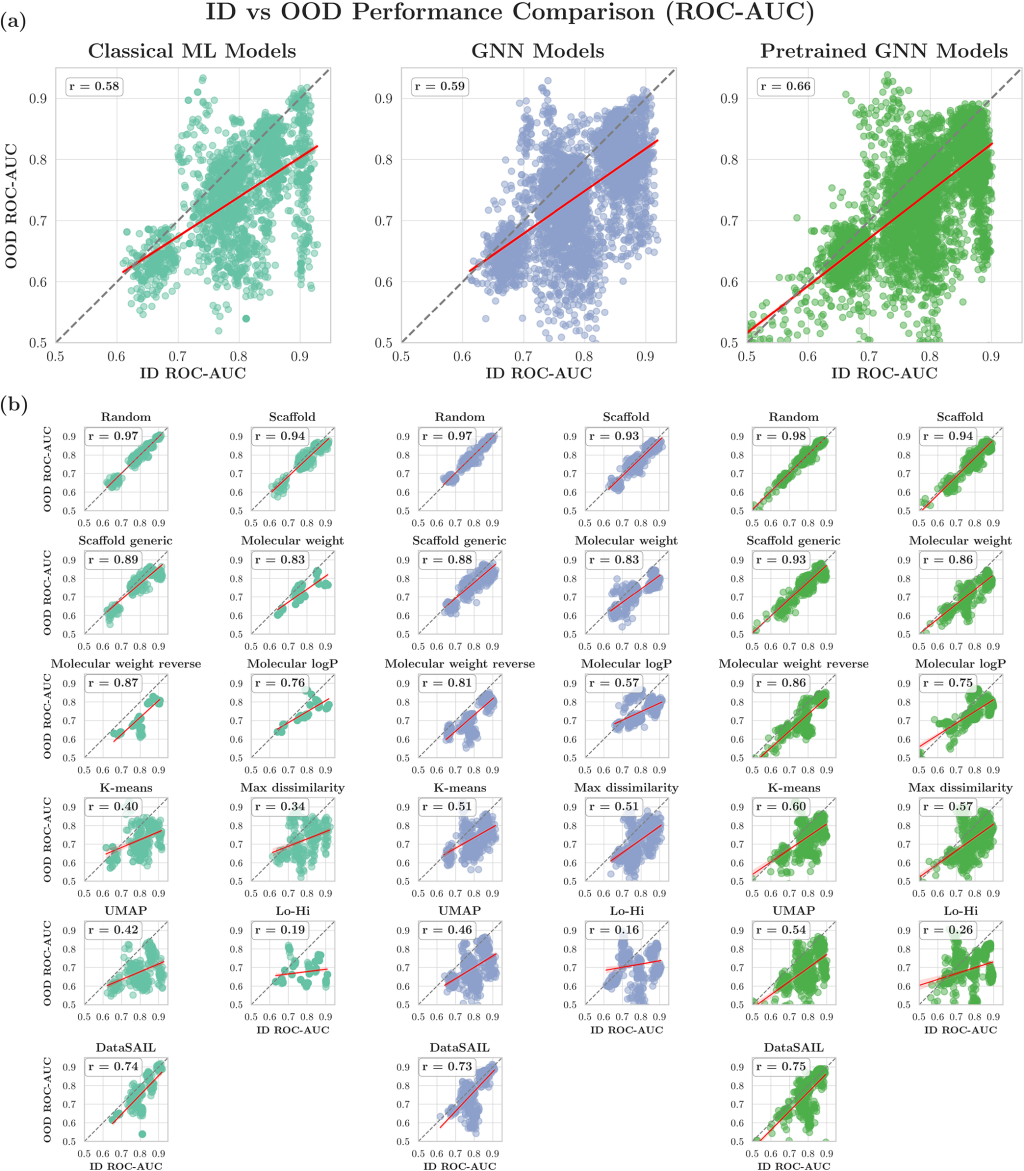
ID vs OOD performance measured by ROC-AUC (a) separately for classical ML, GNN, and pretrained GNN models and (b) separately for each individual splitting approach. Each data point corresponds to one model in our model pool. All plots aggregate results across all data sets. *r* represents the Pearson correlation coefficient.

Considering all the splitting strategies and data sets collectively, we observed a moderate positive correlation between ID and OOD ROC-AUC for classical ML (Pearson’s *r* = 0.58), GNNs (Pearson’s *r* = 0.59), and pretrained GNNs (Pearson’s *r* = 0.66) (Figure 9a). However, a more nuanced picture emerged when analyzing individual splitting strategies (Figure 9b). The correlation strength varied markedly across different splitting strategies, and all observed correlations were statistically significant (all *p* < 0.01) due to the large sample sizes (Classical ML: *n* = 240; GNNs: *n* = 400; Pretrained GNNs: *n* = 480). We observed a strong correlation between ID and OOD performance when using random and scaffold splitting (classical ML: Pearson’s *r* = 0.97 and 0.94; GNNs: Pearson’s *r* = 0.97 and 0.93; pretrained GNNs: Pearson’s *r* = 0.98 and 0.94, respectively), while using some other splitters, like K-means and Lo-Hi, showed substantially weaker relationships (classical ML: Pearson’s *r* = 0.40 and 0.19; GNNs: Pearson’s *r* = 0.51 and 0.16; pretrained GNNs: Pearson’s *r* = 0.60 and 0.26, respectively). These observations can be explained and understood based on the hardness of the splitting approach (see section Evaluation of the Hardness of Splitting Strategies). We observe that random and scaffold splitting are among the least challenging approaches (Figures 3 and 5), which means that they do not create a distinct chemical space for the OOD test set. For these approaches, the relationship between ID and OOD is strongly correlated. In contrast, K-means and Lo-Hi are among the “hardest” approaches, which can create an OOD test set with a distinguishing chemical space compared to the training set. In that case, there is a weaker correlation between ID and OOD ROC-AUC.

The fact that the relationship between ID and OOD performance is dependent on, and influenced by, the nature of the OOD data set generation strategy has important implications for model selection strategies. In cases where the chemical space of the OOD data is not far from that of the training data (measured by the distance between the OOD test and training set), model selection strategies based on the ID performance can work well and be efficient. However, as the OOD chemical space becomes more distant, the ID-OOD relationship becomes increasingly nonlinear and more nuanced, and model selection based on ID performance may not be the best model selection strategy. We observed cases where multiple models achieve similar ID performance but yield substantially different OOD results, highlighting that high ID performance does not guarantee strong OOD generalization.

We observed similar ID-OOD correlation patterns between classical ML, GNN, and pretrained GNN models across different splitting strategies. When a splitting strategy showed a high ID-OOD correlation for classical ML models, it typically showed a comparable correlation for GNN and pretrained GNN models. All three model types exhibited the strongest ID-OOD correlations with random, scaffold, and scaffold generic splitting (classical ML: Pearson’s *r* = 0.97, 0.94, and 0.89; GNNs: Pearson’s *r* = 0.97, 0.93, and 0.88; pretrained GNNs: Pearson’s *r* = 0.98, 0.94, and 0.93, respectively), and the weakest correlations with Lo-Hi splitting (classical ML: Pearson’s *r* = 0.19; GNNs: Pearson’s *r* = 0.16; pretrained GNNs: Pearson’s *r* = 0.26).

We further analyzed the relationship between ID and OOD performance at the individual data set level (Table S7) and found a more nuanced relationship across data sets. Taking max dissimilarity splitting as an example, we have seen varying correlations: positive correlation for AMES (in classical ML, GNN, and pretrained GNN models), negligible correlation for CYP2C19, and negative correlation for CYP3A4 (Figure 10). Three key factors contribute to the diverse patterns. First, the number of data points (ID vs OOD ROC-AUC) is insufficient for some data set-splitting combinations (30 for classical ML models, 50 for GNN models, and 60 for pretrained GNN models) to establish reliable linear relationships. Second, the narrow dynamic range of ID performance (between minimum and maximum ID ROC-AUC; classical ML: 0.056; GNNs: 0.077; pretrained GNNs: 0.156 averaged over the data sets) means prediction noise can dominate the observed patterns. Third, some data sets actually exhibit inverse ID-OOD correlations, which is in contradiction with the consensus trend. These cases need further investigation, particularly with larger sample sizes (more trained models) and a broader performance dynamic range.

**Figure 10:**
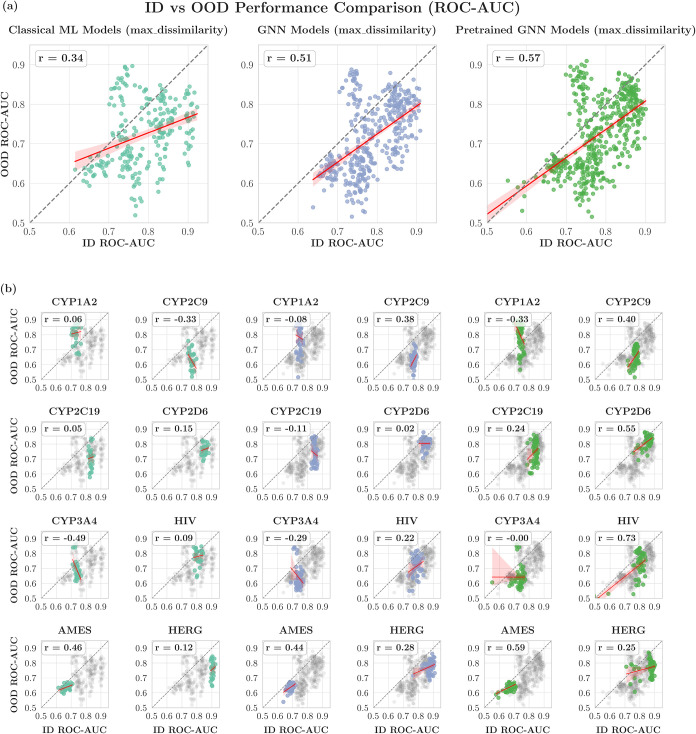
ID vs OOD performance measured by ROC-AUC for max dissimilarity splitter. The results are presented (a) separately for classical ML, GNN, and pretrained GNN models and (b) separately for each individual data set. Each data point corresponds to one model in our model pool. *r* represents the Pearson correlation coefficient.

### Limitations and Future Work

Several limitations should be acknowledged and addressed in future research.

#### Data Set Scale and Scope

The data sets used for benchmarking all contain between 5000 and 20,000 molecules with activity ratios of 30–70% (as described in section Data Sets). The generalizability of our findings to smaller data sets (e.g., <100 molecules, common in early drug discovery) or larger data sets (e.g., >100,000 molecules, as typical for high-throughput screening data set) requires further investigation. Additionally, this study focused exclusively on binary classification tasks for ADMET and bioactivity prediction, and extension to regression tasks or other molecular property domains remains unexplored.

An important characteristic of our data sets, which derived from publicly available data sets, is target promiscuity: the targets examined (primarily CYPs) are known to be promiscuous, resulting in many structurally distinct active compounds, and all data sets contain high proportions of active compounds (30–70%). While this promiscuity provides valuable diversity for model evaluation, it may introduce selection bias against scaffold split performance compared to pharmaceutical industry settings, where data sets typically contain higher proportions of congeneric series. The strongest advocates of scaffold split validation have previously come from industry, where researchers work with large congeneric series of compounds, suggesting our findings regarding scaffold split may not fully generalize to such contexts.

#### Model Configuration

To ensure fair comparison across all architectures, and avoid prohibitive computational burden, we employed fixed hyperparameters for each model type rather than data set-specific optimization. While this approach enables systematic comparison, it may lead to suboptimal performance for individual models on specific data sets, potentially affecting the absolute performance values and correlation patterns observed.

#### Evaluation Metrics

Our primary analysis focused on ROC-AUC and accuracy metrics. Although we investigated additional metrics such as top-100 hit rate for virtual screening relevance, the generalizability of our findings to other evaluation metrics commonly used in drug discovery (e.g., enrichment factors, or domain-specific metrics) requires systematic investigation.

## Conclusions

In this work, we trained over 11,000 ML models based on classical tabular methods (such as RF) as well as various graph neural networks, to investigate two research questions. First, we analyzed how distribution shifts in the training and test data affect the performance of the models. To this end, we explored ten distinct data splitting methods. Second, we investigated whether, and under what conditions, ID and OOD performance correlate.

According to both distance metrics employed in this work (Tanimoto distance and TMD), scaffold splitting and scaffold generic splitting consistently produced the least challenging test sets among the ten investigated splitters. These results show that scaffold splitting approaches can lead to overly optimistic performance estimates compared to more rigorous evaluation methods such as clustering based on molecular structures. Moreover, classical ML, GNN, and pretrained GNN models showed similar relative performance pattern when confronted with OOD data: splitting methods that challenged classical ML models (showing larger performance gaps) similarly challenged GNN-based methods. However, when performance drops differed significantly between classical ML and GNN-based models, GNN-based models demonstrated more robust behavior.

Regarding the relationship between ID and OOD performance, we observed that the strength of the correlation substantially depends on the splitting strategy used for generating OOD data. OOD data with the most similar chemical space to the training data (measured based on the distance metric) led to a strong correlation between ID-OOD performance. However, as chemical space similarity decreased, this correlation weakened substantially. This observation indicates that ID performance is not always a good proxy and criterion for model selection when seeking the best generalization capacity.

Overall, this benchmarking study not only advances our understanding of distribution shifts in molecular ML but also provides guidance for practitioners to design more reliable evaluation protocols.

## Supplementary Material



## Data Availability

All data utilized in this work originates from the Therapeutics Data Commons (TDC) database and is available from https://tdcommons.ai. The source code of the approach presented in this work is available from https://github.com/HFooladi/ALineMol

## Notes

Detailed protocols for data set preprocessing, complete model training parameters and hyperparameter selection methodology, statistical properties and characteristics of all data sets, comprehensive tables of accuracy metrics for all experiments described in the main manuscript, additional visualizations of model performance (PDF)

## References

[1] Schneider P., Walters W. P., Plowright A. T. (2020). Rethinking Drug Design
in the Artificial Intelligence Era. Nat. Rev.
Drug Discovery.

[2] Askr H., Elgeldawi E., Aboul Ella H., Elshaier Y. A. M. M., Gomaa M. M., Hassanien A. E. (2023). Deep Learning
in Drug Discovery:
An Integrative Review and Future Challenges. Artif. Intell. Rev..

[3] Yang K., Swanson K., Jin W., Coley C., Eiden P., Gao H., Guzman-Perez A., Hopper T., Kelley B., Mathea M., Palmer A., Settels V., Jaakkola T., Jensen K., Barzilay R. (2019). Analyzing
Learned Molecular Representations for Property
Prediction. J. Chem. Inf. Model..

[4] Vamathevan J., Clark D., Czodrowski P., Dunham I., Ferran E., Lee G., Li B., Madabhushi A., Shah P., Spitzer M., Zhao S. (2019). Applications
of Machine Learning in Drug Discovery and Development. Nat. Rev. Drug Discovery.

[5] Paul D., Sanap G., Shenoy S., Kalyane D., Kalia K., Tekade R. K. (2021). Artificial Intelligence
in Drug Discovery and Development. Drug Discovery
Today.

[6] Hendrycks, D. ; Dietterich, T. Benchmarking Neural Network Robustness to Common Corruptions and Perturbations. In Proceedings of the International Conference on Learning Representations, 2019. 10.48550/arXiv.1903.12261.

[7] Torralba, A. ; Efros, A. A. Unbiased Look at Dataset Bias. In Proceedings of the IEEE Conference on Computer Vision and Pattern Recognition; Colorado Springs: CO, USA, 2011; pp 1521–1528. 10.1109/CVPR.2011.5995347.

[8] Recht, B. ; Roelofs, R. ; Schmidt, L. ; Shankar, V. Do ImageNet classifiers generalize to ImageNet? In Proceedings of the International Conference on Machine Learning 2019; pp 5389–5400. 10.48550/arXiv.1902.10

[9] Geirhos R., Jacobsen J.-H., Michaelis C., Zemel R., Brendel W., Bethge M., Wichmann F. A. (2020). Shortcut learning in deep neural
networks. Nat. Mach. Intell..

[10] Scalia G., Grambow C. A., Pernici B., Li Y.-P., Green W. H. (2020). Evaluating
Scalable Uncertainty Estimation Methods for Deep Learning-Based Molecular
Property Prediction. J. Chem. Inf. Model..

[11] Tossou P., Wognum C., Craig M., Mary H., Noutahi E. (2024). Real-World
Molecular Out-Of-Distribution: Specification and Investigation. J. Chem. Inf. Model..

[12] Ji, Y. DrugOOD: Out-of-Distribution (OOD) Dataset Curator and Benchmark for AI-aided Drug Discovery - A Focus on Affinity Prediction Problems with Noise Annotations. arXiv, 2022. 10.48550/arXiv.2201.09637.

[13] Breiman L. (2001). Random Forests. Mach. Learn..

[14] Battaglia, P. W. ; Hamrick, J. B. ; Bapst, V. ; Sanchez-Gonzalez, A. ; Zambaldi, V. ; Malinowski, M. ; Tacchetti, A. ; Raposo, D. ; Santoro, A. ; Faulkner, R. Relational Inductive Biases, Deep Learning, and Graph Networks. arXiv 2018, 10.48550/arXiv.1806.01261.

[15] Gulrajani, I. ; Lopez-Paz, D. In Search of Lost Domain Generalization. In Proceedings of the International Conference on Learning Representations, 2021. 10.48550/arXiv.2007.01434.

[16] Vapnik, V. Statistical Learning Theory Wiley. New York, 1998; Vol. 2, pp 831–842.

[17] Arjovsky, M. ; Bottou, L. ; Gulrajani, I. ; Lopez-Paz, D. Invariant Risk Minimization. arXiv, 2019. 10.48550/arXiv.1907.02893.

[18] Sagawa, S. ; Koh, P. W. ; Hashimoto, T. B. ; Liang, P. Distributionally Robust Neural Networks. In Proceedings of the International Conference on Learning Representations, 2020. 10.48550/arXiv.1911.08731.

[19] Sun, B. ; Saenko, K. Deep CORAL: Correlation Alignment for Deep Domain Adaptation. In Computer Vision - ECCV 2016 Workshops., Lecture Notes in Computer Science; Springer International Publishing, 2016; Vol. 9915, p 443 10.1007/978-3-319-49409-8_35.

[20] Huang, Z. ; Wang, H. ; Xing, E. P. ; Huang, D. Self-Challenging Improves Cross-Domain Generalization. In Computer Vision - ECCV 2020, Lecture Notes in Computer Science; Springer International Publishing, 2020; Vol. 2020, pp 124–140 10.1007/978-3-030-58536-5_8.

[21] Xu M., Zhang J., Ni B., Li T., Wang C., Tian Q., Zhang W. (2020). Adversarial Domain
Adaptation with
Domain Mixup. Proc. AAAI Conf. Artif. Intell..

[22] Bemis G. W., Murcko M. A. (1996). The Properties of Known Drugs. 1.
Molecular Frameworks. J. Med. Chem..

[23] Wu Z., Ramsundar B., Feinberg E. N., Gomes J., Geniesse C., Pappu A. S., Leswing K., Pande V. (2018). MoleculeNet: A Benchmark
for Molecular Machine Learning. Chem. Sci..

[24] Guo, Q. ; Hernandez-Hernandez, S. ; Ballester, P. J. Scaffold Splits Overestimate Virtual Screening Performance. arXiv, 2024. 10.48550/arXiv.2406.00873.

[25] Miller, J. P. ; Taori, R. ; Raghunathan, A. ; Sagawa, S. ; Koh, P. W. ; Shankar, V. ; Liang, P. ; Carmon, Y. ; Schmidt, L. Accuracy on the Line: On the Strong Correlation Between Out-of-Distribution and In-Distribution Generalization. In Proceedings of the International Conference on Machine Learning, 2021; pp 7721–7735. 10.48550/arXiv.2107.04649.

[26] Baek, C. ; Jiang, Y. ; Raghunathan, A. ; Kolter, Z. Agreement-on-the-Line: Predicting the Performance of Neural Networks under Distribution Shift. arXiv, 2022. 10.48550/arXiv.2206.13089.

[27] Vedantam, R. ; Lopez-Paz, D. ; Schwab, D. J. An Empirical Investigation of Domain Generalization with Empirical Risk Minimizers. In Proceedings of the Neural Information Processing Systems, 2021; pp 28131–28143. PMC9806840 36597462

[28] Teney, D. ; Lin, Y. ; Oh, S. J. ; Abbasnejad, E. ID and OOD Performance Are Sometimes Inversely Correlated on Real-world Datasets. arXiv, 2022. 10.48550/arXiv.2209.00613.

[29] Sanyal, A. ; Hu, Y. ; Yu, Y. ; Ma, Y. ; Wang, Y. ; Schölkopf, B. Accuracy on the Wrong Line: On the Pitfalls of Noisy Data for OOD Generalisation. In Proceedings of the International Conference on Machine Learning, Next Generation of AI Safety Workshop, 2024. 10.48550/arXiv.2406.19049.

[30] Huang K., Fu T., Gao W., Zhao Y., Roohani Y., Leskovec J., Coley C. W., Xiao C., Sun J., Zitnik M. (2022). Artificial
intelligence foundation for therapeutic science. Nat. Chem. Biol..

[31] Veith H., Southall N., Huang R., James T., Fayne D., Artemenko N., Shen M., Inglese J., Austin C. P., Lloyd D. G., Auld D. S. (2009). Comprehensive Characterization
of
Cytochrome P450 Isozyme Selectivity across Chemical Libraries. Nat. Biotechnol..

[32] Xu C., Cheng F., Chen L., Du Z., Li W., Liu G., Lee P. W., Tang Y. (2012). In Silico Prediction of Chemical
Ames Mutagenicity. J. Chem. Inf. Model..

[33] Karim A., Lee M., Balle T., Sattar A. (2021). CardioTox Net: ARobust Predictor
for hERG Channel Blockade Based on Deep Learning Meta-Feature Ensembles. J. Cheminf..

[34] Kipf, T. N. ; Welling, M. Semi-Supervised Classification with Graph Convolutional Networks. In Proceedings of the International Conference on Learning Representations, 2017. 10.48550/arXiv.1609.02907.

[35] Veličković, P. ; Cucurull, G. ; Casanova, A. ; Romero, A. ; Liò, P. ; Bengio, Y. Graph Attention Networks. In Proceedings of the International Conference on Learning Representations, 2018. 10.48550/arXiv.1710.10903.

[36] Gilmer, J. ; Schoenholz, S. S. ; Riley, P. F. ; Vinyals, O. ; Dahl, G. E. Neural Message Passing for Quantum Chemistry. In Proceedings of the International Conference on Machine Learning, 2017; pp 1263–1272. 10.48550/arXiv.1704.01212.

[37] Xiong Z., Wang D., Liu X., Zhong F., Wan X., Li X., Li Z., Luo X., Chen K., Jiang H., Zheng M. (2020). Pushing the Boundaries of Molecular
Representation for Drug Discovery
with the Graph Attention Mechanism. J. Med.
Chem..

[38] Kearnes S., McCloskey K., Berndl M., Pande V., Riley P. (2016). Molecular
Graph Convolutions: Moving beyond Fingerprints. J. Comput.-Aided Mol. Des..

[39] Xu, K. ; Hu, W. ; Leskovec, J. ; Jegelka, S. How Powerful Are Graph Neural Networks. In Proceedings of the International Conference on Learning Representations, 2019. 10.48550/arXiv.1810.00826.

[40] Hu, W. ; Liu, B. ; Gomes, J. ; Zitnik, M. ; Liang, P. ; Pande, V. ; Leskovec, J. Strategies for Pre-Training Graph Neural Networks. In Proceedings of the International Conference on Learning Representations. 2020. 10.48550/arXiv.1905.12265.

[41] Fang X., Liu L., Lei J., He D., Zhang S., Zhou J., Wang F., Wu H., Wang H. (2022). Geometry-enhanced molecular
representation learning for property prediction. Nat. Mach. Intell..

[42] Rong, Y. ; Bian, Y. ; Xu, T. ; Xie, W. ; Wei, Y. ; Huang, W. ; Huang, J. Self-Supervised Graph Transformer on Large-Scale Molecular Data. In Advances in Neural Information Processing Systems; Curran Associates, Inc., 2020; pp 12559–12571 10.48550/arXiv.2007.02835.

[43] Vapnik, V. ; Golowich, S. ; Smola, A. Support Vector Method for Function Approximation, Regression Estimation and Signal Processing. In Proceedings of the Neural Information Processing Systems, 1996; pp 281–287.

[44] Schölkopf, B. ; Smola, A. J. Learning with Kernels: Support Vector Machines, Regularization, Optimization, and Beyond; The MIT Press, 2001 10.7551/mitpress/4175.001.0001.

[45] Chen, T. ; Guestrin, C. XGBoost: A Scalable Tree Boosting System. In Proceedings of the 22nd ACM SIGKDD International Conference on Knowledge Discovery and Data Mining; ACM: San Francisco California USA, 2016; pp 785–794 10.1145/2939672.2939785.

[46] Li M., Zhou J., Hu J., Fan W., Zhang Y., Gu Y., Karypis G. (2021). DGL-LifeSci: An Open-Source Toolkit for Deep Learning
on Graphs in Life Science. ACS Omega.

[47] Pedregosa F. (2011). Scikit-Learn: Machine
Learning in Python. J.
Mach. Learn. Res..

[48] Guo Q., Hernandez-Hernandez S., Ballester P. J. (2025). UMAP-based Clustering Split for Rigorous
Evaluation of AI Models for Virtual Screening on Cancer Cell Lines*. J. Cheminf..

[49] Steshin, S. Lo-Hi: Practical ML Drug Discovery Benchmark. In Thirty-seventh Conference on Neural Information Processing Systems Datasets and Benchmarks Track, 2023.

[50] Joeres R., Blumenthal D. B., Kalinina O. V. (2025). Data splitting to avoid information
leakage with DataSAIL. Nat. Commun..

[51] Sheridan R. P., Feuston B. P., Maiorov V. N., Kearsley S. K. (2004). Similarity to Molecules
in the Training Set Is a Good Discriminator for Prediction Accuracy
in QSAR. J. Chem. Inf. Comput. Sci..

[52] Chuang, C.-Y. ; Jegelka, S. Tree Mover’s Distance: Bridging Graph Metrics and Stability of Graph Neural Networks. In Proceedings of the Neural Information Processing Systems, 2022. 10.48550/arXiv.2210.01906.

[53] Tukey J. W. (1949). Comparing
Individual Means in the Analysis of Variance. Biometrics.

[54] Kim, E. ; Sun, M. ; Baek, C. ; Raghunathan, A. ; Kolter, J. Z. Test-Time Adaptation Induces Stronger Accuracy and Agreement-on-the-Line. In Proceedings of the Neural Information Processing Systems, 2024. 10.48550/arXiv.2310.04941.

[55] Shi, J. ; Gare, G. R. ; Tian, J. ; Chai, S. ; Lin, Z. ; Vasudevan, A. B. ; Feng, D. ; Ferroni, F. ; Kong, S. ; Ramanan, D. LCA-on-the-line: Benchmarking out of Distribution Generalization with Class Taxonomies. In Proceedings of the Neural Information Processing Systems Workshop on Distribution Shifts: New Frontiers with Foundation Models, 2024. 10.48550/arXiv.2407.16067.

