# Supplementary material for: Evaluating Machine Learning Models for Molecular Property Prediction: Performance and Robustness on Out-of-Distribution Data
Source: J Chem Inf Model. 2025 Sep 15;65(19):9871–91. doi: 10.1021/acs.jcim.5c00475 (PMC12529777; doi:10.1021/acs.jcim.5c00475)
Supplement: Supplementary file 1 [file ci5c00475_si_001.pdf]

SUPPORTING INFORMATION

Evaluating Machine Learning Models for Molecular Property Prediction: Performance and Robustness on Out-of-Distribution Data

Hosein Fooladi,[†,‡,¶] Thi Ngoc Lan Vu,[†,‡,¶] Miriam Mathea,[§] and Johannes Kirchmair[*,†,‡,¶]

†*Department of Pharmaceutical Sciences, Division of Pharmaceutical Chemistry, Faculty of Life Sciences, University of Vienna, Josef-Holaubek-Platz 2, 1090 Vienna, Austria*

‡*Christian Doppler Laboratory for Molecular Informatics in the Biosciences, Department for Pharmaceutical Sciences, University of Vienna, 1090 Vienna, Austria*

¶*Vienna Doctoral School of Pharmaceutical, Nutritional and Sport Sciences (PhaNuSpo), University of Vienna, 1090 Vienna, Austria*

§*BASF SE, Ludwigshafen, 67056, Germany*

E-mail: johannes.kirchmair@univie.ac.at

Contents

1 Molecular Standardization **S3**

2 Model Training and Selection **S5**

3 Hyperparameter Selection **S5**

4 Pretrained Graph Neural Network Models **S6**

4.1 Overview S6

4.2 GIN-based Pretrained Models (DGL-LifeSci) S7

4.2.1 Model Architecture S7

4.2.2 Pretraining Strategy S7

4.2.3 Self-supervised Tasks S7

4.3 GEM (Geometry-Enhanced Molecular Representation Learning) S8

4.3.1 Model Architecture S8

4.3.2 Pretraining Strategy S9

4.4 GROVER (Self-supervised Graph Transformer) S9

4.4.1 Model Architecture S9

4.4.2 Pretraining Strategy S9

5 Supplementary Figures **S10**

6 Supplementary Tables **S21**

References **S35**

1 Molecular Standardization

The `standardization_pipeline` function takes a DataFrame that must have two columns: `'smiles'` (containing SMILES strings representing molecules) and `'label'` (containing the corresponding labels/properties), and performs three main operations:

1. **Standardize SMILES** (using `standardize_smiles` function):
   - Converts each SMILES string into a standardized Molecule object using RDKit
   - Performs several cleaning operations:
     - Removes hydrogens

$$\text{Molecule} \xrightarrow{\text{RemoveHs}} \text{No Explicit Hydrogens} \tag{1}$$

     - Disconnects metal complexes (optional)

$$\text{No Explicit Hydrogens} \xrightarrow[\text{(optional)}]{\text{MetalDisconnector}} \text{Disconnected Metals} \tag{2}$$

     - Normalizes functional groups

$$\text{Disconnected Metals} \xrightarrow{\text{Normalizer}} \text{Normalized Structure} \tag{3}$$

     - Reionizes acids/bases

$$\text{Normalized Structure} \xrightarrow{\text{Reionizer}} \text{Adjusted Ionization} \tag{4}$$

     - Picks the largest fragment

$$\text{Adjusted Ionization} \xrightarrow{\text{LargestFragmentChooser}} \text{Desalted Structure} \tag{5}$$

- Neutralizes charges

$$\text{Desalted Structure} \xrightarrow{\text{Uncharger}} \text{Neutral Molecule} \tag{6}$$

- Canonicalizes tautomers (optional, but true by default)

$$\text{Neutral Molecule} \xrightarrow[\text{(optional)}]{\text{TautomerEnumerator}} \text{Canonical Tautomer} \tag{7}$$

2. **Remove Duplicates** (using `drop_duplicates` function):
   - Removes any rows with NaN values in SMILES
   - Removes conflicting duplicates (same molecule but different labels)
   - Keeps only one instance of molecules that are identical and have the same label

3. **Final Cleanup**:
   - Keeps only the `'canonical_smiles'` and `'label'` columns

Detailed Standardization Operations Each transformation in the standardization process serves a specific purpose:

Metal Disconnection: Metal-organic complex $\longrightarrow$ Disconnected metals and ligands

Separates metals from organic ligands for better standardization of the organic components.

Normalization: Varied functional groups $\longrightarrow$ Canonical representations

Converts functional groups to their standard forms according to predefined transformation rules.

Reionization: Arbitrary protonation state $\longrightarrow$ Preferred protonation state

Adjusts acids and bases to their preferred ionization states based on pKa values.

Largest Fragment Selection: Molecule with salts/counter-ions $\longrightarrow$ Main molecular entity

Selects the largest fragment to remove salts and counter-ions, with preference for organic components.

Uncharging: Charged molecule $\longrightarrow$ Neutral form

Neutralizes formal charges when possible to obtain a neutral representation.

Tautomer Canonicalization: Arbitrary tautomer $\longrightarrow$ Canonical tautomer form

Converts all possible tautomers to a single canonical form for consistent representation.

The source code for preprocessing the data sets is available in our GitHub repository: `https://github.com/HFooladi/ALineMol`

2 Model Training and Selection

All models, including both classical machine learning algorithms and graph neural networks (GNNs), were trained exclusively on the training set. In-distribution (ID) validation data was used for early stopping during training of GNNs that originate from DGL-LifeSci. The ROC-AUC metric was employed as the early stopping criterion. Specifically, training was terminated if the ROC-AUC on the ID validation set failed to improve over a predefined number of iterations (the patience parameter was set to 30 by default), and the model with the best validation performance was retained.

For the GEM[1] and GROVER[2] models, training was performed for the maximum number of epochs, and the model achieving the highest ROC-AUC on the ID validation set was selected for subsequent analysis and prediction tasks.

3 Hyperparameter Selection

Since the primary objective of this study is to compare model performance between ID and OOD data, we were not primarily concerned with selecting hyperparameters that achieve

optimal performance on each dataset. Given that we conducted dozens of experiments across different datasets, splitters, and models, running hyperparameter optimization for each experimental setting would have been computationally prohibitive.

Specific Selection Methods:

- **Classical ML models (Random Forest, SVM, XGBoost):** We performed grid search hyperparameter optimization on an external validation dataset and applied the best-performing hyperparameters consistently across all experiments.
- **Graph Neural Networks from DGL-LifeSci:**[3] We used the default hyperparameters provided in the DGL-LifeSci package, which have been optimized for molecular property prediction tasks through extensive validation by the software developers.
- **Pre-trained models (GEM, GROVER):** We employed the default hyperparameters for fine-tuning as specified in their respective software repositories, which represent the authors' recommended settings based on their original publications and benchmarking studies.

All hyperparameters are provided in Table S2. We acknowledge that this approach may not yield optimal performance for individual datasets; however, it enables robust and fair comparison of generalization capabilities across different model architectures and data splitting strategies, which is the central contribution of our work.

4 Pretrained Graph Neural Network Models

4.1 Overview

This section provides information about the pretrained Graph Neural Network (GNN) models used in our study.

4.2 GIN-based Pretrained Models (DGL-LifeSci)

4.2.1 Model Architecture

Backbone: Graph Isomorphism Network (GIN) architecture, which has been shown to be as powerful as the Weisfeiler-Lehman test in distinguishing non-isomorphic graphs.

4.2.2 Pretraining Strategy

Following the methodology of Hu et al.,[4] these models employ a two-stage pretraining approach:

Stage 1: Node-level Self-supervised Pretraining

- **Data set:** 2 million unlabeled molecules sampled from the ZINC15 database
- **Objective:** Learn local molecular representations through self-supervised tasks

Stage 2: Graph-level Supervised Pretraining

- **Data set:** ChEMBL dataset containing 456,000 molecules with 1,310 diverse biochemical assays
- **Objective:** Learn graph-level and global representations for molecular property prediction

4.2.3 Self-supervised Tasks

We utilized four variants of GIN models, each pretrained with different self-supervised strategies:

1. **Context Prediction (ContextPred)**
    - **Task:** Predict the surrounding graph structure from local neighborhoods
    - **Method:** Uses negative sampling to learn whether neighborhood and context graphs belong to the same node

- **Hyperparameters:** K-hop neighborhoods (K=5), context radius r1=4, r2=r1+3

2. **Deep Graph Infomax (InfoMax)**
   - **Task:** Maximize mutual information between node-level and graph-level representations
   - **Method:** Distinguishes whether node-graph pairs come from the same graph or different graphs

3. **Attribute Masking (AttrMasking)**
   - **Task:** Predict masked node/edge attributes from neighboring structure
   - **Method:** Randomly masks 15% of the atom types and bond features, then predicts them
   - **Benefit:** Learns chemical rules such as valency and functional group properties

4. **Edge Prediction (EdgePred)**
   - **Task:** Predict the existence of edges between nodes
   - **Method:** Binary classification of whether edges exist between node pairs

4.3 GEM (Geometry-Enhanced Molecular Representation Learning)

4.3.1 Model Architecture

Backbone: The authors proposed GeoGNN architecture.[1] It uses the Aggregate and Combine function from Graph Isomorphism Network (GIN) architecture, with the addition of residual connection, layer normalization, and graph normalization for enhancement.

4.3.2 Pretraining Strategy

Data set: 20 million unlabeled molecules sampled from the ZINC15 database

Pretraining Tasks:

- **3D Geometry Prediction:** Self-supervised learning of 3D local spatial relationships between atoms by using (1) the bond lengths prediction; (2) the bond angles prediction.
- **Distance Prediction:** Learning inter-atomic distances in 3D space for learning global spatial structure.
- **Graph-level Prediction:** Predict the molecular fingerprints for the graph-level tasks. Each bit in the fingerprint is assumed to be a binary label, and the task can be formulated as a binary classification.

4.4 GROVER (Self-supervised Graph Transformer)

4.4.1 Model Architecture

Backbone: GNN Transformer, a hybrid architecture combining Message Passing Networks with Transformer-style architecture.[2]

4.4.2 Pretraining Strategy

Data set: 11 million unlabeled molecules sampled from ZINC15 and ChEMBL data sets

Pretraining Tasks:

1. **Contextual Property Prediction:**
2. **Graph-level Motif Prediction:** motif (functional group) prediction, which can be formulated as multi-label classification.

5 Supplementary Figures

List of Supplementary Figures:

Figure S1. Chemical space visualization based on t-SNE method, showing data set-specific molecules against the background of all unique molecules.

Figure S2. Chemical space visualization based on t-SNE method, distinguishing active (green) and inactive (orange) molecules in each data set against the background of all unique molecules.

Figure S3. Distribution of physicochemical properties across different data sets.

Figure S4. Test size ratio (ID and OOD) for each splitting strategy, aggregated across data sets and replicates.

Figure S5. Activity ratio, training and OOD test set for each data set.

Figure S6. Ratio of unique scaffolds to total number of molecules for each data set.

Figure S7. Heatmap visualization of in-distribution and out-of-distribution performance in terms of ROC-AUC across data sets and splitters.

Figure S8. Heatmap visualization of in-distribution and out-of-distribution performance in terms of Accuracy across data sets and splitters.

Figure S9. Heatmap showing performance drop measured by Accuracy for different data sets and splitters, averaged across all models and replicates.

Figure S10. Comprehensive heatmap of performance drops measured by Accuracy across different data sets, splitters, and models.

Figure S11. Performance measured with ROC-AUC for all models across different splitting strategies.

Figure S12. Top 100 hit rate performance across different splitting strategies.

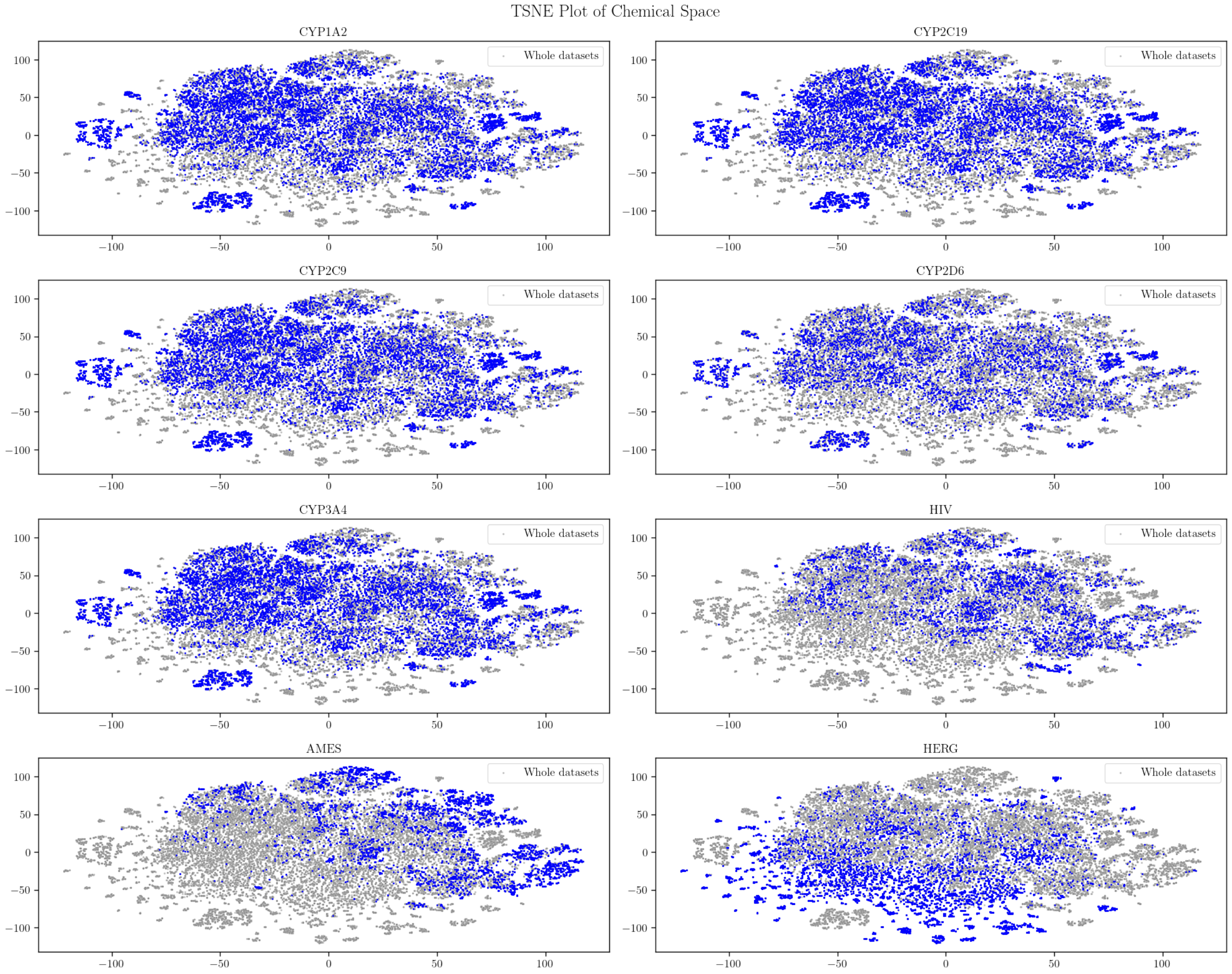

Figure S1: Chemical space visualization based on the t-SNE method. Gray points (background) are the unique molecules in all the data sets. Blue points (darker points) are the molecules in each particular data set.

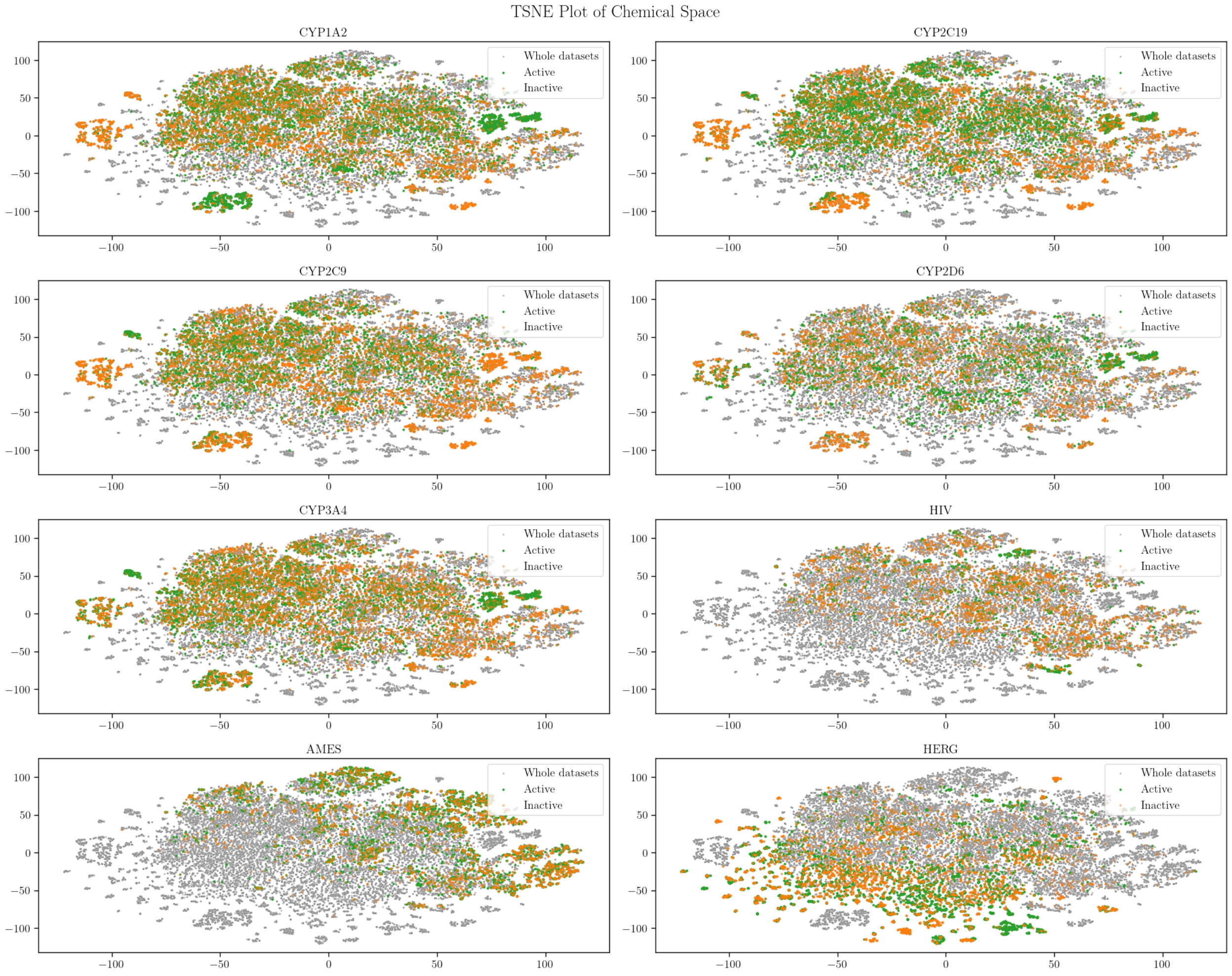

Figure S2: Chemical space visualization based on the t-SNE method. Gray points (background) are the unique molecules in all the data sets. Green points are the active molecules in each particular data set. Orange points are inactive molecules in each data set.

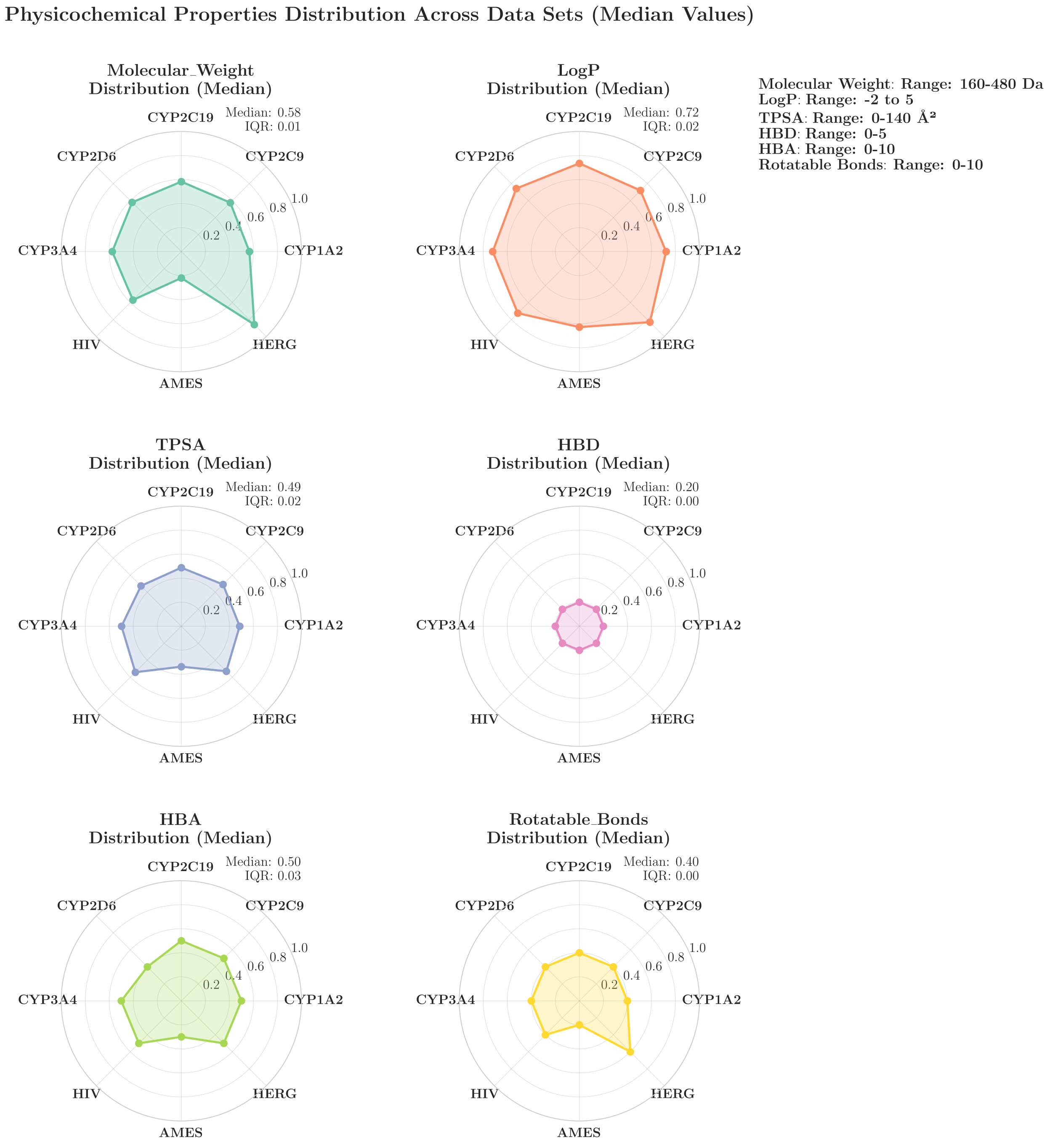

Figure S3: Physicochemical properties distribution across different data sets.

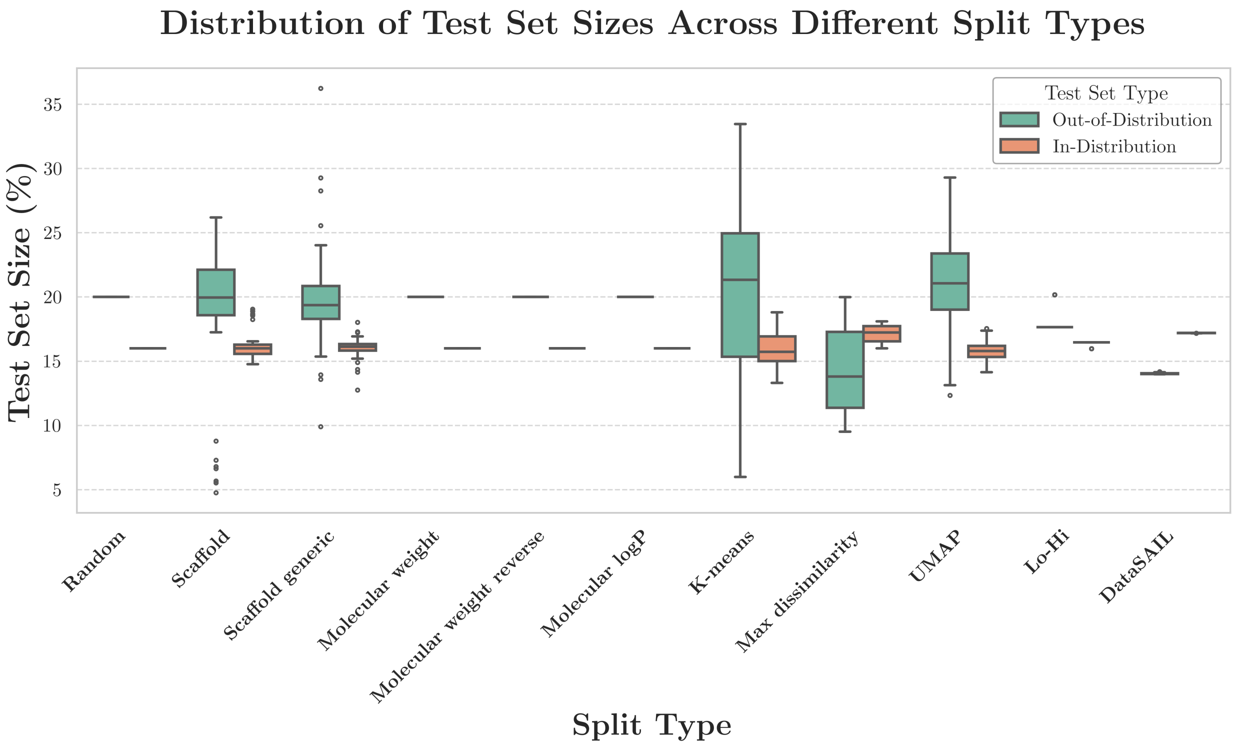

Figure S4: Test size ratio, ID and OOD, for each splitting strategy. Numbers have been aggregated across the data sets and replicates.

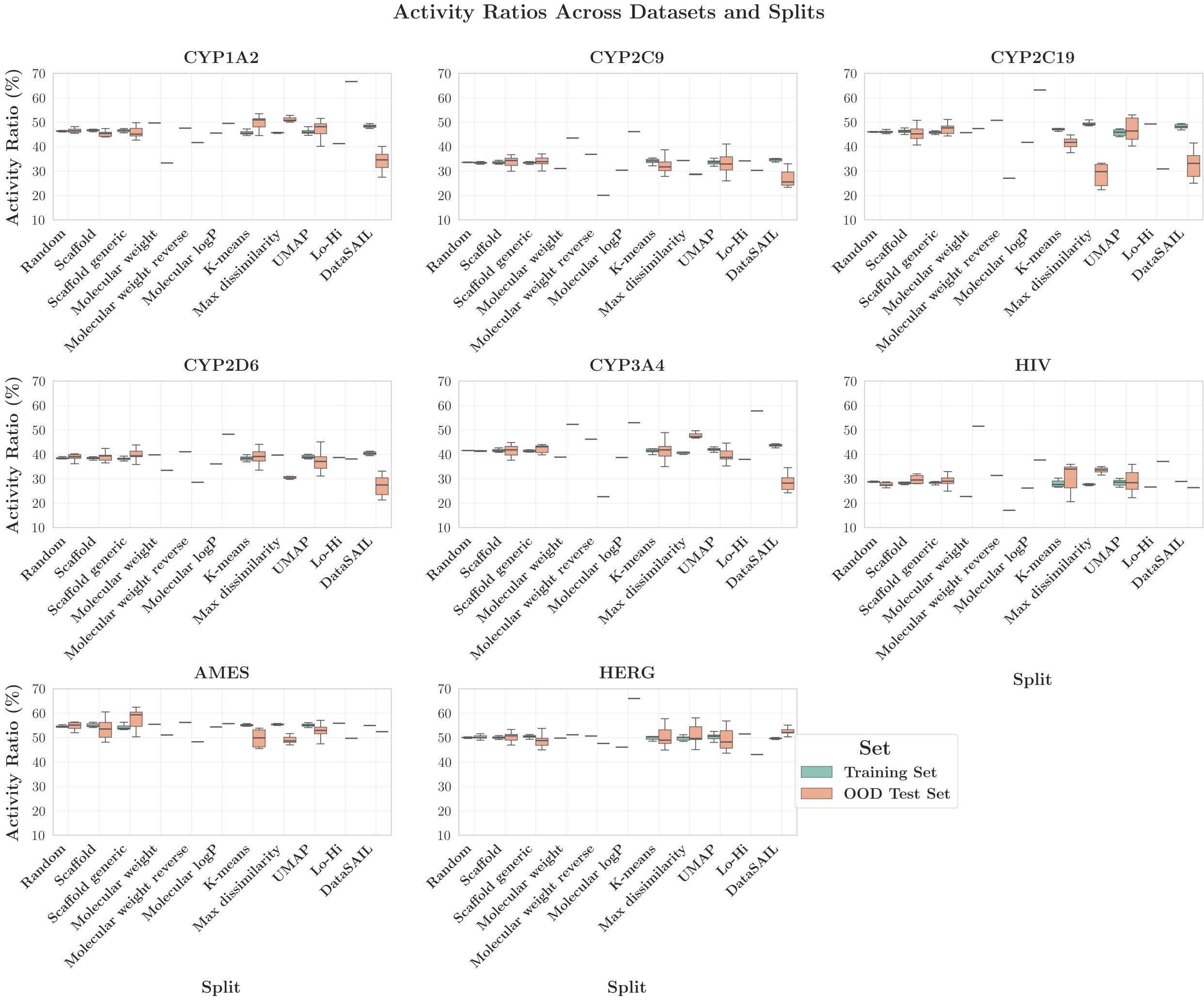

Figure S5: Activity ratio, training and OOD tests set for each data set.

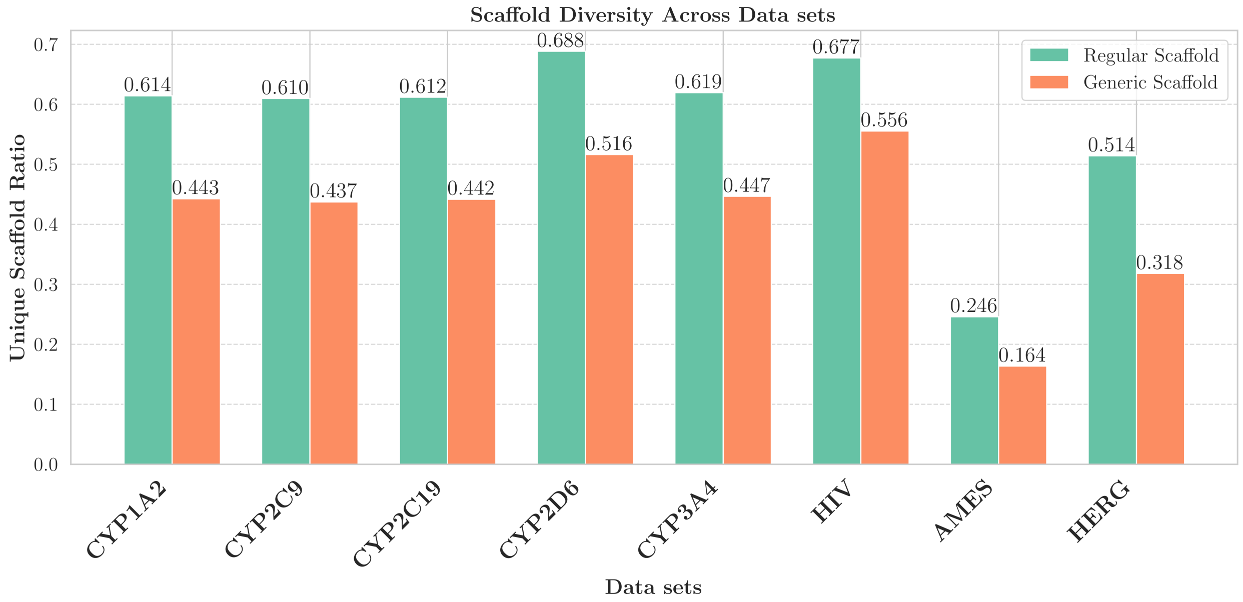

Figure S6: Ratio of unique scaffolds to the total number of molecules for each data sets.

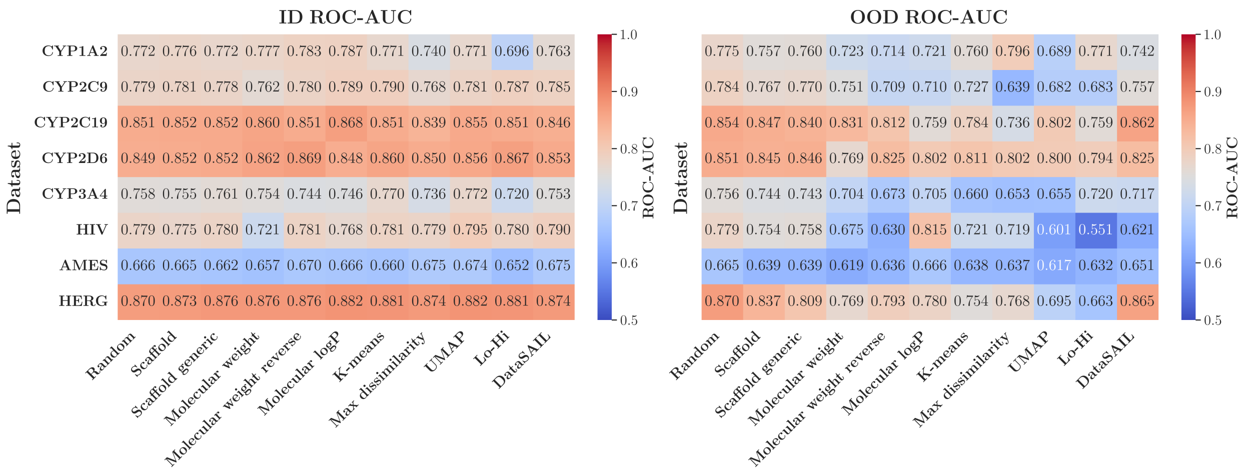

Figure S7: In-distribution (ID) and out-of-distribution (OOD) performance on test sets for each data sets and splitters in terms of ROC-AUC. Results are averaged across all the models and replicates.

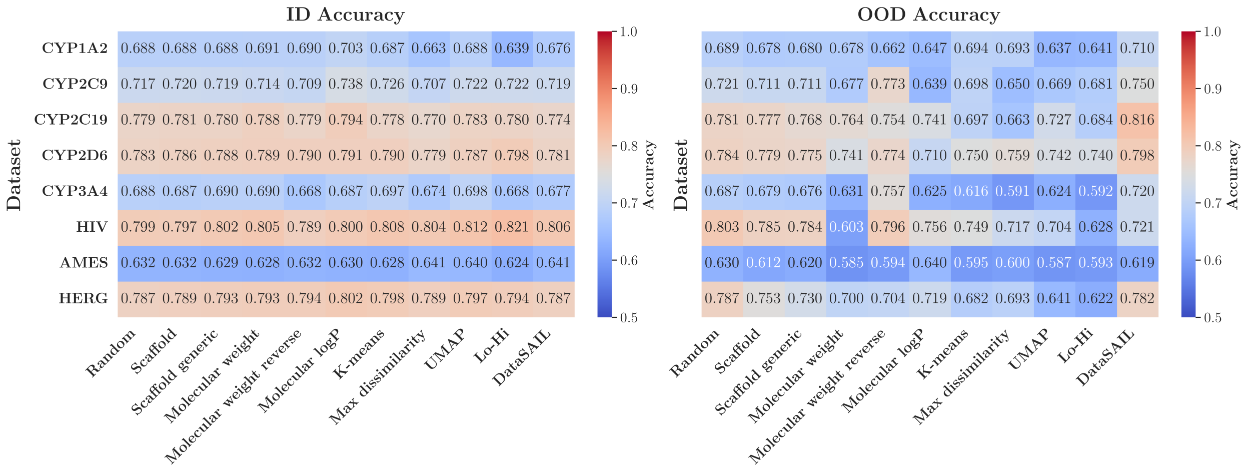

Figure S8: In-distribution (ID) and out-of-distribution (OOD) performance on test sets for each data sets and splitters in terms of Accuracy. Results are averaged across all the models and replicates.

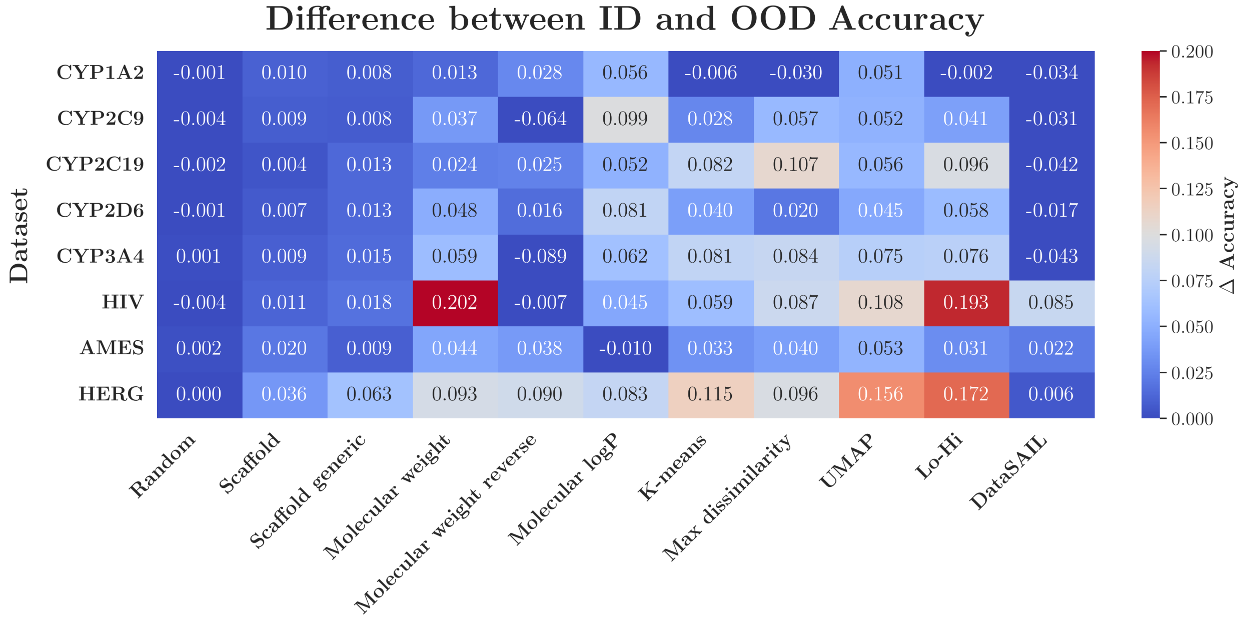

Figure S9: Drop in performance measured with accuracy for different data sets and splitters. The results are averaged over all the models and replicates.

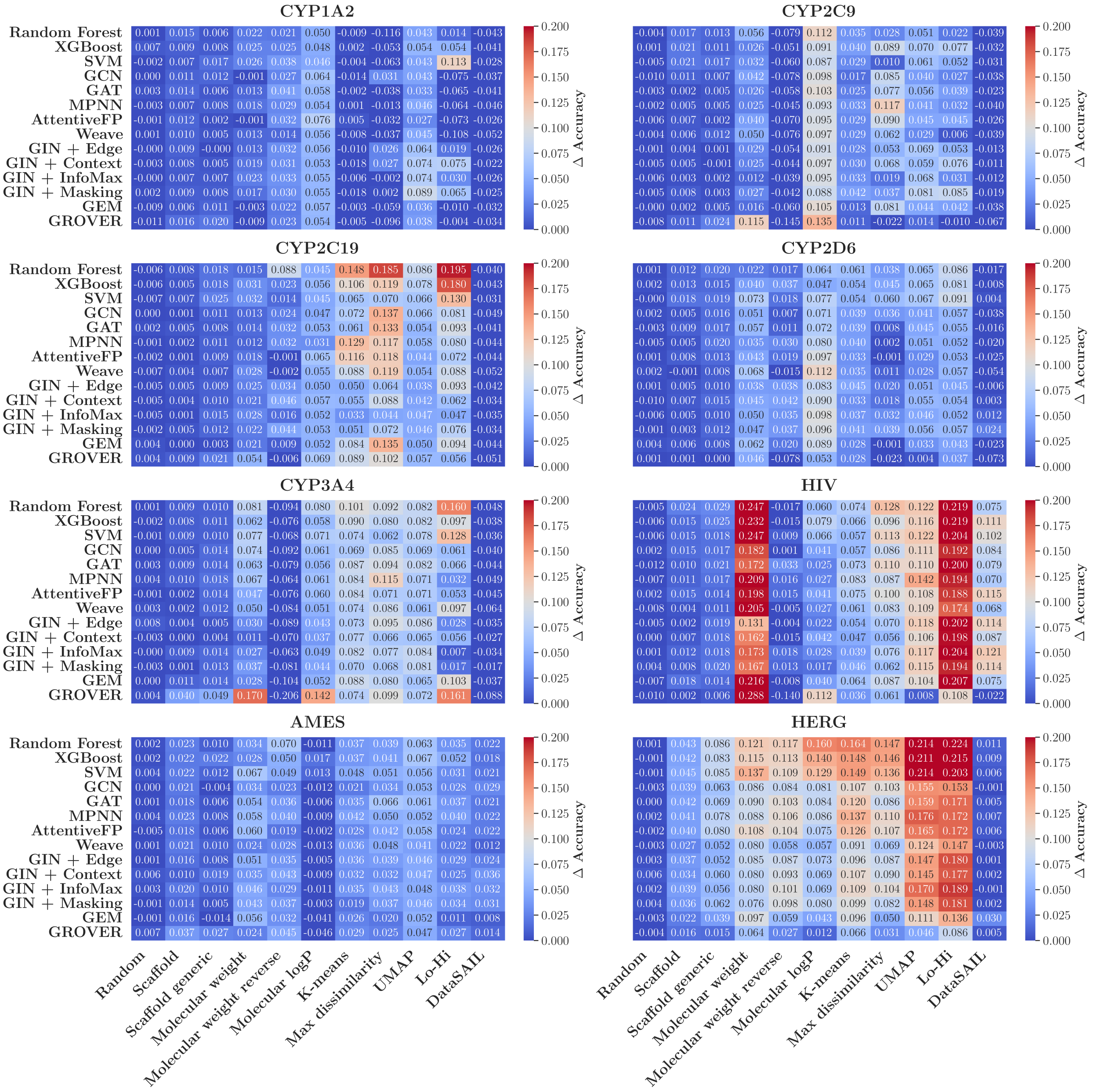

Figure S10: Drop in performance measured with accuracy across different data sets, splitters and models.

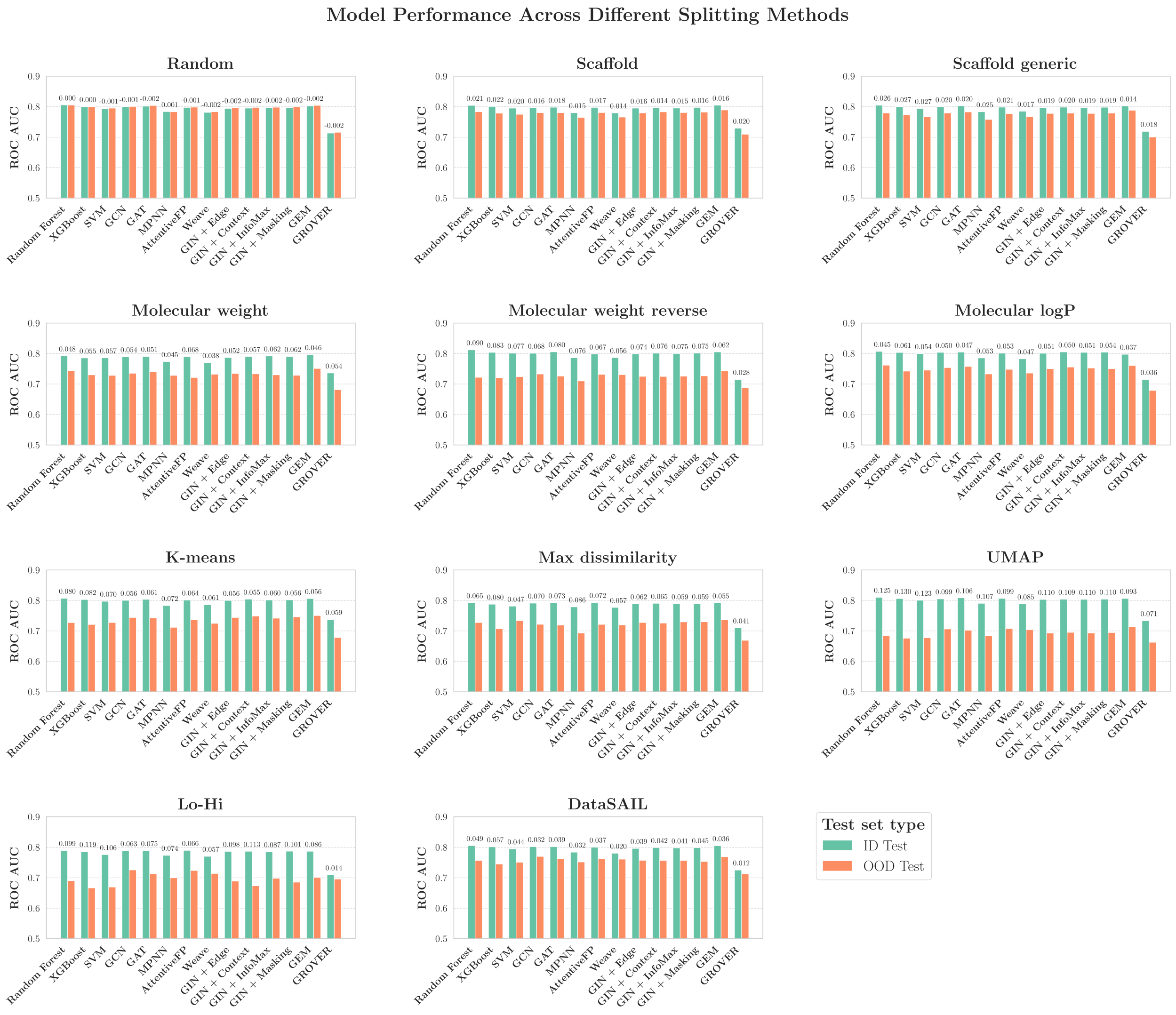

Figure S11: Performance measured with ROC-AUC for all the models. The results are averaged across all the data sets and folds.

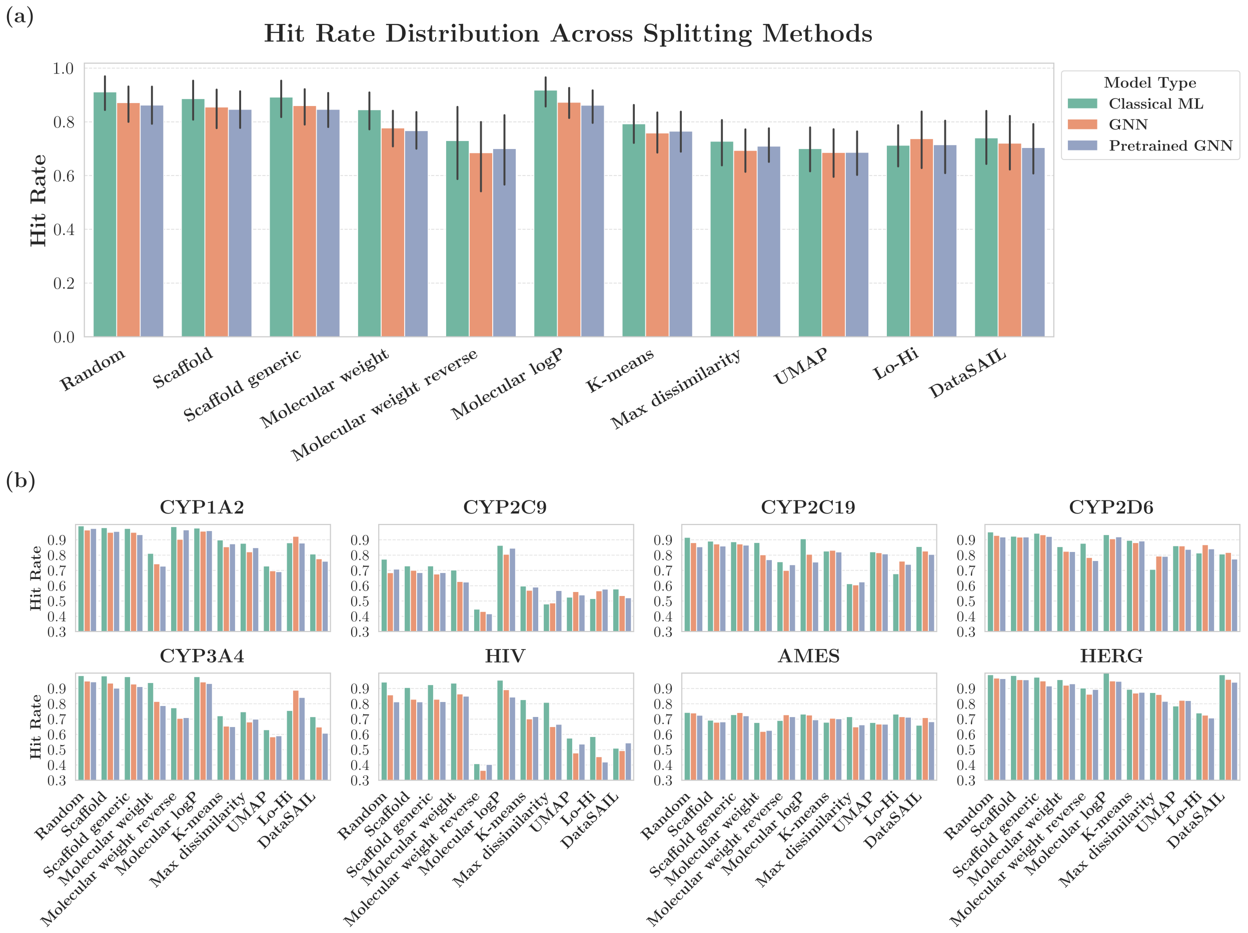

Figure S12: Top 100 hit rate performance on OOD test set generated with different splitting strategies. The figure presents both the aggregated results across all data sets and the results for each data set separately. Hit rate drop is measured relative to the random splitting performance.

6 Supplementary Tables

List of Supplementary Tables:

Table S1. Number of shared molecules between data sets.

Table S2. Hyperparameter configurations for all machine learning models used in this study, including graph neural networks and classical ML approaches.

Table S3. Canonical atom and bond features used for GNN models.

Table S4. Hyperparameters and configuration details for molecular data splitting strategies.

Table S5. Performance gap in terms of Accuracy between in-distribution and out-of-distribution test sets.

Table S6. Model-specific performance (Accuracy) gaps between in-distribution and out-of-distribution test sets for classical ML, GNN, and pretrained GNN models.

Table S7. Relationship between ID and OOD performance across data sets and splitters, showing slopes of linear fits and R-squared values for classical ML, GNN, and pretrained GNN models.

Table S1: Number of shared molecules between data sets.[1,2]

Data set	CYP1A2	CYP2C19	CYP2C9	CYP2D6	CYP3A4	HIV	AMES	HERG
CYP1A2	**12,535**	9,770	9,364	4,987	9,498	23	167	80
CYP2C19	9,770	**12,613**	9,659	5,097	9,621	32	175	103
CYP2C9	9,364	9,659	**12,033**	4,814	9,221	26	158	93
CYP2D6	4,987	5,097	4,814	**6,493**	4,947	15	83	63
CYP3A4	9,498	9,621	9,221	4,947	**12,278**	25	156	84
HIV	23	32	26	15	25	**4,980**	46	8
AMES	167	175	158	83	156	46	**7,157**	77
HERG	80	103	93	63	84	8	77	**12,890**

[1] Diagonal values (bold) represent the total number of molecules in each data set.
[2] Off-diagonal values represent the number of molecules shared between pairs of data sets.

Table S2: Hyperparameter configurations for all machine learning models used in this study, including graph neural networks and classical ML approaches.

Algorithm	Hyperparameters	Default Values
**Graph Neural Networks**		
GCN	Hidden feature size	256
	Predictive hidden size	128
	Number of GNN layers	2
	Atom featurization type	Canonical
	Residual connections	True
	Readout function	Weighted sum and max
	Total parameters	236,290
	Learning rate	2.00×10^{-2}
	Patience	30
	Batch size	128
	Dropout rate	0.05
	Max epochs	1000
GAT	Hidden feature size	64
	Predictive hidden size	128
	Number of attention heads	8
	Number of GNN layers	5
	Leaky ReLU slope	0.06
	Atom featurization type	Canonical
	Residual connections	True
	Readout function	Weighted sum and max
	Total parameters	1,148,994
	Learning rate	3.00×10^{-4}
	Patience	30
	Batch size	128
	Dropout rate	0.05
	Max epochs	1000
MPNN	Node output features	64
	Edge hidden features	128
	Message passing steps	6
	Set2Set steps	6
	Set2Set layers	3
	Atom featurization type	Canonical
	Bond featurization type	Canonical
	Total parameters	684,545
	Learning rate	3.00×10^{-4}
	Patience	30

Continued on next page

Table S2 – continued from previous page

Algorithm	Hyperparameters	Default Values
	Batch size	128
	Dropout rate	0.05
	Max epochs	1000
Weave	Hidden feature size	50
	Number of GNN layers	5
	Graph feature size	128
	Gaussian expansion	True
	Atom featurization type	Canonical
	Bond featurization type	Canonical
	Readout function	Weave Gather
	Total parameters	313,937
	Learning rate	3.00×10^{-4}
	Patience	30
	Batch size	128
	Dropout rate	0.05
	Max epochs	1000
AttentiveFP	Number of layers	2
	Number of timesteps	2
	Graph feature size	200
	Atom featurization type	Canonical
	Bond featurization type	Canonical
	Readout function	AttentiveFP Readout
	Total parameters	1,160,005
	Learning rate	3.00×10^{-4}
	Patience	30
	Batch size	128
	Dropout rate	0.0
	Max epochs	1000
GINs	Hidden feature size	300
	Number of GNN layers	5
	Jumping knowledge	Last
	Atom featurization type	Pretrain
	Bond featurization type	Pretrain
	Readout function	Sum
	Total parameters	1,858,201
	Learning rate	2.00×10^{-2}
	Patience	30

Continued on next page

Table S2 – continued from previous page

Algorithm	Hyperparameters	Default Values
	Batch size	128
	Dropout rate	0.05
	Max epochs	1000
GEM	Embedding dimension	32
	Predictive hidden size	128
	Number of GNN layers	8
	Number of MLP layers	2
	Atom featurization type	GEM Default
	Readout function	Mean
	Encoding learning rate	1.00×10^{-3}
	Head Learning rate	1.00×10^{-3}
	Batch size	32
	Dropout rate	0.2
	Max epochs	25
GROVER	MLP hidden size	200
	Number of MLP layers	2
	Distance cutoff	0.1
	Attention output size	128
	Hidden layer size	4
	Pretrained model	Grover base
	Initial learning rate	1.00×10^{-4}
	Max learning rate	1.00×10^{-3}
	Batch size	32
	Max epochs	30
**Classical Machine Learning**		
Random Forest	Number of estimators	[100, **200**, 300]
	Maximum depth	[10, 20, **40**]
	Minimum samples to split	[2, **4**, 8]
	Maximum features	$\sqrt{\text{features}}$
	Minimum samples per leaf	[**1**, 2, 4]
	Bootstrap samples	True
	Maximum sample size	None
SVM	Kernel function	RBF
	Kernel coefficient (γ)	[1, **scale**, auto]
	Regularization parameter (C)	[1, **10**, 100]
	Probability estimates	True

Continued on next page

Table S2 – continued from previous page

Algorithm	Hyperparameters	Default Values
XGBoost	Number of estimators	[100, **200**, 300]
	Maximum depth	[3, 6, **9**]
	Learning rate	[0.01, **0.1**, 0.3]
	Minimum loss reduction (γ)	[**0**, 1]
	L1 regularization (α)	[0, **1.0**]
	L2 regularization (λ)	[0, **1.0**]
	Minimum child weight	[**1.0**, 3.0]
	Column sampling ratio	0.5
	Row sampling ratio	1.0

Table S3: Canonical Atom and Bond features for GNN models.

Atom Features	Size	Description
Atom symbol	43	[C, N, O, S, F, Si, P, Cl, Br, Mg, Na, Ca, Fe, As, Al, I, B, V, K, Tl, Yb, Sb, Sn, Ag, Pd, Co, Se, Ti, Zn, H, Li, Ge, Cu, Au, Ni, Cd, In, Mn, Zr, Cr, Pt, Hg, Pb] (one-hot)
Degree	11	Number of covalent bonds [0, 1, 2, 3, 4, 5, 6, 7, 8, 9, 10] (one-hot)
Implicit hydrogens	7	Number of implicit hydrogen atoms; supported values include [0, 1, 2, 3, 4, 5, 6] (one-hot)
Formal charge	1	Formal charge of the atom
Radical electrons	1	Number of radical electrons of the atom
Hybridization	5	Supported hybridization states include [SP, SP2, SP3, SP3D, SP3D2] (one-hot)
Aromaticity	1	Whether the atom is aromatic
Total hydrogens	5	Total number of hydrogen atoms; supported values include [0, 1, 2, 3, 4] (one-hot)
**Bond Features**	**Size**	**Description**
Bond type	4	[single, double, triple, aromatic] (one-hot)
Conjugation	1	Whether the bond is conjugated
Ring membership	1	Whether the bond is in a ring of any size
Stereo configuration	6	Supported stereo configurations include [STEREONONE, STEREOANY, STEREOZ, STEREOE, STEREOCIS, STEREOTRANS] (one-hot)
Self-loop	1	Whether the bond is a self-loop or not

Table S4: Hyperparameters and configuration details for molecular data splitting strategies.[1,2,3]

Split Type	Hyperparameter	Value	Description
Random	–	–	Random assignment of molecules to train/test sets
Scaffold	make_generic	False	Uses specific molecular scaffolds (preserves functional groups and substituents)
Scaffold generic	make_generic	True	Uses generic molecular scaffolds (removes functional groups, keeps core structure)
Molecular weight	generalize_to_larger	True	Training set contains lighter molecules, test set contains heavier molecules
Molecular weight reverse	generalize_to_larger	False	Training set contains heavier molecules, test set contains lighter molecules
Molecular LogP	generalize_to_larger	True	Training set contains lower LogP molecules, test set contains higher LogP molecules
K-means clustering	n_clusters	10	Number of clusters for K-means algorithm
	metric	euclidean	Distance metric for clustering (After converting Morgan fingerprint to continuous representation)
Max dissimilarity	n_clusters	10	Number of clusters for dissimilarity-based split
	metric	euclidean	Distance metric for clustering (Maximum dissimilarity selection)
UMAP clustering	n_clusters	20	Number of clusters for UMAP clustering algorithm
	metric	jaccard	Distance metric for clustering (Morgan fingerprint-based clustering)
	n_neighbors	100	Controls how UMAP balances local-global structure in the data
	min_dist	0.1	Controls how tightly UMAP is allowed to pack points
	n_components	2	Dimensionality of the reduced dimension space
Lo-Hi	similarity_threshold	0.4	ECFP4 Tanimoto threshold
	max_min_gap	0.1	Determines when to halt optimization based on proximity to the optimal solution.
	coarsening_threshold	N/A	Whether to apply clustering
	train_min_frac	0.7	Minimum fraction for the training set
	test_min_frac	0.15	Minimum fraction for the test set
DataSAIL	metric	jaccard	Distance metric for clustering
	techniques	C1e	Cluster-based one-dimensional cold split
	solver	SCIP	Solver for solving the optimization problem

[1] All splitting strategies maintain the same ID data set/OOD test set ratios across data sets.
[2] For Lo-Hi splitting, the "Hi" split variant is used in this study.
[3] For DataSAIL splitting, the C1 (cluster-based one-dimensional cold split) method is employed.

Table S5: Performance gap in terms of accuracy between in-distribution (ID) and out-of-distribution (OOD) test sets.[1,2]

**Split Type**	**Metric**	**Data sets**							
		**CYP1A2**	**CYP2C9**	**CYP2C19**	**CYP2D6**	**CYP3A4**	**HIV**	**AMES**	**HERG**
Random	Test (ID)	0.69 (0.01)	0.72 (0.02)	0.78 (0.02)	0.78 (0.03)	0.69 (0.03)	0.80 (0.04)	0.63 (0.02)	0.79 (0.04)
	Test (OOD)	0.69 (0.01)	0.72 (0.02)	0.78 (0.02)	0.78 (0.03)	0.69 (0.03)	0.80 (0.03)	0.63 (0.02)	0.79 (0.04)
	Gap	-0.00 (0.02)	-0.00 (0.01)	-0.00 (0.02)	-0.00 (0.01)	0.00 (0.01)	-0.00 (0.02)	0.00 (0.02)	0.00 (0.01)
Scaffold	Test (ID)	0.69 (0.01)	0.72 (0.02)	0.78 (0.01)	0.79 (0.02)	0.69 (0.02)	0.80 (0.04)	0.63 (0.02)	0.79 (0.04)
	Test (OOD)	0.68 (0.02)	0.71 (0.02)	0.78 (0.02)	0.78 (0.02)	0.68 (0.03)	0.79 (0.03)	0.61 (0.03)	0.75 (0.04)
	Gap	0.01 (0.02)	0.01 (0.02)	0.00 (0.02)	0.01 (0.02)	0.01 (0.02)	0.01 (0.02)	0.02 (0.02)	0.04 (0.02)
Scaffold generic	Test (ID)	0.69 (0.01)	0.72 (0.02)	0.78 (0.02)	0.79 (0.03)	0.69 (0.02)	0.80 (0.04)	0.63 (0.03)	0.79 (0.05)
	Test (OOD)	0.68 (0.02)	0.71 (0.02)	0.77 (0.02)	0.77 (0.03)	0.68 (0.03)	0.78 (0.03)	0.62 (0.04)	0.73 (0.04)
	Gap	0.01 (0.02)	0.01 (0.02)	0.01 (0.01)	0.01 (0.02)	0.01 (0.02)	0.02 (0.02)	0.01 (0.03)	0.06 (0.03)
Molecular weight	Test (ID)	0.69 (0.02)	0.71 (0.01)	0.79 (0.01)	0.79 (0.02)	0.69 (0.03)	0.81 (0.03)	0.63 (0.02)	0.79 (0.04)
	Test (OOD)	0.68 (0.02)	0.68 (0.03)	0.76 (0.02)	0.74 (0.03)	0.63 (0.04)	0.60 (0.05)	0.58 (0.02)	0.70 (0.03)
	Gap	0.01 (0.02)	0.04 (0.03)	0.02 (0.01)	0.05 (0.02)	0.06 (0.04)	0.20 (0.06)	0.04 (0.02)	0.09 (0.02)
Molecular weight reverse	Test (ID)	0.69 (0.01)	0.71 (0.02)	0.78 (0.02)	0.79 (0.04)	0.67 (0.03)	0.79 (0.04)	0.63 (0.03)	0.79 (0.05)
	Test (OOD)	0.66 (0.01)	0.77 (0.02)	0.75 (0.03)	0.77 (0.02)	0.76 (0.03)	0.80 (0.03)	0.59 (0.03)	0.70 (0.03)
	Gap	0.03 (0.01)	-0.06 (0.03)	0.03 (0.03)	0.02 (0.04)	-0.09 (0.04)	-0.01 (0.04)	0.04 (0.02)	0.09 (0.03)
Molecular logp	Test (ID)	0.70 (0.01)	0.74 (0.01)	0.79 (0.02)	0.79 (0.02)	0.69 (0.02)	0.80 (0.04)	0.63 (0.02)	0.80 (0.04)
	Test (OOD)	0.65 (0.01)	0.64 (0.02)	0.74 (0.03)	0.71 (0.03)	0.63 (0.04)	0.76 (0.05)	0.64 (0.03)	0.72 (0.03)
	Gap	0.06 (0.01)	0.10 (0.02)	0.05 (0.02)	0.08 (0.02)	0.06 (0.03)	0.04 (0.03)	-0.01 (0.02)	0.08 (0.04)
K-means	Test (ID)	0.69 (0.02)	0.73 (0.02)	0.78 (0.02)	0.79 (0.02)	0.70 (0.03)	0.81 (0.03)	0.63 (0.03)	0.80 (0.04)
	Test (OOD)	0.69 (0.10)	0.70 (0.04)	0.70 (0.07)	0.75 (0.03)	0.62 (0.03)	0.75 (0.05)	0.59 (0.04)	0.68 (0.04)
	Gap	-0.01 (0.12)	0.03 (0.04)	0.08 (0.06)	0.04 (0.03)	0.08 (0.02)	0.06 (0.04)	0.03 (0.03)	0.12 (0.04)
Max dissimilarity	Test (ID)	0.66 (0.02)	0.71 (0.01)	0.77 (0.01)	0.78 (0.03)	0.67 (0.02)	0.80 (0.04)	0.64 (0.03)	0.79 (0.05)
	Test (OOD)	0.69 (0.10)	0.65 (0.06)	0.66 (0.09)	0.76 (0.04)	0.59 (0.06)	0.72 (0.04)	0.60 (0.03)	0.69 (0.05)
	Gap	-0.03 (0.09)	0.06 (0.06)	0.11 (0.10)	0.02 (0.04)	0.08 (0.06)	0.09 (0.04)	0.04 (0.03)	0.10 (0.05)
UMAP	Test (ID)	0.69 (0.03)	0.72 (0.02)	0.78 (0.02)	0.79 (0.03)	0.70 (0.02)	0.81 (0.04)	0.64 (0.02)	0.80 (0.04)
	Test (OOD)	0.64 (0.06)	0.67 (0.04)	0.73 (0.05)	0.74 (0.03)	0.62 (0.04)	0.70 (0.05)	0.59 (0.03)	0.64 (0.04)
	Gap	0.05 (0.08)	0.05 (0.05)	0.06 (0.04)	0.05 (0.03)	0.07 (0.04)	0.11 (0.06)	0.05 (0.03)	0.16 (0.06)
Lo-Hi	Test (ID)	0.64 (0.01)	0.72 (0.02)	0.78 (0.01)	0.80 (0.02)	0.67 (0.02)	0.82 (0.03)	0.62 (0.02)	0.79 (0.05)
	Test (OOD)	0.64 (0.10)	0.68 (0.04)	0.68 (0.06)	0.74 (0.03)	0.59 (0.07)	0.63 (0.01)	0.59 (0.02)	0.62 (0.03)
	Gap	-0.00 (0.10)	0.04 (0.04)	0.10 (0.05)	0.06 (0.02)	0.08 (0.07)	0.19 (0.03)	0.03 (0.02)	0.17 (0.04)
DataSAIL	Test (ID)	0.68 (0.01)	0.72 (0.02)	0.77 (0.02)	0.78 (0.03)	0.68 (0.03)	0.81 (0.05)	0.64 (0.02)	0.79 (0.05)
	Test (OOD)	0.71 (0.04)	0.75 (0.04)	0.82 (0.02)	0.80 (0.03)	0.72 (0.04)	0.72 (0.05)	0.62 (0.02)	0.78 (0.06)
	Gap	-0.03 (0.04)	-0.03 (0.05)	-0.04 (0.02)	-0.02 (0.04)	-0.04 (0.04)	0.09 (0.04)	0.02 (0.01)	0.01 (0.03)

[1] Performance is averaged over replications and models that are included in classical ML and GNN-based models.
[2] Parentheses show the standard deviations across models and replicates.

Table S6: Performance gap in terms of accuracy between in-distribution (ID) and out-of-distribution (OOD) test set

Split Type	Model Type	Metric	Data sets							
			CYP1A2	CYP2C9	CYP2C19	CYP2D6	CYP3A4	HIV	AMES	HERG
Random	Classical ML	Test (ID)	0.69 (0.01)	0.72 (0.01)	0.77 (0.01)	0.79 (0.01)	0.71 (0.01)	0.83 (0.01)	0.63 (0.02)	0.82 (0.01)
		Test (OOD)	0.69 (0.01)	0.73 (0.01)	0.78 (0.01)	0.79 (0.01)	0.71 (0.01)	0.84 (0.01)	0.62 (0.01)	0.82 (0.01)
		Gap	0.00 (0.02)	-0.00 (0.01)	-0.01 (0.01)	0.00 (0.02)	-0.00 (0.01)	-0.01 (0.02)	0.00 (0.02)	-0.00 (0.01)
	GNN	Test (ID)	0.69 (0.01)	0.72 (0.02)	0.78 (0.01)	0.79 (0.02)	0.69 (0.02)	0.80 (0.02)	0.64 (0.02)	0.79 (0.04)
		Test (OOD)	0.69 (0.01)	0.72 (0.01)	0.78 (0.01)	0.79 (0.02)	0.69 (0.02)	0.80 (0.02)	0.64 (0.02)	0.79 (0.03)
		Gap	-0.00 (0.01)	-0.00 (0.01)	-0.00 (0.02)	-0.00 (0.01)	0.00 (0.01)	-0.00 (0.02)	0.00 (0.02)	-0.00 (0.01)
	Pretrained GNN	Test (ID)	0.69 (0.01)	0.71 (0.02)	0.78 (0.02)	0.78 (0.04)	0.68 (0.03)	0.78 (0.04)	0.63 (0.02)	0.77 (0.04)
		Test (OOD)	0.69 (0.01)	0.72 (0.02)	0.78 (0.02)	0.78 (0.04)	0.68 (0.03)	0.79 (0.04)	0.63 (0.02)	0.77 (0.04)
		Gap	-0.00 (0.01)	-0.00 (0.01)	-0.00 (0.02)	-0.00 (0.01)	0.00 (0.01)	-0.00 (0.02)	0.00 (0.02)	0.00 (0.01)
Scaffold	Classical ML	Test (ID)	0.69 (0.01)	0.73 (0.01)	0.77 (0.01)	0.79 (0.01)	0.71 (0.01)	0.83 (0.01)	0.63 (0.02)	0.82 (0.01)
		Test (OOD)	0.68 (0.02)	0.71 (0.01)	0.77 (0.01)	0.78 (0.01)	0.70 (0.02)	0.81 (0.02)	0.61 (0.03)	0.78 (0.01)
		Gap	0.01 (0.02)	0.02 (0.02)	0.01 (0.02)	0.01 (0.02)	0.01 (0.02)	0.02 (0.02)	0.02 (0.02)	0.04 (0.01)
	GNN	Test (ID)	0.69 (0.01)	0.72 (0.01)	0.78 (0.01)	0.79 (0.02)	0.69 (0.02)	0.79 (0.02)	0.64 (0.02)	0.80 (0.03)
		Test (OOD)	0.68 (0.02)	0.71 (0.01)	0.78 (0.01)	0.78 (0.02)	0.68 (0.02)	0.78 (0.03)	0.62 (0.03)	0.76 (0.02)
		Gap	0.01 (0.02)	0.01 (0.01)	0.00 (0.02)	0.01 (0.02)	0.01 (0.02)	0.01 (0.02)	0.02 (0.02)	0.04 (0.02)
	Pretrained GNN	Test (ID)	0.69 (0.02)	0.71 (0.02)	0.78 (0.01)	0.78 (0.03)	0.68 (0.03)	0.79 (0.04)	0.63 (0.03)	0.77 (0.05)
		Test (OOD)	0.68 (0.02)	0.71 (0.02)	0.78 (0.02)	0.78 (0.03)	0.67 (0.04)	0.78 (0.04)	0.61 (0.04)	0.74 (0.05)
		Gap	0.01 (0.02)	0.01 (0.02)	0.00 (0.02)	0.00 (0.02)	0.01 (0.03)	0.01 (0.02)	0.02 (0.02)	0.03 (0.02)
Scaffold generic	Classical ML	Test (ID)	0.69 (0.01)	0.72 (0.01)	0.78 (0.01)	0.79 (0.01)	0.71 (0.01)	0.83 (0.01)	0.62 (0.02)	0.83 (0.01)
		Test (OOD)	0.68 (0.02)	0.71 (0.01)	0.76 (0.01)	0.78 (0.02)	0.70 (0.01)	0.81 (0.02)	0.61 (0.02)	0.74 (0.02)
		Gap	0.01 (0.02)	0.01 (0.02)	0.02 (0.02)	0.02 (0.02)	0.01 (0.02)	0.02 (0.02)	0.01 (0.03)	0.08 (0.02)
	GNN	Test (ID)	0.69 (0.01)	0.72 (0.02)	0.78 (0.01)	0.79 (0.02)	0.69 (0.01)	0.80 (0.02)	0.64 (0.02)	0.81 (0.02)
		Test (OOD)	0.68 (0.02)	0.71 (0.02)	0.77 (0.01)	0.78 (0.02)	0.68 (0.02)	0.78 (0.02)	0.63 (0.02)	0.74 (0.02)
		Gap	0.01 (0.02)	0.01 (0.01)	0.01 (0.01)	0.01 (0.02)	0.01 (0.02)	0.02 (0.02)	0.01 (0.03)	0.07 (0.02)
	Pretrained GNN	Test (ID)	0.69 (0.01)	0.72 (0.02)	0.78 (0.02)	0.78 (0.03)	0.68 (0.03)	0.79 (0.04)	0.62 (0.04)	0.77 (0.07)
		Test (OOD)	0.68 (0.02)	0.71 (0.03)	0.77 (0.03)	0.77 (0.03)	0.67 (0.04)	0.77 (0.04)	0.61 (0.06)	0.72 (0.06)
		Gap	0.01 (0.02)	0.01 (0.02)	0.01 (0.01)	0.01 (0.02)	0.02 (0.02)	0.02 (0.03)	0.01 (0.03)	0.05 (0.03)
Molecular weight	Classical ML	Test (ID)	0.69 (0.00)	0.72 (0.00)	0.78 (0.00)	0.79 (0.00)	0.71 (0.00)	0.83 (0.00)	0.62 (0.01)	0.82 (0.01)
		Test (OOD)	0.66 (0.01)	0.68 (0.01)	0.75 (0.01)	0.74 (0.02)	0.64 (0.01)	0.59 (0.01)	0.58 (0.02)	0.70 (0.00)
		Gap	0.02 (0.00)	0.04 (0.01)	0.03 (0.01)	0.05 (0.02)	0.07 (0.01)	0.24 (0.01)	0.04 (0.02)	0.12 (0.01)
	GNN	Test (ID)	0.69 (0.01)	0.72 (0.01)	0.79 (0.01)	0.79 (0.02)	0.70 (0.01)	0.80 (0.03)	0.63 (0.02)	0.80 (0.02)
		Test (OOD)	0.68 (0.01)	0.68 (0.02)	0.78 (0.01)	0.74 (0.03)	0.64 (0.02)	0.60 (0.05)	0.59 (0.02)	0.71 (0.02)
		Gap	0.01 (0.02)	0.04 (0.03)	0.02 (0.01)	0.05 (0.02)	0.06 (0.03)	0.19 (0.06)	0.05 (0.02)	0.09 (0.01)
	Pretrained GNN	Test (ID)	0.69 (0.03)	0.71 (0.01)	0.79 (0.01)	0.79 (0.03)	0.67 (0.03)	0.80 (0.02)	0.63 (0.02)	0.77 (0.05)
		Test (OOD)	0.68 (0.02)	0.67 (0.05)	0.76 (0.03)	0.74 (0.03)	0.62 (0.06)	0.61 (0.06)	0.58 (0.02)	0.69 (0.04)
		Gap	0.01 (0.03)	0.04 (0.04)	0.03 (0.02)	0.05 (0.02)	0.05 (0.06)	0.19 (0.06)	0.04 (0.02)	0.08 (0.02)
Molecular weight reverse	Classical ML	Test (ID)	0.69 (0.01)	0.72 (0.00)	0.77 (0.00)	0.81 (0.00)	0.69 (0.01)	0.82 (0.00)	0.64 (0.01)	0.83 (0.01)
		Test (OOD)	0.67 (0.01)	0.78 (0.01)	0.73 (0.03)	0.78 (0.01)	0.77 (0.01)	0.83 (0.01)	0.59 (0.00)	0.71 (0.01)
		Gap	0.03 (0.01)	-0.06 (0.01)	0.04 (0.03)	0.02 (0.01)	-0.08 (0.01)	-0.01 (0.01)	0.06 (0.01)	0.11 (0.00)
	GNN	Test (ID)	0.69 (0.01)	0.71 (0.01)	0.79 (0.01)	0.79 (0.02)	0.67 (0.01)	0.80 (0.01)	0.63 (0.01)	0.80 (0.02)
		Test (OOD)	0.66 (0.01)	0.77 (0.02)	0.77 (0.02)	0.78 (0.02)	0.75 (0.03)	0.79 (0.02)	0.60 (0.03)	0.71 (0.02)
		Gap	0.03 (0.01)	-0.07 (0.02)	0.02 (0.02)	0.01 (0.03)	-0.08 (0.02)	0.01 (0.02)	0.03 (0.02)	0.09 (0.02)

Continued on next page

Table S6 – continued from previous page

Split Type	Model Type	Metric	Data sets							
			CYP1A2	CYP2C9	CYP2C19	CYP2D6	CYP3A4	HIV	AMES	HERG
	Pretrained GNN	Test (ID)	0.69 (0.01)	0.70 (0.03)	0.78 (0.03)	0.78 (0.05)	0.65 (0.04)	0.77 (0.04)	0.63 (0.04)	0.77 (0.06)
		Test (OOD)	0.66 (0.01)	0.77 (0.02)	0.75 (0.02)	0.77 (0.02)	0.75 (0.03)	0.79 (0.04)	0.59 (0.03)	0.69 (0.04)
		Gap	0.03 (0.01)	-0.06 (0.04)	0.02 (0.03)	0.02 (0.05)	-0.10 (0.05)	-0.02 (0.06)	0.04 (0.03)	0.08 (0.03)
Molecular logp	Classical ML	Test (ID)	0.70 (0.01)	0.74 (0.00)	0.79 (0.00)	0.79 (0.01)	0.70 (0.01)	0.83 (0.00)	0.62 (0.01)	0.84 (0.01)
		Test (OOD)	0.65 (0.01)	0.64 (0.01)	0.74 (0.01)	0.73 (0.01)	0.63 (0.00)	0.77 (0.01)	0.62 (0.02)	0.69 (0.02)
		Gap	0.05 (0.00)	0.10 (0.01)	0.05 (0.01)	0.06 (0.01)	0.07 (0.01)	0.07 (0.01)	0.01 (0.01)	0.14 (0.01)
	GNN	Test (ID)	0.71 (0.01)	0.74 (0.01)	0.80 (0.01)	0.79 (0.02)	0.69 (0.02)	0.78 (0.04)	0.64 (0.01)	0.81 (0.03)
		Test (OOD)	0.65 (0.01)	0.64 (0.01)	0.75 (0.01)	0.71 (0.04)	0.64 (0.01)	0.75 (0.04)	0.65 (0.01)	0.73 (0.02)
		Gap	0.06 (0.01)	0.10 (0.01)	0.05 (0.02)	0.09 (0.03)	0.06 (0.01)	0.03 (0.03)	-0.01 (0.01)	0.08 (0.02)
	Pretrained GNN	Test (ID)	0.70 (0.01)	0.74 (0.02)	0.79 (0.03)	0.79 (0.03)	0.67 (0.03)	0.80 (0.03)	0.62 (0.03)	0.78 (0.05)
		Test (OOD)	0.65 (0.01)	0.64 (0.03)	0.74 (0.04)	0.70 (0.03)	0.61 (0.06)	0.75 (0.07)	0.64 (0.03)	0.72 (0.03)
		Gap	0.05 (0.01)	0.10 (0.02)	0.06 (0.03)	0.08 (0.02)	0.06 (0.04)	0.04 (0.04)	-0.02 (0.03)	0.06 (0.03)
K-means	Classical ML	Test (ID)	0.68 (0.02)	0.73 (0.01)	0.77 (0.01)	0.80 (0.01)	0.71 (0.02)	0.84 (0.01)	0.62 (0.01)	0.83 (0.01)
		Test (OOD)	0.69 (0.11)	0.70 (0.03)	0.67 (0.07)	0.74 (0.02)	0.63 (0.02)	0.77 (0.02)	0.58 (0.03)	0.67 (0.04)
		Gap	-0.00 (0.13)	0.03 (0.04)	0.11 (0.07)	0.06 (0.03)	0.09 (0.02)	0.07 (0.02)	0.04 (0.04)	0.15 (0.04)
	GNN	Test (ID)	0.69 (0.02)	0.72 (0.01)	0.78 (0.01)	0.79 (0.02)	0.70 (0.02)	0.80 (0.03)	0.63 (0.02)	0.81 (0.03)
		Test (OOD)	0.69 (0.09)	0.70 (0.04)	0.69 (0.07)	0.75 (0.03)	0.62 (0.03)	0.73 (0.05)	0.60 (0.03)	0.69 (0.03)
		Gap	-0.00 (0.11)	0.03 (0.05)	0.09 (0.07)	0.04 (0.04)	0.08 (0.02)	0.07 (0.05)	0.03 (0.03)	0.12 (0.03)
	Pretrained GNN	Test (ID)	0.69 (0.03)	0.72 (0.02)	0.78 (0.03)	0.79 (0.03)	0.69 (0.03)	0.80 (0.04)	0.62 (0.03)	0.78 (0.05)
		Test (OOD)	0.70 (0.10)	0.70 (0.03)	0.72 (0.06)	0.75 (0.03)	0.61 (0.03)	0.75 (0.05)	0.60 (0.04)	0.68 (0.04)
		Gap	-0.01 (0.12)	0.03 (0.04)	0.06 (0.05)	0.04 (0.03)	0.08 (0.03)	0.05 (0.05)	0.03 (0.03)	0.10 (0.03)
Max dissimilarity	Classical ML	Test (ID)	0.66 (0.01)	0.70 (0.01)	0.76 (0.01)	0.79 (0.01)	0.69 (0.01)	0.84 (0.01)	0.64 (0.02)	0.82 (0.01)
		Test (OOD)	0.74 (0.07)	0.66 (0.05)	0.63 (0.09)	0.74 (0.02)	0.61 (0.05)	0.72 (0.04)	0.59 (0.02)	0.68 (0.05)
		Gap	-0.08 (0.07)	0.04 (0.05)	0.12 (0.09)	0.05 (0.02)	0.08 (0.06)	0.11 (0.03)	0.04 (0.02)	0.14 (0.05)
	GNN	Test (ID)	0.67 (0.01)	0.71 (0.01)	0.78 (0.01)	0.78 (0.02)	0.68 (0.02)	0.80 (0.03)	0.65 (0.01)	0.80 (0.03)
		Test (OOD)	0.69 (0.09)	0.62 (0.06)	0.65 (0.10)	0.77 (0.03)	0.59 (0.06)	0.71 (0.03)	0.60 (0.02)	0.70 (0.04)
		Gap	-0.02 (0.08)	0.09 (0.06)	0.12 (0.11)	0.01 (0.03)	0.09 (0.06)	0.09 (0.04)	0.05 (0.02)	0.09 (0.04)
	Pretrained GNN	Test (ID)	0.66 (0.02)	0.71 (0.02)	0.77 (0.01)	0.77 (0.04)	0.66 (0.03)	0.79 (0.04)	0.64 (0.03)	0.77 (0.05)
		Test (OOD)	0.68 (0.11)	0.67 (0.07)	0.69 (0.08)	0.75 (0.05)	0.58 (0.07)	0.72 (0.05)	0.60 (0.03)	0.69 (0.06)
		Gap	-0.02 (0.11)	0.04 (0.07)	0.08 (0.08)	0.01 (0.04)	0.08 (0.07)	0.07 (0.05)	0.03 (0.03)	0.07 (0.05)
UMAP	Classical ML	Test (ID)	0.68 (0.03)	0.72 (0.02)	0.78 (0.01)	0.80 (0.01)	0.71 (0.01)	0.84 (0.01)	0.64 (0.02)	0.83 (0.01)
		Test (OOD)	0.64 (0.06)	0.66 (0.04)	0.70 (0.04)	0.73 (0.02)	0.63 (0.04)	0.72 (0.04)	0.57 (0.02)	0.62 (0.03)
		Gap	0.05 (0.09)	0.06 (0.04)	0.08 (0.04)	0.07 (0.02)	0.08 (0.04)	0.12 (0.04)	0.06 (0.03)	0.21 (0.03)
	GNN	Test (ID)	0.69 (0.03)	0.72 (0.02)	0.79 (0.02)	0.79 (0.02)	0.70 (0.02)	0.82 (0.02)	0.65 (0.02)	0.80 (0.03)
		Test (OOD)	0.65 (0.06)	0.68 (0.04)	0.73 (0.05)	0.75 (0.02)	0.63 (0.03)	0.70 (0.05)	0.59 (0.03)	0.65 (0.03)
		Gap	0.04 (0.09)	0.04 (0.05)	0.06 (0.05)	0.04 (0.02)	0.07 (0.04)	0.12 (0.05)	0.05 (0.03)	0.16 (0.04)
	Pretrained GNN	Test (ID)	0.69 (0.03)	0.72 (0.02)	0.78 (0.02)	0.78 (0.04)	0.69 (0.02)	0.79 (0.04)	0.64 (0.03)	0.77 (0.05)
		Test (OOD)	0.63 (0.07)	0.66 (0.05)	0.74 (0.04)	0.74 (0.04)	0.61 (0.04)	0.70 (0.05)	0.59 (0.03)	0.65 (0.04)
		Gap	0.06 (0.08)	0.06 (0.05)	0.05 (0.04)	0.04 (0.03)	0.08 (0.04)	0.09 (0.07)	0.05 (0.03)	0.13 (0.06)
Lo-Hi	Classical ML	Test (ID)	0.63 (0.01)	0.71 (0.01)	0.77 (0.00)	0.80 (0.00)	0.68 (0.01)	0.84 (0.01)	0.62 (0.01)	0.83 (0.00)
		Test (OOD)	0.57 (0.05)	0.66 (0.02)	0.60 (0.03)	0.71 (0.01)	0.55 (0.02)	0.63 (0.00)	0.59 (0.01)	0.61 (0.01)
		Gap	0.06 (0.04)	0.05 (0.02)	0.17 (0.03)	0.09 (0.01)	0.13 (0.03)	0.21 (0.01)	0.04 (0.01)	0.21 (0.01)

Continued on next page

Table S6 – continued from previous page

Split Type	Model Type	Metric	Data sets							
			CYP1A2	CYP2C9	CYP2C19	CYP2D6	CYP3A4	HIV	AMES	HERG
	GNN	Test (ID)	0.65 (0.01)	0.72 (0.01)	0.79 (0.01)	0.80 (0.02)	0.67 (0.02)	0.82 (0.02)	0.63 (0.02)	0.80 (0.02)
		Test (OOD)	0.72 (0.06)	0.69 (0.03)	0.70 (0.03)	0.75 (0.02)	0.61 (0.05)	0.63 (0.01)	0.60 (0.02)	0.64 (0.02)
		Gap	-0.08 (0.05)	0.03 (0.03)	0.08 (0.03)	0.05 (0.01)	0.06 (0.05)	0.19 (0.02)	0.03 (0.01)	0.16 (0.02)
	Pretrained GNN	Test (ID)	0.64 (0.02)	0.72 (0.02)	0.78 (0.02)	0.79 (0.02)	0.66 (0.02)	0.81 (0.04)	0.62 (0.02)	0.77 (0.06)
		Test (OOD)	0.61 (0.11)	0.68 (0.05)	0.71 (0.04)	0.75 (0.03)	0.60 (0.08)	0.63 (0.01)	0.60 (0.03)	0.61 (0.03)
		Gap	0.03 (0.10)	0.05 (0.05)	0.07 (0.04)	0.05 (0.02)	0.06 (0.08)	0.19 (0.04)	0.03 (0.02)	0.16 (0.05)
DataSAIL	Classical ML	Test (ID)	0.67 (0.01)	0.73 (0.01)	0.77 (0.01)	0.79 (0.01)	0.70 (0.01)	0.84 (0.01)	0.63 (0.02)	0.82 (0.01)
		Test (OOD)	0.71 (0.03)	0.76 (0.04)	0.81 (0.02)	0.80 (0.02)	0.74 (0.03)	0.75 (0.02)	0.61 (0.02)	0.81 (0.01)
		Gap	-0.04 (0.04)	-0.03 (0.04)	-0.04 (0.02)	-0.01 (0.03)	-0.04 (0.04)	0.10 (0.02)	0.02 (0.00)	0.01 (0.01)
	GNN	Test (ID)	0.68 (0.01)	0.72 (0.01)	0.78 (0.01)	0.78 (0.03)	0.68 (0.02)	0.81 (0.03)	0.65 (0.02)	0.80 (0.03)
		Test (OOD)	0.72 (0.04)	0.75 (0.04)	0.82 (0.02)	0.81 (0.03)	0.73 (0.03)	0.73 (0.03)	0.63 (0.02)	0.80 (0.03)
		Gap	-0.04 (0.04)	-0.03 (0.05)	-0.05 (0.02)	-0.03 (0.03)	-0.05 (0.04)	0.08 (0.03)	0.02 (0.01)	0.00 (0.01)
	Pretrained GNN	Test (ID)	0.68 (0.01)	0.71 (0.03)	0.78 (0.02)	0.78 (0.04)	0.66 (0.03)	0.78 (0.06)	0.64 (0.02)	0.76 (0.07)
		Test (OOD)	0.70 (0.04)	0.74 (0.04)	0.82 (0.03)	0.79 (0.04)	0.70 (0.03)	0.70 (0.06)	0.62 (0.02)	0.75 (0.08)
		Gap	-0.03 (0.04)	-0.03 (0.05)	-0.04 (0.03)	-0.01 (0.04)	-0.04 (0.04)	0.08 (0.06)	0.02 (0.02)	0.01 (0.04)

Table S7: Relationship between ID and OOD performance across data sets and splitters.

Split Type	Model Type	Metric	Data sets							
			**CYP1A2**	**CYP2C9**	**CYP2C19**	**CYP2D6**	**CYP3A4**	**HIV**	**AMES**	**HERG**
Random	Classical ML	Accuracy	-0.17 (0.02)	-0.28 (0.11)	-0.31 (0.11)	-0.20 (0.06)	0.20 (0.03)	-0.67 (0.40)	0.48 (0.25)	0.29 (0.12)
		ROC-AUC	0.26 (0.04)	0.25 (0.17)	-0.21 (0.07)	0.04 (0.00)	0.49 (0.24)	-0.16 (0.02)	0.34 (0.15)	0.38 (0.19)
	GNN	Accuracy	0.27 (0.12)	0.45 (0.32)	0.35 (0.12)	0.64 (0.54)	0.77 (0.61)	0.73 (0.46)	0.48 (0.27)	0.88 (0.85)
		ROC-AUC	0.08 (0.01)	0.41 (0.24)	-0.00 (0.00)	0.67 (0.48)	0.68 (0.38)	0.33 (0.08)	0.12 (0.03)	0.93 (0.83)
	Pretrained GNN	Accuracy	0.18 (0.06)	0.80 (0.77)	0.71 (0.59)	1.01 (0.90)	0.90 (0.83)	0.83 (0.79)	0.80 (0.62)	0.93 (0.95)
		ROC-AUC	0.21 (0.09)	0.69 (0.65)	0.75 (0.49)	0.91 (0.96)	0.91 (0.78)	1.01 (0.93)	0.80 (0.65)	0.92 (0.97)
Scaffold	Classical ML	Accuracy	-0.32 (0.05)	0.28 (0.02)	-0.47 (0.09)	-0.09 (0.01)	0.08 (0.00)	-0.18 (0.01)	0.66 (0.19)	0.41 (0.15)
		ROC-AUC	0.05 (0.00)	0.72 (0.21)	-0.96 (0.15)	-0.23 (0.05)	-0.18 (0.01)	-0.43 (0.15)	0.70 (0.29)	0.38 (0.12)
	GNN	Accuracy	0.04 (0.00)	0.64 (0.21)	0.24 (0.06)	0.38 (0.18)	0.32 (0.07)	0.70 (0.40)	0.68 (0.21)	0.66 (0.68)
		ROC-AUC	-0.37 (0.04)	0.55 (0.25)	-0.18 (0.03)	0.43 (0.22)	0.24 (0.04)	0.13 (0.03)	-0.15 (0.02)	0.74 (0.67)
	Pretrained GNN	Accuracy	0.27 (0.04)	0.96 (0.62)	0.77 (0.34)	0.79 (0.61)	0.93 (0.44)	0.72 (0.64)	1.13 (0.71)	0.84 (0.87)
		ROC-AUC	-0.11 (0.00)	1.37 (0.76)	0.58 (0.22)	0.58 (0.48)	1.19 (0.59)	0.84 (0.82)	0.71 (0.33)	0.78 (0.88)
Scaffold generic	Classical ML	Accuracy	0.28 (0.04)	0.11 (0.00)	-0.35 (0.19)	-0.32 (0.06)	-0.11 (0.01)	-0.07 (0.00)	0.07 (0.00)	0.68 (0.13)
		ROC-AUC	0.43 (0.06)	0.95 (0.60)	-0.27 (0.11)	-0.52 (0.17)	0.22 (0.02)	-0.33 (0.04)	0.56 (0.22)	0.45 (0.05)
	GNN	Accuracy	0.47 (0.09)	1.04 (0.58)	0.42 (0.20)	0.45 (0.11)	0.56 (0.17)	0.33 (0.12)	-0.18 (0.02)	0.57 (0.30)
		ROC-AUC	-0.11 (0.00)	0.62 (0.35)	0.12 (0.02)	0.36 (0.07)	0.29 (0.04)	0.26 (0.05)	-0.03 (0.00)	0.85 (0.36)
	Pretrained GNN	Accuracy	0.25 (0.04)	1.06 (0.59)	0.95 (0.73)	0.79 (0.61)	1.07 (0.60)	0.74 (0.65)	1.19 (0.68)	0.78 (0.87)
		ROC-AUC	0.19 (0.01)	1.38 (0.76)	0.72 (0.54)	0.68 (0.45)	1.35 (0.61)	0.84 (0.82)	0.66 (0.62)	0.76 (0.89)
Molecular weight	Classical ML	Accuracy	0.94 (0.49)	0.87 (0.07)	1.34 (0.34)	3.51 (0.43)	-1.07 (0.57)	-0.43 (0.09)	0.94 (0.12)	0.02 (0.01)
		ROC-AUC	0.98 (0.90)	0.27 (0.39)	1.53 (0.43)	-1.26 (0.55)	1.16 (0.58)	1.12 (0.11)	1.28 (0.94)	0.05 (0.01)
	GNN	Accuracy	-0.01 (0.00)	0.47 (0.03)	0.88 (0.33)	1.28 (0.65)	0.18 (0.01)	-0.32 (0.03)	0.40 (0.25)	0.60 (0.65)
		ROC-AUC	0.64 (0.22)	-0.09 (0.01)	0.61 (0.30)	0.32 (0.10)	0.38 (0.10)	-0.93 (0.16)	0.05 (0.00)	0.80 (0.53)
	Pretrained GNN	Accuracy	0.13 (0.03)	1.60 (0.23)	1.60 (0.71)	0.61 (0.54)	1.01 (0.16)	1.05 (0.12)	0.47 (0.33)	0.84 (0.90)
		ROC-AUC	1.25 (0.56)	1.29 (0.67)	0.83 (0.76)	1.58 (0.84)	1.18 (0.63)	0.59 (0.25)	0.48 (0.16)	0.74 (0.85)
Molecular weight reverse	Classical ML	Accuracy	2.10 (0.90)	-1.19 (0.51)	-5.14 (0.32)	-1.24 (0.37)	0.85 (0.24)	-1.28 (0.63)	0.05 (0.02)	1.27 (0.84)
		ROC-AUC	1.75 (0.98)	0.99 (0.91)	0.62 (0.62)	-0.66 (0.47)	0.91 (0.64)	0.74 (0.04)	0.04 (0.01)	1.85 (0.68)
	GNN	Accuracy	0.02 (0.00)	0.58 (0.15)	-0.04 (0.00)	0.20 (0.05)	1.25 (0.49)	0.59 (0.13)	1.33 (0.43)	0.22 (0.08)
		ROC-AUC	0.40 (0.05)	0.16 (0.02)	0.26 (0.04)	0.82 (0.48)	0.58 (0.34)	0.65 (0.18)	1.47 (0.19)	0.18 (0.03)
	Pretrained GNN	Accuracy	0.49 (0.22)	-0.30 (0.13)	0.21 (0.07)	0.13 (0.06)	-0.08 (0.01)	-0.01 (0.00)	0.57 (0.45)	0.57 (0.79)
		ROC-AUC	1.07 (0.41)	0.59 (0.59)	0.39 (0.39)	0.27 (0.44)	0.41 (0.57)	0.51 (0.82)	1.47 (0.79)	0.53 (0.85)
Molecular logp	Classical ML	Accuracy	0.64 (0.66)	1.32 (0.18)	0.34 (0.04)	-0.08 (0.00)	-0.28 (0.20)	-0.47 (0.05)	1.44 (0.48)	1.34 (0.33)
		ROC-AUC	0.61 (0.93)	0.90 (0.84)	0.64 (0.02)	-1.43 (0.52)	1.38 (0.94)	-1.29 (0.22)	0.91 (0.51)	1.35 (0.93)
	GNN	Accuracy	0.21 (0.05)	1.01 (0.41)	0.10 (0.01)	1.54 (0.54)	0.51 (0.32)	0.93 (0.60)	0.29 (0.09)	0.51 (0.59)
		ROC-AUC	0.57 (0.17)	0.51 (0.32)	0.03 (0.00)	1.29 (0.60)	0.98 (0.38)	0.77 (0.68)	0.37 (0.10)	1.28 (0.62)
	Pretrained GNN	Accuracy	0.37 (0.17)	1.55 (0.74)	1.07 (0.49)	0.79 (0.54)	1.80 (0.73)	1.76 (0.80)	0.66 (0.46)	0.55 (0.79)
		ROC-AUC	0.79 (0.52)	0.70 (0.75)	0.26 (0.18)	0.80 (0.79)	1.64 (0.89)	1.11 (0.96)	0.86 (0.43)	0.36 (0.53)
K-means	Classical ML	Accuracy	-4.17 (0.78)	-1.56 (0.37)	0.09 (0.00)	-0.74 (0.17)	0.61 (0.24)	-0.07 (0.00)	0.14 (0.00)	-1.24 (0.10)
		ROC-AUC	-3.18 (0.78)	-0.38 (0.02)	2.45 (0.10)	-0.14 (0.01)	-0.38 (0.05)	-1.22 (0.20)	-0.01 (0.00)	-2.31 (0.14)
	GNN	Accuracy	-3.54 (0.77)	-0.99 (0.13)	0.98 (0.03)	0.21 (0.01)	0.85 (0.28)	0.85 (0.21)	0.78 (0.20)	0.22 (0.04)
		ROC-AUC	-3.31 (0.84)	-0.88 (0.11)	2.68 (0.22)	0.54 (0.08)	0.50 (0.15)	-0.19 (0.01)	0.16 (0.00)	0.29 (0.02)
	Pretrained GNN	Accuracy	-2.91 (0.59)	0.01 (0.00)	0.93 (0.22)	0.65 (0.31)	0.68 (0.40)	0.54 (0.15)	0.78 (0.41)	0.60 (0.53)
		ROC-AUC	-3.30 (0.81)	0.17 (0.01)	2.26 (0.41)	0.64 (0.24)	0.78 (0.35)	0.61 (0.28)	0.72 (0.21)	0.45 (0.31)
Max dissimilarity	Classical ML	Accuracy	0.67 (0.02)	1.63 (0.06)	-0.38 (0.00)	0.32 (0.04)	-3.19 (0.34)	1.45 (0.13)	0.67 (0.27)	0.54 (0.01)
		ROC-AUC	0.26 (0.00)	-1.70 (0.11)	0.39 (0.00)	0.38 (0.02)	-2.37 (0.24)	0.26 (0.01)	0.39 (0.21)	0.90 (0.01)
	GNN	Accuracy	1.86 (0.07)	1.30 (0.07)	-2.94 (0.11)	0.79 (0.32)	-1.31 (0.12)	0.08 (0.00)	0.48 (0.10)	0.38 (0.11)
		ROC-AUC	-0.67 (0.01)	1.93 (0.14)	-0.80 (0.01)	0.03 (0.00)	-1.24 (0.08)	0.71 (0.05)	0.69 (0.20)	0.45 (0.08)

Continued on next page

Table S7 – continued from previous page

Split Type	Model Type	Metric	Data sets							
			CYP1A2	CYP2C9	CYP2C19	CYP2D6	CYP3A4	HIV	AMES	HERG
	Pretrained GNN	Accuracy	1.57 (0.08)	0.11 (0.00)	0.25 (0.00)	0.65 (0.33)	0.38 (0.03)	0.64 (0.28)	0.49 (0.35)	0.62 (0.37)
		ROC-AUC	-2.09 (0.11)	1.41 (0.16)	1.00 (0.06)	0.77 (0.30)	-0.00 (0.00)	0.80 (0.53)	0.48 (0.34)	0.27 (0.06)
UMAP	Classical ML	Accuracy	-1.62 (0.67)	-0.66 (0.09)	-0.44 (0.01)	-0.17 (0.01)	-0.36 (0.02)	0.71 (0.02)	0.10 (0.01)	-0.04 (0.00)
		ROC-AUC	-1.69 (0.66)	0.70 (0.15)	-0.72 (0.01)	-1.00 (0.26)	-0.44 (0.05)	-1.11 (0.28)	0.60 (0.21)	-0.94 (0.05)
	GNN	Accuracy	-1.79 (0.54)	-0.92 (0.18)	0.53 (0.03)	0.48 (0.11)	-0.25 (0.01)	0.46 (0.02)	0.64 (0.21)	0.04 (0.00)
		ROC-AUC	-2.18 (0.64)	0.34 (0.01)	-0.71 (0.04)	-0.10 (0.01)	-0.36 (0.04)	0.04 (0.00)	0.16 (0.01)	-0.08 (0.00)
	Pretrained GNN	Accuracy	-1.07 (0.21)	-0.30 (0.02)	0.73 (0.12)	0.67 (0.52)	0.41 (0.06)	0.08 (0.00)	0.46 (0.19)	0.13 (0.03)
		ROC-AUC	-1.77 (0.45)	1.07 (0.22)	0.80 (0.15)	0.50 (0.38)	0.03 (0.00)	0.46 (0.16)	0.54 (0.10)	-0.05 (0.00)
Lo-Hi	Classical ML	Accuracy	5.32 (0.75)	-0.37 (0.04)	-5.04 (0.25)	1.50 (0.64)	-0.65 (0.04)	0.19 (0.75)	0.50 (0.44)	1.03 (0.12)
		ROC-AUC	3.70 (0.92)	-0.89 (0.89)	-3.78 (0.19)	0.57 (0.04)	3.70 (0.85)	4.18 (0.15)	-0.08 (0.06)	4.67 (0.80)
	GNN	Accuracy	3.35 (0.31)	-0.27 (0.02)	0.24 (0.01)	0.92 (0.56)	0.54 (0.03)	0.29 (0.22)	0.86 (0.64)	0.53 (0.26)
		ROC-AUC	2.85 (0.11)	-0.13 (0.00)	-0.12 (0.00)	0.68 (0.33)	0.57 (0.19)	-0.16 (0.00)	0.66 (0.31)	0.80 (0.34)
	Pretrained GNN	Accuracy	4.21 (0.33)	0.11 (0.00)	0.73 (0.09)	0.87 (0.49)	0.62 (0.03)	0.05 (0.03)	0.94 (0.45)	0.35 (0.41)
		ROC-AUC	-0.60 (0.00)	-0.10 (0.01)	0.56 (0.17)	0.59 (0.69)	0.56 (0.23)	0.02 (0.00)	0.66 (0.23)	0.02 (0.00)
DataSAIL	Classical ML	Accuracy	-0.69 (0.04)	-1.82 (0.26)	-0.78 (0.09)	-0.61 (0.07)	-0.81 (0.07)	3.27 (0.82)	1.01 (0.94)	0.44 (0.09)
		ROC-AUC	0.21 (0.00)	0.83 (0.05)	0.60 (0.07)	-0.75 (0.06)	-0.16 (0.01)	-9.69 (0.25)	0.93 (0.84)	0.01 (0.00)
	GNN	Accuracy	-1.08 (0.14)	-1.26 (0.15)	0.51 (0.10)	0.28 (0.08)	-0.32 (0.03)	0.61 (0.27)	0.79 (0.65)	0.84 (0.73)
		ROC-AUC	-1.28 (0.18)	0.23 (0.01)	0.32 (0.08)	0.09 (0.00)	-0.23 (0.01)	0.55 (0.09)	0.33 (0.18)	0.93 (0.75)
	Pretrained GNN	Accuracy	0.30 (0.01)	-0.22 (0.02)	0.44 (0.12)	0.30 (0.10)	0.18 (0.03)	0.53 (0.23)	0.71 (0.54)	0.94 (0.69)
		ROC-AUC	-1.13 (0.12)	1.38 (0.47)	0.38 (0.21)	0.09 (0.01)	0.56 (0.21)	0.54 (0.58)	0.57 (0.47)	0.98 (0.42)

References

(1) Fang, X.; Liu, L.; Lei, J.; He, D.; Zhang, S.; Zhou, J.; Wang, F.; Wu, H.; Wang, H. Geometry-enhanced molecular representation learning for property prediction. *Nature Machine Intelligence* **2022**, 1–8, DOI: 10.1038/s42256-021-00438-4.

(2) Rong, Y.; Bian, Y.; Xu, T.; Xie, W.; Wei, Y.; Huang, W.; Huang, J. Self-Supervised Graph Transformer on Large-Scale Molecular Data. *Advances in Neural Information Processing Systems* **2020**, *33*, DOI: 10.48550/arXiv.2007.02835.

(3) Li, M.; Zhou, J.; Hu, J.; Fan, W.; Zhang, Y.; Gu, Y.; Karypis, G. DGL-LifeSci: An Open-Source Toolkit for Deep Learning on Graphs in Life Science. *ACS omega* **2021**, *6*, 27233–27238, DOI: 10.1021/acsomega.1c04017.

(4) Hu, W.; Liu, B.; Gomes, J.; Zitnik, M.; Liang, P.; Pande, V.; Leskovec, J. Strategies for Pre-Training Graph Neural Networks. Proceedings of the International Conference on Learning Representations. 2020; DOI: 10.48550/arXiv.1905.12265.
